# Chirality in Singlet Fission: Controlling Singlet Fission in Aqueous Nanoparticles of Tetracenedicarboxylic Acid Ion Pairs

**DOI:** 10.1002/advs.202405864

**Published:** 2024-08-13

**Authors:** Ilias Papadopoulos, Joseph Ka‐Ho Hui, Masa‐aki Morikawa, Yasuhito Kawahara, Kenji Kaneko, Kiyoshi Miyata, Ken Onda, Nobuo Kimizuka

**Affiliations:** ^1^ Department of Applied Chemistry Graduate School of Engineering Kyushu University 744 Motooka, Nishi‐ku Fukuoka 819‐0395 Japan; ^2^ Center for Molecular Systems (CMS) Kyushu University 744 Motooka, Nishi‐ku Fukuoka 819‐0395 Japan; ^3^ Department of Materials Science and Engineering Graduate School of Engineering Kyushu University 744 Motooka, Nishi‐ku Fukuoka 819‐0395 Japan; ^4^ Department of Chemistry Faculty of Science Kyushu University 744 Motooka, Nishi‐ku Fukuoka 819‐0395 Japan

**Keywords:** chirality, ion pairs, photochemistry, self‐assembly, singlet fission

## Abstract

The singlet fission characteristics of aqueous nanoparticles, self‐assembled from ion pairs of tetracene dicarboxylic acid and various amines with or without chirality, are thoroughly investigated. The structure of the ammonium molecule, the counterion, is found to play a decisive role in determining the molecular orientation of the ion pairs and its regularity, spectroscopic properties, the strength of the intermolecular coupling between the tetracene chromophores, and the consequent singlet fission process. Using chiral amines has led to the formation of crystalline nanosheets and efficient singlet fission with a triplet quantum yield as high as 133% ±20% and a rate constant of 6.99 × 10^9^ s^−1^. The chiral ion pairs also provide a separation channel to free triplets with yields as high as 33% ±10%. In contrast, nanoparticles with achiral counterions do not show singlet fission, which gave low or high fluorescence quantum yields depending on the size of the counterions. The racemic ion pair produces a correlated triplet pair intermediate by singlet fission, but no decorrelation into two free triplets is observed, as triplet‐triplet annihilation dominates. The introduction of chirality enables higher control over orientation and singlet fission in self‐assembled chromophores. It provides new design guidelines for singlet fission materials.

## Graphical abstract

Chiral molecular assembly promotes singlet fission (SF). Because of efficient SF in chiral chromophore assemblies and decoherence, triplet excitons are generated in aqueous nanoparticles self‐assembled from tetracenedicarboxylic acid derivatives and chiral cations. Achiral ion pairs show no SF, and racemic ion pairs show SF but are deactivated by triplet‐triplet annihilation.

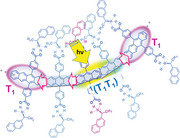

## 1 Introduction

The photophysical phenomenon known as singlet fission (SF) has been the subject of extensive investigations by researchers worldwide for decades.^[^ ^1^, ^2^, ^3^, ^4^ ^]^ The significant attention on the SF process is due to its immense potential in surpassing the current thermodynamic limit of single junction solar cells, known as the Shockley‐Queisser limit, of roughly 32% and raising it to a new theoretical maximum of ≈45%.^[^ ^5^ ^]^ This is due to its ability to energetically down‐convert otherwise unusable high‐energy photons into two excitons of lower energy by effectively splitting singlet excited states (S_1_) into two separated triplet excited states (T_1_+T_1_) (Equation (1)).

(1) $$\begin{array}{c} \;S_{1}{⇄}^{1}\left(T_{1}T_{1}\right){⇄}^{1}\left(T_{1}\cdots T_{1}\right)⇄T_{1}+\;T_{1} \end{array}$$

According to the kinetic three‐step model,^[^ ^6^ ^]^ this transition is intermediated by a coherently coupled triplet pair of singlet multiplicity ^1^(T_1_T_1_) and a spatially separated triplet‐pair ^1^(T_1_⋯T_1_) that has lost electronic coherence but retains the spin coherence. Because these intermediate states are in singlet states overall, the SF process is spin‐allowed and occurs on an ultrafast timescale.^[^ ^1^, ^2^, ^3^, ^6^ ^]^ The complex mechanisms of the SF process, i.e., involvement of ^1^(T_1_T_1_) or other accompanying intermediate states, and the way to control interchromophore coupling that is needed to produce two free triplet excitons efficiently are not yet fully understood, and thus subject to the ongoing research.

For the SF process to transpire in the first place, chromophores need to fulfill several properties and criteria. One primary criterion is the energetic relationship between the excited singlet and triplet states. The energy level of the singlet excited state E(S_1_) should exceed twice the energy level of the triplet excited state E(T_1_), E(S_1_) > 2 × E(T_1_), to serve as the thermodynamic driving force for exothermic SF. Being equal to or slightly above E(S_1_) is also viable for endothermic SF, E(S_1_) ≲ 2 × E(T_1_).^[^ ^1^, ^3^ ^]^ The most well‐known and investigated chromophore representatives for endothermic and exothermic SF are derivatives of tetracene and pentacene, respectively.^[^ ^3^, ^7^, ^8^ ^]^ Another essential energetic requirement is the energy level of higher triplet excited states (T_2_,…T_n_). Their energy levels should most suitably exceed 2 × E(T_1_), to avoid any possibility of their formation via triplet‐triplet annihilation (TTA) and thus allow the generation of multiple individuals (T_1_): E(T_2_, … T_n_) ≫ 2xE(T_1_).^[^ ^3^ ^]^

In addition to energetic requirements, sufficient electronic coupling between the interacting chromophores must be appropriated for SF. The strong interchromophore coupling is a prerequisite for efficient SF to compete with other relaxation events, such as fluorescence. Although such intermolecular coupling can be achieved in solutions with high concentrations of chromophores,^[^ ^9^, ^10^ ^]^ most research focuses on dimer compounds in solution or condensed molecular solids. Covalent molecular dimers or oligomers in solution are models for obtaining a detailed understanding of intramolecular SF processes, and the choice of linkers can control the orientation, spatial overlap, and degree of coupling, allowing fine‐tunning and analysis of their impact on the SF performance and mechanisms.^[^ ^3^, ^11^ ^]^ However, synthesizing covalently linked chromophores requires much effort, and the SF processes in solution are irrelevant to the SF that occurs in their condensed states, the favorable form for applications. In addition, volatile organic solvents make these approaches unsuitable for application.

Achieving efficient SF in molecularly assembled systems for more valuable applications is a crucial direction. To this end, it is essential to understand the relationship between the molecular organization and the rate and yield of SF while controlling various hierarchical structures from the nano‐region to the bulk regime. In molecular crystals, the electronic intermolecular coupling between S_1_ and ^1^(T_1_T_1_) required for SF depends on the local intermolecular geometry of the chromophores. The coupling vanishes for a pair of linear acenes π stacked with C*_2v_* symmetry,^[^ ^1^, ^2^, ^12^, ^13^ ^]^ while the common herringbone alignment is not necessarily an optimized orientation for fast SF.^[^ ^8^ ^]^ Although the strong coupling is desirable to produce ^1^(T_1_T_1_), it is often accompanied by a lowering of the S_1_ energy, which could make SF endothermic. It provides a thermodynamic barrier that hinders the production of independent triplets (T_1_ + T_1_), and also facilitates a deleterious pathway from ^1^(T_1_T_1_) back to the singlet excited or ground state.^[^ ^1^, ^14^ ^]^ Therefore, molecular design principles are needed to precisely control the chromophore organization, which facilitates the electronic dephasing of ^1^(T_1_T_1_) to weakly exchange‐coupled triplet pairs ^1^(T_1_⋯T_1_) and further separation into two independent excited triplets (T_1_ + T_1_). The former process to produce weakly exchange‐coupled ^1^(T_1_⋯T_1_) in parallel molecules has been considered to be promoted by obtaining a mixed spin character of ^1^(T_1_T_1_) and ^5^(T_1_T_1_) under conditions of a weak inter‐triplet coupling regime (***J*** ≪ ***D***), with the quintet acting as an intermediate between ^1^(T_1_T_1_) and (T_1_ + T_1_).^[^ ^13^, ^15^, ^16^ ^]^ Here, ***J*** denotes the inter‐triplet exchange interaction, and ***D*** is the intra‐triplet dipolar interaction.^[^ ^13^, ^15^, ^16^ ^]^ For nonparallel molecules (i.e., with translation + rotation operations), additional singlet‐triplet and quintet‐triplet mixing becomes possible, causing increased singlet‐triplet‐quintet mixing.^[^ ^16^ ^]^ The succeeding separation into a pair of independent triplet excitons (T_1_ + T_1_) will be facilitated by taking full advantage of the entropy gain by diffusing triplet excitons apart, i.e., by rapid triple energy migration via the Dexter mechanism among regularly aligned chromophores.^[^ ^17^ ^]^ However, general molecular design guidelines that satisfy the weak triplet‐triplet exchange interactions and efficient separation of the triplet states have not been obtained.

In this work, we introduced chirality as a new regulator of intermolecular orientation in self‐assemblies to address the challenges of SF, as mentioned above. In general, chromophores in chiral self‐assemblies take a regularly twisted molecular orientation by translational and rotational manipulations characterized by circular dichroism.^[^ ^18^, ^19^, ^20^ ^]^ There are two approaches to introducing chirality into molecular assemblies: covalent and noncovalent. This study adopted the latter supramolecular approach, as it reduces the synthetic effort. Although many reports have been on SF in single‐component systems,^[^ ^21^, ^22^, ^23^, ^24^, ^25^, ^26^, ^27^ ^]^ SF based on supramolecular self‐assemblies formed from different subunits is surprisingly poorly reported.^[^ ^28^ ^]^ By adequately designing the noncovalent intermolecular interactions between SF‐exhibiting chromophores and chiral components, it is possible to achieve the chiral and regular molecular orientation, i.e., a relative arrangement of π electron systems without translational symmetry. We expected that such nonparallel, twisted chiral chromophore orientations with controlled interchromophore distances could generate weak exchange coupling, leading to the mixed spin state that promotes SF.^[^ ^15^, ^16^ ^]^ Furthermore, by introducing regular, chiral chromophore arrays, rapid triplet energy migration will be achieved, which is advantageous for separating the triplets produced. Although some examples of covalent chiral molecular dimers of SF chromophores have been reported, they are studies on molecularly isolated species in solution.^[^ ^29^, ^30^ ^]^ To our knowledge, the chiral supramolecular assembly of SF chromophores has not been reported.

**Figure 1:**
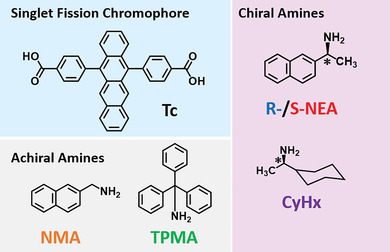
Chemical structures of the dicarboxylic acid tetracene SF chromophore (Tc), chiral amines (R)/(S)‐1‐(2‐Naphthyl)ethylamine (**R‐NEA**/**S‐NEA**), (R)‐(‐)−1‐Cyclohexylethylamine (**CyHx**), and achiral amines triphenyl methylamine (**TPMA**), naphthalen‐2‐ylmethanamine (**NMA**).

We employed aqueous self‐assembly of ion pairs to prepare chiral molecular assemblies,^[^ ^20^, ^31^, ^32^, ^33^ ^]^ which harnesses electrostatic interactions and hydrogen bonding in the course of hydrophobic self‐assembly between chiral cations with anionic Tc chromophores. This simple approach allows us to introduce chiral molecular arrangements into chromophores.^[^ ^20^, ^31^, ^32^, ^33^ ^]^ We prepared aqueous nanoparticles (**NP**s) because they facilitate spectroscopic characterizations, including CD spectra, and allow us future wet processing into solid films. As a chromophore for investigating SF phenomena in chiral molecular assemblies, we selected a 5,12‐ diphenyl‐tetracene‐based dicarboxylic acid (**Tc**, **Figure** **1**). Tetracene has a triplet energy level of ca. 1.25 eV,^[^ ^34^ ^]^ and its SF characteristics have been widely investigated in the solid state^[^ ^7^, ^35^, ^36^, ^37^, ^38^ ^]^ and solar cell applications.^[^ ^34^, ^39^ ^]^ Meanwhile, the SF process of tetracene is endothermic by 0.2 eV and exhibits slow dynamics (10–100 ps).^[^ ^17^, ^34^, ^39^ ^]^ These features are considered suitable to illustrate the role of the counterions' size, shape, and chirality in determining the molecular organization, which governs the photo‐relaxation processes of the **Tc** chromophores, particularly in the triplet energy migration^[^ ^17^, ^40^, ^41^ ^]^ and SF processes.

As chiral counterions, (R) and (S)‐1‐(2‐Naphthyl) ethylamine (**R‐NEA/S‐NEA**) and (R)(‐)−1‐Cyclohexyl‐ethylamine (**CyHx**) were employed (Figure 1). A racemic form **(Rac)** consisting of equimolar **R‐NEA** and **S‐NEA** was also introduced as the counter ion to demonstrate the importance of enantiomeric purity. As achiral cations, triphenyl methylamine (**TPMA**) and naphthalen‐2‐ylmethanamine (**NMA**) were used, respectively. The SF properties of colloidal **NP**s have been reported for 5,12‐diphenyltetracene^[^ ^42^, ^43^ ^]^ and 1,11‐diphenyltetracene carboxylic acids,^[^ ^44^, ^45^ ^]^ where the triplet yield of the former **NP**s (75%)^[^ ^43^ ^]^ has been shown to be inferior to that of bulk amorphous films (122%).^[^ ^42^ ^]^ However, supramolecular control of the SF process in **NP**s formed by chiral molecular assembly has been unprecedented. This study aims to shed light on the insights and role of chirality in self‐assembly‐based intermolecular SF.

## 2 Results and Discussion

### 2.1 Characterization of Ion‐Pair Nanoparticles

The formation of pairs of tetracene dicarboxylate (**Tc^2−^**)‐ammonium ions in the initial step was confirmed by changes in the stretching vibrations of C═O in the FT‐IR spectra (Figures S1–S6, Supporting Information). We noticed that the deprotonated monomeric form of **Tc^2−^**, obtained by KOH, appeared unstable in THF, producing new absorption and fluorescence peaks unrelated to tetracene after one day (Figure S7, Supporting Information). In contrast, the ammonium salts of **Tc^2−^** dispersed in water are substantially more stable, indicating that molecular self‐assembly in water contributes to increased chemical stability. We assume that the observed stabilization is due to the hydrophobic microenvironment and the contribution of hydrogen bonding within the **NP**s.

Dynamic light scattering (DLS) measurements were performed to gain insight into the size distributions of the nanoparticles (Figure S8, Supporting Information). The single‐component **Tc_NP**s showed rather monodisperse nanoparticles with average sizes between 50 – 70 nm. Meanwhile, binary **Tc^2−^**‐ammonium nanoparticles showed generally larger particle sizes than **Tc_NP**s, and their distribution was more polydisperse. The Zeta potential of **Tc_NP**s exhibited a large negative potential of ≈−60 mV, indicating that the carboxyl groups of **Tc^2−^** on the surface of the nanoparticle are proton‐dissociated, and the observed negative charge contributed to the stability of the aqueous dispersion (Figure S9a, Supporting Information). On the other hand, aqueous dispersions of **Tc^2−^** with various ammonium counterions revealed lower negative Zeta potentials of ≈−30 mV (Figure S9b–g, Supporting Information). This reflects the reduced anion charge density at the nanoparticle‐water interface, which is reasonable given that the nanoparticles consist of ion pairs and hydrogen bonding formed between the carboxyl groups of **Tc^2−^** and ammonium ions.

**Figure 2:**
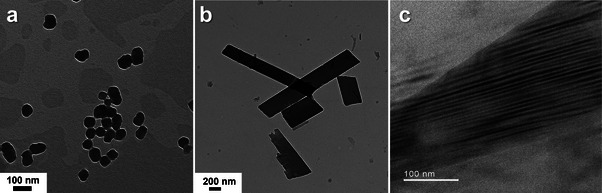
TEM images of aqueous **NP**s. a) **Tc_NP**s, scale, 100 nm. b) **Tc_R‐NEA_NP**s, scale, 200 nm. c) high‐resolution TEM for **Tc_R‐NEA_NP**s, scale, 100 nm.

Transmission electron microscopy (TEM, **Figure** **2**; Figure S10, Supporting Information) further confirmed the nanoparticle morphology. TEM images of **Tc_NP**s revealed monodisperse spherical particles (Figure 2a) with sizes consistent with the DLS data (Figure S8, Supporting Information). The ion‐complexed **NP**s on the other side revealed polydisperse structures (Figure S10b–g, Supporting Information). Ill‐defined shapes were observed in the case of **Tc_TPMA_NP**s, **Tc_NMA_NP**s, and **Tc_CyHx_NP**s.

Meanwhile, the use of the chiral, compact **R‐/S‐NEA** in **Tc_R‐/S‐NEA_NP**s led to a sheet‐like morphology (Figure 2b). In high‐resolution TEM images of **Tc**_**S‐NEA_NP**s, ordered molecular layers with a thickness of ≈3 Å were observed within these sheets, suggesting some degree of crystallinity (Figure 2c; Figure S11a–c, Supporting Information). These observations were further corroborated via grazing‐incidents small‐angle X‐ray scattering (GI‐SAXS) analysis (Figure S11d, Supporting Information). The racemate mixture also gave a sheet‐like morphology (Figure S10g, Supporting Information). Overall, these observations demonstrated that the electrostatic complexation of **Tc** and the chosen amine significantly affects the self‐assembly and morphology of aqueous **NP**s.

### 2.2 Photophysical Characterization with Steady‐State Spectroscopy

**Figure 3:**
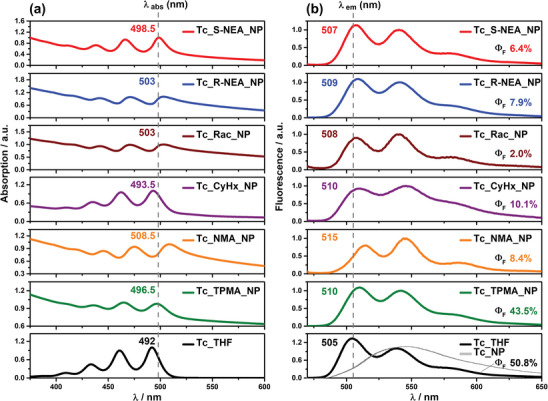
Steady‐state absorption a) and fluorescence b) spectra and quantum yield (Φ_F_) of **Tc** in THF (black), **Tc_TPMA_NPs** (green), **Tc_NMA_NPs** (orange), **Tc‐CyHx_NPs** (purple), **Tc_Rac_NPs** (brown), **Tc_R‐NEA_NPs** (blue), and **Tc_S‐NEA_NPs**(red) in water. The fluorescence spectrum of aqueous **Tc_NPs** (gray) is also shown in the bottom right‐hand figure. The fluorescence spectra were recorded at an excitation wavelength of 460 nm.

First, we investigated the spectroscopic steady‐state characteristics of **Tc**, **TPMA**, **NMA**, **CyHx**, **R‐**/**S‐NEA,** and **Rac** in THF solutions. Steady‐state absorption measurements of **TPMA**, **NMA**, **CyHx**, **R‐**/**S‐NEA,** and **Rac** revealed only absorption bands in the far blue to ultraviolet part of the visible spectrum between 200 and 300 nm (Figure S12, Supporting Information). For **Tc**, the familiar set of absorption bands with transition moments in the molecular short‐axis direction (^1^L_a_ ←^1^A transition, 400–550 nm) and in the long‐axis direction (^1^B ←^1^A transition, 200–350 nm)^[^ ^43^ ^]^ were observed (Figure S13, Supporting Information). Steady‐state fluorescence measurements with an excitation wavelength of 460 nm yielded two prominent **Tc** fluorescence bands at ≈505 and 540 nm (**Figure** **3**b, bottom; Figure S13, Supporting Information). No fluorescence could be discerned under this condition for **TPMA**, **NMA**, **CyHx**, **R‐/S‐NEA**, and **Rac**. In THF solutions, the **Tc^2−^**/ammonium complexes yielded spectra identical to those of pure **Tc**, indicating that the complexes are molecularly dispersed in THF without forming aggregates (Figure S14 and Table S1, Supporting Information).

On the other hand, the results obtained for the aqueous **NP**s painted a different picture. Steady‐state absorption spectra of all **NP**s featured more‐or‐less red‐shifted and broadened absorption peaks, indicative of aggregation and the consequent ground‐state interactions between the tetracene units (Figure 3a). The absorption of aqueous **Tc_NP**s containing no counterions showed a red shift in the 0‐0 absorption peak (492 nm in THF, 502.5 nm in aqueous **NP**s, Figure S15a, Supporting Information). In fluorescence spectra, a significant red shift and broadening with complete loss of fine structure were observed, characteristic of excimer emission (Figure 3b, bottom (gray); Figure S15b, Supporting Information). Interestingly, such a broad excimer emission was not observed for aqueous ion‐paired **NP**s, which all showed structured emissions. These data indicate that the ion‐pairing affects the molecular orientation of **Tc** and suppresses excimer formation, which traps excited states and is detrimental to SF.^[^ ^46^ ^]^

In the case of **Tc_TPMA_NP**s and **Tc_R‐/S‐NEA_NP**s, the shape and fine‐structure of the fluorescence remained the same as in **Tc** molecularly dissolved in THF, with only minor redshifts and slight broadening but a more substantial weighting of the 0–1 emission peak (Figure 3b). Meanwhile, in **Tc_NMA_NP**s, both absorption and emission peaks are most red‐shifted compared to other ion‐paired **NP**s, and two prominent fluorescence peak intensities are inverted compared to **Tc_TPMA_NP**s and **Tc_R‐/S‐NEA_NP**s. Both **NMA** and **R‐/S‐NEA** contain naphthalene chromophores and have similar molecular sizes, but the observed considerable red shifts in the absorption and fluorescence spectra for the achiral **Tc_NMA_NP**s (Figure 3; and Table S2, Supporting Information) indicate more extensive interchromophore interactions. **Tc_CyHx_NP**s show relatively broadened emission peaks, suggesting a small excimer emission component, with the inversion of two prominent fluorescence peak intensities like **Tc_NMA_NPs**. Thus, the molecular orientation of **Tc** in ion‐paired **NP**s, which are reflected in absorption and fluorescence spectra, varies depending on the size and shape of the counterions. We note that the red shift of the absorption and fluorescence peaks of ion‐pair **NP**s is relatively weak compared to **Tc_NP**s, except for **Tc**_**NMA_NP**s, indicating that the orientation and interaction of **Tc** chromophores within ion‐pair **NP**s are controlled. It is interesting that the chiral **Tc_R‐/S‐NEA_NP**s exhibit fluorescence with vibrational structures similar to those of the monomeric **Tc** in THF.

Notably, the fluorescence quantum yields (Φ_F_) of aqueous **NP**s were also highly dependent on the chemical structure of ammonium ions. For **Tc** molecularly dissolved in THF, a Φ_F_ of as high as 50.8% was obtained, and similarly high Φ_F_’s were observed for all the **Tc**‐ammonium salts dissolved in THF (Figure 3b; and Table S1, Supporting Information). Meanwhile, the broad excimer emission observed for aqueous **Tc_NP**s gave significantly quenched Φ_F_ at ≈2.7% (Table S2, Supporting Information), in line with previous reports on tetracene‐based nanoparticles. On the other hand, applying the bulky, achiral **TPMA** yielded a minor quenched Φ_F_ of 43.5% in aqueous **Tc_TPMA_NP**s, suggesting small interactions (Figure 3b; and Table S2, Supporting Information). In comparison, **Tc_R‐/S‐NEA_NP**s showed a Φ_F_ value of 7.9% and 6.4%, a quenching on par with **Tc_NP**s, highlighting the stronger interchromophore interactions in chiral **Tc_R‐/S‐NEA_NP**s. Furthermore, the Φ_F_’s of **Tc_NMA_NP**s, **Tc_CyHx_NP**s, and **Tc_Rac_NP**s were also heavily quenched. However, when considered in conjunction with the differences in the fluorescence spectra as described above, the nature of this quenching appears to be different from the **Tc_R‐/S‐NEA_NP**s (Figure 3b). Overall, the molecular packing within the nanoparticles and the type of interaction, as reflected in the absorption/fluorescence spectra and fluorescence quantum yields, are significantly controllable by choice of countercations.

**Figure 4:**
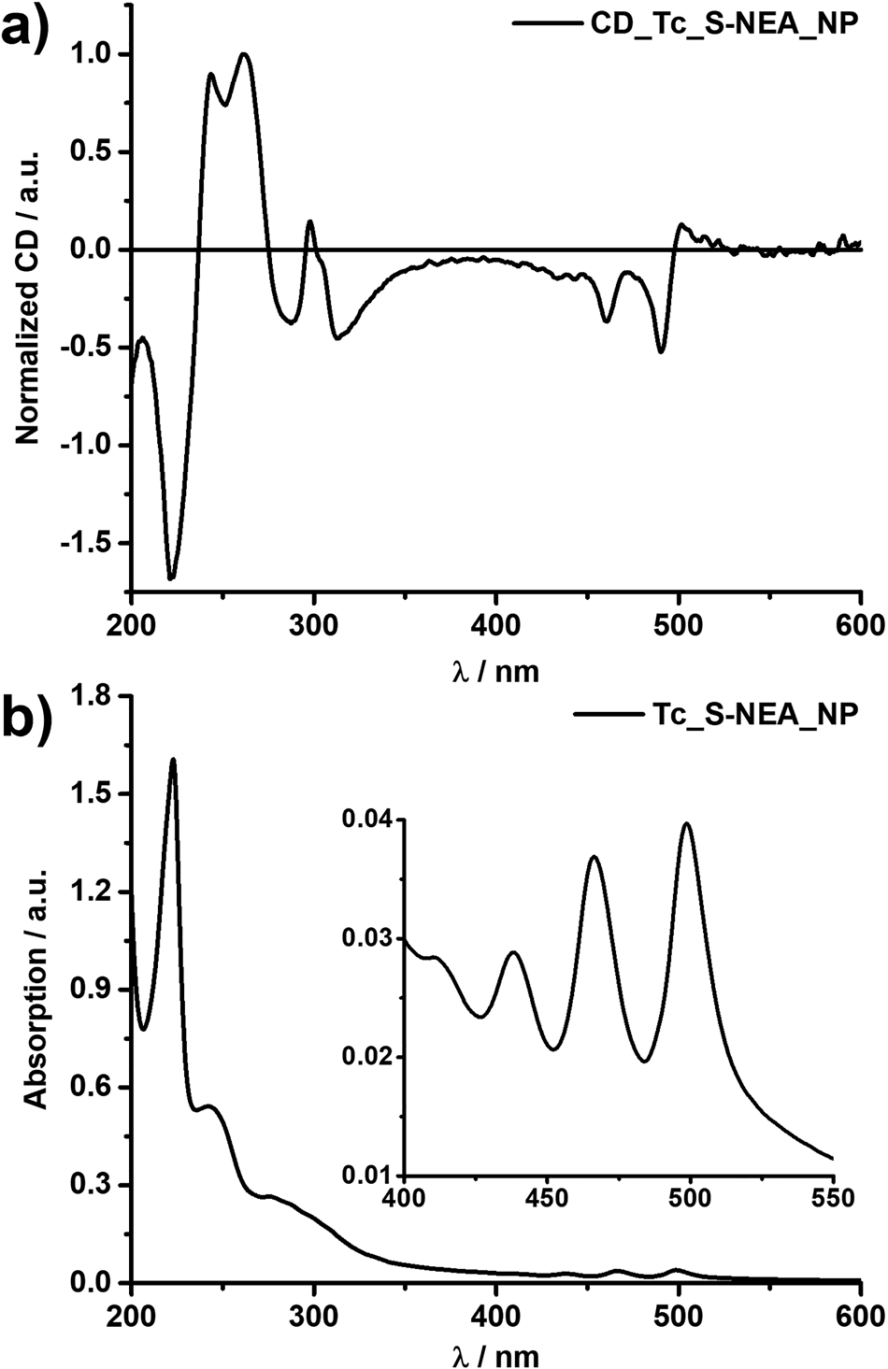
Circular dichroism (CD) spectra a) and corresponding absorption spectra b) of aqueous **Tc_S‐NEA_NPs** ([**Tc**] ≈ 0.1 mm).

Circular dichroism (CD) spectroscopy measurements were conducted to examine the influence of chiral counterions on tetracene chromophores. As expected, reference measurements for **Tc**, **TPMA**, **NMA, Rac**, **Tc_TPMA_NP**s, **Tc_NMA_NP**s, and **Tc_Rac_NP**s did not show CD signals (Figures S16–S22, Supporting Information), while chiral **R‐**/**S‐NEA** and **CyHx** showed cotton effects at ≈200‐250 nm (Figures S23, S25, and S27, Supporting Information). In contrast, **Tc_R‐**/**S‐NEA_NP**s revealed more intense and well‐resolved CD spectra with cotton effects in the UV and vis (450‐500 nm) regimes (**Figure** **4**; Figures S26, S28, and S29, Supporting Information). **R‐**/**S‐NEA** does not have absorption in the vis‐region (Figures S25 and S27, Supporting Information), and the observed CD signals in the absorption band of **Tc** are assigned to the induced CD for tetracene chromophores. They reflect the chiral molecular orientation of **Tc,** directed by the electrostatic interactions and hydrogen bonding with chiral **R‐**/**S‐NEA** in **NP**s. The CD spectra in the UV region show a complicated pattern that appears to be composed of exciton coupling between naphthalene chromophores of **R‐**/**S‐NEA** assembled in **NP**s (Figure 4; Figures S26, S28, and S29, Supporting Information). These results indicate that the intermolecular interactions between **R‐/S‐NEA** and **Tc** within the **NP**s cause chiral, i.e., twisted molecular orientations between the **Tc** molecules, significantly reducing Φ_F_. We note that such massive quenching was absent for aqueous nanoparticles of **Tc_TPMA_NP**s paired with bulky achiral ions (Figure 3b; and Table S2, Supporting Information). The CD spectrum of **Tc_CyHx_NP**s also revealed weak induced CD signals in the vis‐region attributed to **Tc** (Figure S24, Supporting Information). Their overall intensity was much lower compared to **Tc_R**‐/**S**‐**NEA_NP**s, indicating a lower degree of molecular orientational order of **Tc^2−^** with the **CyHx** cation, which is supported by the broadened emission spectrum (Figure 3b) and undeveloped aggregates observed in TEM (Figure S10d, Supporting Information). The **CyHx** cation likely took a non‐flat, flexible conformation, and the ion pair with **Tc** could not ensure sufficient intermolecular interactions to form a regular molecular alignment.

### 2.3 Time‐Resolved Spectroscopy

**Figure 5:**
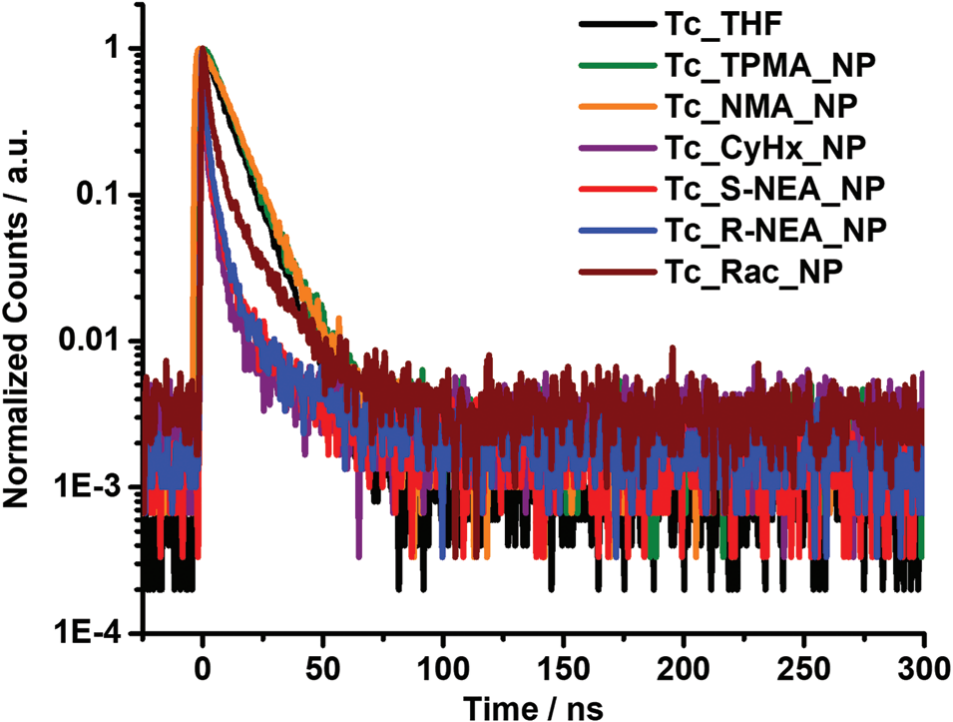
Normalized time‐correlated single photon counting (TCSPC) fluorescence time profiles of **Tc** in THF (black), **Tc_TPMA_NPs** (green), **Tc_NMA_NPs** (orange), **Tc‐CyHx_NPs** (purple), **Tc_S‐NEA_NPs** (red), **Tc_R‐NEA_NPs** (blue), and **Tc_Rac_NPs** (brown) in water, respectively.

After elucidating the steady‐state properties of the nanoparticles, their excited state dynamics were investigated. First, the fluorescence lifetimes were investigated via time‐correlated single photon counting (TCSPC). The TCSPC measurement of **Tc** in THF was subject to a mono‐exponential decay with a lifetime of 9.61 ns (**Figure** **5**; and Table S3, Supporting Information). The highly fluorescent **Tc_TPMA_NP**s also featured a mono‐exponential decay and a similar lifetime of 9.33 ns. The high Φ_F_ and long fluorescence lifetime strongly indicate that the tetracene units complexed with bulky TPMA counterions do not experience sufficient intermolecular coupling within nanoparticles and are not subject to SF.

**Tc_NMA_NP**s with achiral **NMA** as the counterion similarly exhibited a mono‐exponential decay with a lifetime of 10.35 ns. Although **Tc_CyHx_NPs** and **Tc_Rac_NPs** showed emission spectra similar to **Tc_NMA_NPs**, both featured a bi‐exponential decay, including a short‐lived component, hundreds of picoseconds, and a long‐lived component comparable to **Tc**. Finally, the **Tc_R‐**/**S‐NEA_NP**s also showed lifetimes similar to those of **Tc_CyHx_NPs** and **Tc_Rac_NPs**, requiring two exponential fits (Figure 5; and Table S3, Supporting Information). The first, short‐lived major component likely relates to the strongly quenched fluorescence of the singlet excited state of tetracene due to additional non‐radiative decay pathways via the SF or other aggregation‐related processes. The slow decay of the minor second component for **Tc_R‐**/**S‐NEA_NP**s may be due to the contribution of minuscule amounts of tetracene molecules bound at the water‐nanoparticle interface.

Then, we examined the decay dynamics of the excited states using time‐resolved transient absorption spectroscopy on the femto‐ (fsTAS; up to 1200 ps; λ_ex _= 400 nm) and nanosecond (nsTAS; up to 1 µs; λ_ex _= 410 nm) time scale. The raw TAS data were examined using a single wavelength and global analysis using the GloTarAn software package. Starting with fsTAS of the reference, **Tc** in THF revealed the instantaneous population of its singlet excited state (S_1_) after photoexcitation, featuring sharp maxima at roughly 475 and 525 nm, and a broad maximum centered at 625 nm (Figure S30, Supporting Information). They are accompanied by minima at 495 and 540 nm, corresponding to ground‐state bleaching (GSB) and stimulated emission (SE), respectively. These features are consistent with previous reports.^[^ ^47^ ^]^ The complete deactivation process was not finished on this timescale, and no further transients could be discerned. Systematic measurements of other **Tc**_ammonium salts in THF yielded the same observations (Figures S30–S43, Supporting Information).

Next, we will discuss the fsTAS results for the achiral **Tc_TPMA_NP**s in water (Figure S44, Supporting Information). The same broad maximum between 600 and 700 nm was discernable together with GSB and SE, as found in **Tc** in THF (Figure S30, Supporting Information), inferring the formation of (S_1_). Furthermore, **Tc_TPMA_NPs** followed the same deactivation dynamics as the **Tc** reference in THF, with no signs of ^1^(T_1_T_1_) population via SF. Again, full ground‐state recovery was not reached on this timescale. This result corresponds well with the high fluorescence quantum yield of aqueous **Tc_TMPA_NP**s (Figure 3b), indicating the absence of favorable electronic intermolecular coupling between **Tc** chromophores placed spatially apart in **Tc_TMPA_NP**s.

In the case of aqueous **Tc_NMA_NP**s, which contain compact, achiral **NMA** as counterions, a low fluorescence quantum yield of 8.4% was observed, as mentioned earlier. Despite the observed fluorescence quenching, the aqueous **Tc_NMA_NP**s showed fsTAS results similar to those of **Tc_TPMA_NPs** with no signs of SF (Figure S45, Supporting Information). As discussed, **Tc_NMA‐NP**s showed the most red‐shifted absorption and fluorescence spectra (Figure 3), indicating electronic interactions among the **Tc** chromophores. It suggests lowering the S_1_ energy, which would have made SF more endothermic and unfavorable. Also, it is likely that **Tc_NMA‐NP**s lack long‐ranged molecular order, as inferred from the irregular aggregate morphology observed by TEM (Figure S10c, Supporting Information). The fluorescence quenching in **Tc_NMA‐NP**s may be due to the predominant singlet energy transfer to non‐radiative defect sites.

Switching over to the aqueous **NP**s with chiral ammonium complexes, we first want to focus on the bulky **CyHx** in aqueous **Tc_CyHx_NP**s (Figure S46, Supporting Information). As mentioned, these aqueous **NP**s exhibited irregular morphology and a broadened fluorescence spectrum with lower fluorescence quantum yield (Figure 3b), suggesting the presence of excimer‐forming sites and defects that serve as energy traps for singlets. In fsTAS measurements, no implications of a triplet excited state could be found for **Tc_CyHx_NP**s on the femtosecond time scale, while the fsTAS raw data required a two‐species kinetic model (Figure S46d, Supporting Information). The short‐lived component (black) most likely relates to the vibrational relaxation/inhomogeneity of the flexible cyclohexane ring of the ammonium, while the long‐lived component (red) stems from the incomplete deactivation of this relaxed singlet excited state. These assignments will be discussed in the following description for aqueous **Tc_R‐/S‐NEA_NP**s.

**Figure 6:**
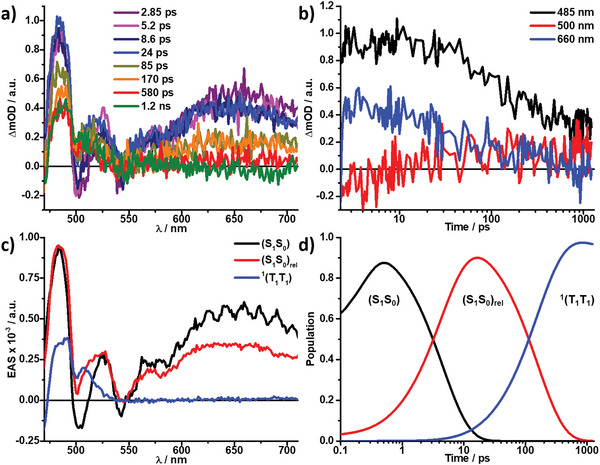
a) Femtosecond transient absorption spectra (λ_ex_ = 400 nm) of **Tc_S‐NEA_NPs** in water at the indicated time delays, together with b) the respective time absorption profiles at the indicated wavelengths. c) Deconvoluted evolution‐associated spectra (EAS) showcasing the initial hot singlet excited state (S_1_S_0_) (black), solvent and vibrational relaxed singlet excited state (S_1_S_0_)_rel_ (red), and singlet correlated triplet pair ^1^(T_1_T_1_) (blue) as obtained from global analysis. d) Respective population kinetics of c).

As mentioned, chiral **Tc_R‐/S‐NEA_NP**s in water revealed regular molecular alignments, as indicated by TEM (Figure 2b,c) and CD spectra (Figure 4). The fsTAS measurements for both **Tc_R‐/S‐NEA_NP**s in water showed striking differences from those for the other ion‐paired systems. The raw data from fsTAS for these dispersions best fit a three‐species kinetic model (**Figure** **6**; Figure S47, Supporting Information). The first observed species in **Tc_R‐/S‐NEA_NP**s also featured all the maxima as mentioned earlier together with the GSB, but now lack the intense SE minimum. The second observed species was formed within 5 ps and consisted of similar transients, with notable differences being the depletion of the GSB to now positive values and blue shifts of the broad band from 630 to 670 nm (Figure S47a, Supporting Information). Considering that the immediately formed excited state is likely to be more polar than the corresponding ground state and the similarities of both spectra, we infer that the first and second species are related to initially photo‐excited hot singlet excited states (S_1_S_0_) and after vibrational and solvent relaxation (S_1_S_0_)_rel_. These conclusions also agree with the literature.^[^ ^48^, ^49^, ^50^, ^51^ ^]^ The third discernable species on this timescale was vastly different from the previous two. Its fast population concluded after roughly 160 ps and exhibited two maxima at ≈490 and 515 nm and the absence of the broad maximum or any GSB (Figure 6c; Figure S47c and Table S4, Supporting Information). This fast formation and a comparison with the literature confirmed that the third species was the singlet correlated triplet pair ^1^(T_1_T_1_) populated via SF, and the chirality promoted the SF process.

It is worth noting that the SF was observed for chiral ion‐paired **NP**s with **S‐/R‐NEA** in which **Tc** chromophores take on a chiral, regular molecular geometry. The triplet quantum yield (TQY) for the transition from (S_1_S_0_)_rel_ to ^1^(T_1_T_1_) was determined using the ground state bleaching method, as described in the literature,^[^ ^44^, ^45^, ^52^, ^53^ ^]^ leading to a TQY of ≈133% ±20% for **Tc_S‐NEA_NP**s and 129% ±20% for **Tc_R‐NEA_NP**s (see Figures S53 and S54, Supporting Information for details). As will be described later, nsTAS measurements revealed that besides strong interaction for the population of ^1^(T_1_T_1_), these two chiral ion pairs also provided a pathway for subsequent decoherence into (T_1_+T_1_) with an efficiency of ≈30% ± 10%. In these chiral systems, the recovery of the ground state via triplet‐triplet annihilation of ^1^(T_1_T_1_) was not observed on the fsTAS time scale.

Lastly, to demonstrate that a single chirality is essential in the observed SF in chiral **Tc_R‐/S‐NEA_NP**s, we investigated fsTAS measurements for racemic **Tc_Rac_NP**s in water, which contain equimolar **R‐NEA** and **S‐NEA** as counterions (Figure S48, Supporting Information). Photoexcitation of **Tc_Rac_NP**s led to two distinguishable features from the GloTarAn global analysis. The first species resembled the singlet excited state, as was discussed in the prior **Tc**‐ammonium ion‐paired **NP**s; however, they lack the GSB signals, possibly because of the overlap of stronger excited state absorptions with the GSB. This species is then replaced within 43 ps with the second species resembling the triplet excited state of **Tc**, including the excited state absorption (ESA) at ≈490 and 516 nm and a lack of the broad ESA ≈625 nm (Figure S48a, Supporting Information). Again, differences could be noted, as seen with the inversion of the intensity ratio of the two maxima at 490 and 513–516 nm. Notably, ground‐state recovery within 450 ps was further observed (Figure S48a, Supporting Information), being a major distinguishable factor in the excited state properties of the racemate compared to the chiral **Tc_R‐/S‐NEA_NP**s (Table S4, Supporting Information). These differences in fsTAS paired with the altered shape of the **Tc_Rac_NP**s steady‐state fluorescence suggest that the molecular assembly and interchromophore interactions within **Tc_Rac_NP**s are different from **Tc_R‐/S‐NEA_NP**s, but still enable strong intermolecular coupling for SF that lead to a slower but efficient triplet pair formation with a TQY of 140% ± 20% (Figure S55, Supporting Information). However, it should be noted that the triplet pairs formed in racemic **NP**s were subjected to a rapid triplet‐triplet annihilation(TTA) on the fsTAS timescale due to interferences of the two included chiralities within the aqueous **NP**s. In the racemate **NP**s, the long‐range molecular order would be inferior to that of chiral nanoparticles. This highlights the need for pure chirality and long‐range molecular order for efficient SF and prevention of TTA, which was further investigated in the nsTAS measurements. The optical relaxation processes observed for the aqueous **Tc** nanoparticles described above are schematically illustrated in Figure 8.

The nsTAS measurements allowed us to observe each sample's complete excited state deactivation. NsTAS assays of the **Tc** and all the **Tc^2−^** _ammonium salts monomerically dissolved in THF led to the same singlet excited state decay within roughly 14 ns and no population of a triplet excited state. (Figures S37–S43 and Table S4, Supporting Information). The same was true for aqueous **Tc_TPMA_NPs**, **Tc_NMA_NPs**, and **Tc_CyHx_NPs**, which did not show any SF behavior in fsTAS measurements and only revealed ground state recovery from (S_1_) in nsTAS (Figures S49–S51 and Table S4, Supporting Information).

**Figure 7:**
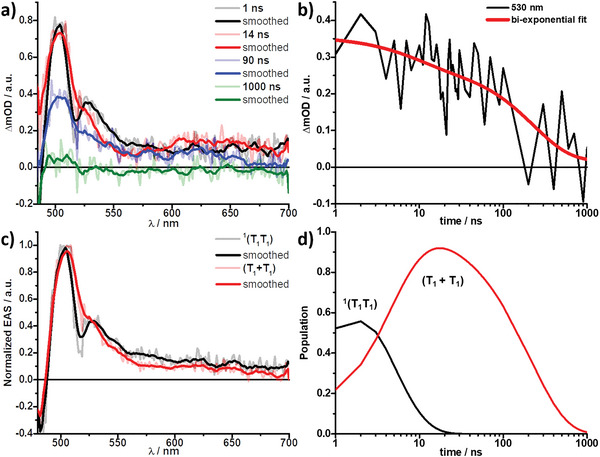
Nanosecond transient absorption spectra (λ_ex_ = 410 nm) of **Tc_S NEA_NPs** in water at the indicated time delays, together with b) the respective time absorption profile and fit at the indicated wavelength. c) Deconvoluted normalized evolution‐associated spectra (EAS) showcasing the singlet correlated triplet pair ^1^(T_1_T_1_) (black) and uncorrelated triplet excited states (T_1_+T_1_) (red) as obtained from global analysis. d) Respective population kinetics of c).

**Figure 8:**
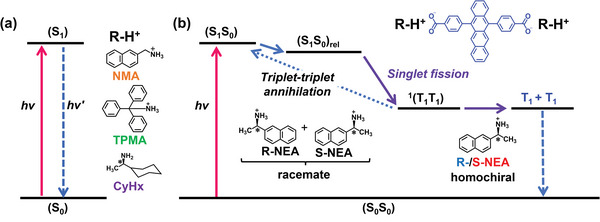
Schematic illustration of the photo‐relaxation processes of the excited states of aqueous ion‐paired **Tc NP**s. a) In aqueous **NP**s with counterions of **NMA**, **TPMA** and **CyHx**, SF is not observed. b) Ion‐paired **NP**s with chiral **S‐NEA** and **R‐NEA** counterions exhibit an SF process to a coherently coupled triplet pair ^1^(T_1_T_1_), respectively, which subsequently undergoes dephasing and separation into two uncorrelated triplet excited states T_1_ + T_1_. The **NP**s with racemate counterions slower show SF to the triplet pair ^1^(T_1_T_1_), which, however, undergoes rapid triplet‐triplet annihilation (TTA) without decoherence into the uncorrelated triplet states. These observations indicate that the chiral molecular orientation and ordered self‐assembly induced by homochiral counterions of appropriate size promote SF.

Meanwhile, these situations are quite different in aqueous **Tc_R‐/S‐NEA_NP**s, which did reveal efficient SF in fsTAS (Figures 6 and 8; Figure S47, Supporting Information). After ≈10 ns, the singlet correlated triplet pair of **Tc_R‐/S‐NEA_NP**s (in red) transitioned into a species sharing very similar characteristics in 90 ns (in blue), which have lost their fine structure and are broadened overall (**Figure** **7**a). Within 300 ns, this second species decayed back to the ground state. We infer the transition to this second species to the decoherence of ^1^(T_1_T_1_) into the excited states of uncorrelated triplets (T_1_+T_1_) (Figure 7c,d; Figure S52 and Table S4, Supporting Information), whose appearance and lifetime are also in agreement with the literature.^[^ ^44^, ^45^ ^]^ The calculated yield of decoupled and free triplets, again using the ground state bleaching method as described in the literature,^[^ ^44^, ^45^, ^52^, ^53^ ^]^ were 33% ± 10% in **Tc_S‐NEA_NP**s and 32% ±10% in **Tc_R‐NEA_NP**s (Figures S56 and S57, Supporting Information).

**Figure** **8** schematically represents the results obtained in this study. Only these aqueous **NP**s with compact, chiral amines yielded SF characteristics, highlighting that size, shape, and chiral purity are key factors for the efficient formation of ^1^(T_1_T_1_) and further channel for decoupling and separation into free triplets (T_1_ + T_1_). When bulky or achiral counterions are employed, SF is not observable. Introducing chirality in chromophore self‐assembled systems allows fine control over chromophore organization and their interactions, including triplet‐triplet exchange interactions.

**Figure 9:**
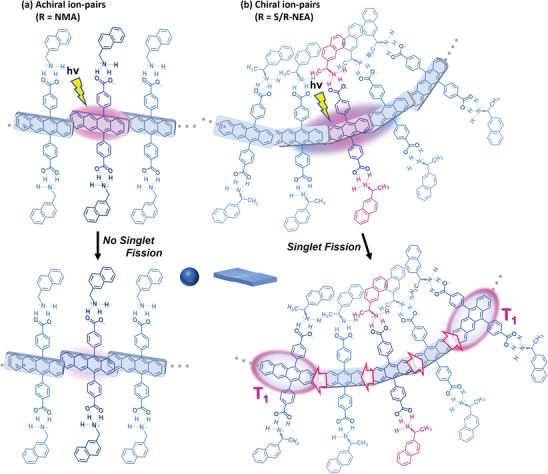
Schematic illustration of the relaxation processes of the excited states of aqueous **Tc** nanoparticles. a) aqueous **NP**s of achiral ion pairs (R = **NMA, TPMA**) do not show SF. In **Tc**_**NMA_NP**s, the S_1_ energy level is lowered due to chromophore interactions, as shown by pronounced red shifts in absorption and fluorescence. This makes the SF process more endothermic and thus unfavorable. In the case of **Tc**_**TPMA_NP**s, tetracene units are separated by bulky **TPMA** ions and do not show the electronic intermolecular coupling required for SF. **CyHx** is a chiral anion; however, the aqueous **Tc_CyHx** did not show SF as described in the text. b) aqueous **NP**s (nanosheets) with chiral **S‐NEA** and **R‐NEA** counterions exhibit an SF process, respectively. These observations indicate that SF is promoted for chiral molecular self‐assemblies with chiral counterions of appropriate size and shape. The translational symmetry of the chromophore is broken by introducing chirality, inducing a nonparallel twist orientation. It would enhance the mixed spin character in weakly exchange coupled triplet pairs ^1^(T_1_⋯T_1_), and the ordered arrangement of tetracene chromophores within the nanosheets facilitates the separation of the correlated triplet pair via triplet energy migration. Note that this figure is only a conceptual cartoon highlighting the twist orientation of the tetracene long axis.

As mentioned in the introduction, the critical factors for achieving SF in the molecular organization are realizing both the enhanced mixed spin character in weakly exchange coupled triplet pairs ^1^(T_1_⋯T_1_) and ordered molecular assembly that can separate a pair of independent triplet excitons (T_1_ + T_1_) through rapid triplet energy migration. The chiral chromophore self‐assembly proposed in this study can simultaneously satisfy these requirements – controlled distances, nonparallel orientations, the favourable overlap of the π‐electron clouds of tetracene units, and their long‐range ordered self‐assembly. Among a series of **Tc**‐containing supramolecular **NP**s developed in this study, SF was observed only for chiral **Tc_R‐/S‐NEA_NP**s, which supports the validity of our idea. The importance of chiral molecular orientation in these ion‐paired molecular assemblies is schematically illustrated in **Figure** **9** for (a) achiral **Tc_NMA_NP**s and (b) **Tc_NEA_NP**s, respectively, in terms of the conceptual cartoon of molecular orientation and the observed photorelaxation processes. Proper design of chiral molecular assemblies allows for compelling SF and separation into uncoupled triplet states.

## 3 Conclusion

In conclusion, the effect of chirality on the photo‐relaxation and SF characteristics was investigated for aqueous ion‐pair nanoparticles. They were self‐assembled from dicarboxylic acid‐functionalized tetracene (**Tc**) and various amines of different sizes and molecular shapes. The size, shape, and chirality of counterions significantly impacted the molecular organization, absorption, and fluorescence spectral characteristics and photorelaxation processes. The importance of the present study is fourfold.

First, the ion pairing with organic cations suppressed the overwhelming excimer fluorescence of pure **Tc** nanoparticles. Such excimer formation is known to be a trapping site for excited states, and the ability to suppress excimer formation is significant. Second, **NP**s composed of achiral ion pairs did not exhibit SF. The intermolecular interactions of **Tc** can be reduced by introducing a large counterion **TPMA**, as shown by a monomer‐like fluorescence with a high Φ_F_ of 43.5% and a long TCSPC lifetime. Compact, achiral **NMA** as counterion caused red shifts in absorption and fluorescence, reflecting strong interchromophore interactions. However, lowering the S_1_ energy level would have made SF more endothermic and unfavorable. Thus, using compact countercations alone does not provide sufficient driving force for SF progression.

Third, and most importantly, SF was observed for the chiral **Tc_R‐/S‐NEA_NP**s. The aqueous nanoparticles of **R‐/S‐NEA** showed the chiral orientation of **Tc** chromophores in nanosheets, as indicated by the prominent induced CD spectra. The absorption and fluorescence spectra of **Tc_R‐/S‐NEA_NP**s featured Φ_F_ and TCSPC lifetimes heavily quenched to ≈7% and 450 ps, respectively. The initially photoexcited hot (S_1_S_0_) relaxes within 8 ps into the (S_1_S_0_)_rel_, then populated ^1^(T_1_T_1_) after 160 ps with a TQY as high as 133% ±20%. Moreover, nsTAS measurements revealed subsequent decoherence of ^1^(T_1_T_1_) into (T_1_+T_1_) with an efficiency of ≈30% ±10%. Thus, the chiral self‐assembly of **Tc** chromophores promotes SF and provides a pathway for separating free triplets. It was also found that the size and shape of chiral counterions affect the molecular orientation and the resultant optical relaxation processes. When chiral but bulky **CyHx** was used as a counterion, regularity of the **Tc** chromophore alignment was reduced, forming defect sites that serve as singlet quenching sites, and SF was not observed.

Fourth, homochirality is essential to give two separated triplet excitons. SF also occurs for racemic **Tc_Rac_NP**s, but the populated ^1^(T_1_T_1_) species showed TTA back to the singlet state without decoherence into free triplets. These results reveal that chiral and long‐range ordered molecular alignments are vital in intermolecular SFs in the ion‐complexed aqueous **NP**s.

The present work is the first to demonstrate the utility of chirality as a critical molecular design that can simultaneously promote the population of ^1^(T_1_T_1_) and its separation into free triplets. Chiral SF‐exhibiting materials would also be designable using covalent approaches. We envisage that the concept of chiral molecular self‐assembly contributes widely to the development of functional triplet chemistry.

## Conflict of Interest

The authors declare no conflict of interest.

## Supporting information

Supporting Information

## Data Availability

The data that support the findings of this study are available in the supplementary material of this article.
