# Supplementary material for: Chirality in Singlet Fission: Controlling Singlet Fission in Aqueous Nanoparticles of Tetracenedicarboxylic Acid Ion Pairs
Source: Adv Sci (Weinh). 2024 Aug 13;11(39):2405864. doi: 10.1002/advs.202405864 (PMC11633336; doi:10.1002/advs.202405864)
Supplement: Supplementary file 1 — Supporting Information [file ADVS-11-2405864-s001.docx]

**Supporting information**

**Chirality in Singlet Fission: Controlling Singlet Fission in Aqueous Nanoparticles of Tetracenedicarboxylic Acid Ion Pairs**

Ilias Papadopoulos,^[a]^ Joseph Ka-Ho Hui,^[a]^ Masa-aki Morikawa,^[a,b]^ Yasuhito Kawahara,^[c]^ Kenji Kaneko,^[c]^ Kiyoshi Miyata,^[d]^ Ken Onda^[d]^ and Nobuo Kimizuka^[a,b]^*

^[a]^ Department of Applied Chemistry, Graduate School of Engineering, Kyushu University, 744 Motooka, Nishi-ku, Fukuoka 819 0395, Japan.

^[b]^ Center for Molecular Systems (CMS) Kyushu University, 744 Moto-oka, Nishi-ku, Fukuoka 819 0395, Japan.

^[c]^ Department of Materials Science and Engineering, Graduate School of Engineering, Kyushu University, 744 Motooka, Nishi-ku, Fukuoka 819 0395, Japan.

^[d]^ Department of Chemistry, Faculty of Science, Kyushu University, 744 Motooka, Nishi‑ku, Fukuoka 819 0395, Japan.

**Table of Contents**

General Experimental Methods S2–S3

Photophysical Characterization S4–S58

Triplet Quantum Yield Determination S59–S63

**General Experimental Methods**

**Materials.** All reagents and solvents were used as received unless otherwise noted. 5,12-diphenyltetracene-based dicarboxylic acid (**Tc**) was synthesized and purified by NARD Institute, Ltd. Stabilizer-free tetrahydrofuran (THF) from FUJIFILM Wako Chemical Corporation was used for spectroscopic measurements.

**Sample preparation.**

Aqueous nanoparticles of **Tc**^2-^ ion-paired with **R**-/**S**-**NEA** (**Tc**_**R**-/**S**-**NEA**_**NP**s), **CyHx** (**Tc**_**CyHx**_**NP**s), **TPMA** (**Tc**_**TPMA**_**NP**s), **NMA** (**Tc**_**NMA**_**NP**s) and a racemate mixture of **R**-/**S**-**NEA** (**Tc**_**Rac**_**NP**s) are obtained by the following procedure. First, THF solutions of 3 mM Tc and 6 mM of the respective amines were prepared (molar ratio 1:2), followed by solvent evaporation under room temperature. After leaving the resulting salts to dry under a vacuum for one day, they were re-dissolved in 1 mM argon-degassed THF solutions, which were injected into 9 ml of rapidly stirring deionized water in an argon atmosphere at room temperature. The mixtures were stirred again for 4 h in the dark to give opaque aqueous dispersions. Exclusive **Tc** (**Tc**_**NP**s) without counterions was employed as a reference, prepared by injecting an argon-degassed 0.2 ml THF solution of Tc into 9.8 ml of rapidly stirring deionized water under an argon atmosphere. Stirring was continued at room temperature for 4 h in the dark, giving an opaque aqueous dispersion of the pure Tc nanoparticles.

**Characterization.** UV-vis absorption spectra were recorded on a JASCO V-670 spectrophotometer. Luminescence spectra were measured by using a PerkinElmer LS 55 fluorescence spectrometer. The samples were excited with an incidence angle of 45° to the quartz cell surface, and the fluorescence was detected along the normal. Absolute quantum yields were calculated using a Hamamatsu C9920-02G instrument. Time‑resolved fluorescence lifetime measurements were carried out using a time‑correlated single photon counting lifetime spectroscopy (TCSPC) system, HAMAMATSU Quantaurus-Tau C11367-02. The quality of the fit has been judged by the fitting parameters such as χ^2^ (<1.2) and the visual inspection of the residuals. FT‑IR spectra were recorded on a SHIMADZU IRTracer-100 with a Smiths DuraSamplIR II ATR device. The fsTAS and nsTAS experiments were conducted using custom-built pump-probe setups. A Ti:sapphire regenerative amplifier (Spectra-Physics, Spitfire Ace) served as the primary light source, emitting pulses with a duration of 120 fs, a repetition rate of 1 kHz, a pulse energy of 4 mJ/pulse, and a central wavelength of 800 nm. This amplifier was seeded by a Ti:sapphire femtosecond mode-locked oscillator (Spectra-Physics, Tsunami) generating pulses with similar parameters, except for a repetition rate of 80 MHz and a pulse energy of 10 nJ/pulse. In the fs-TAS setup, the amplifier output was split, with one branch directed through a $\alpha$-BBO crystal for second harmonic generation at 400 nm to create the pump pulse, while the other was focused on a 3 mm thick sapphire crystal to generate white light (450-750 nm) for the probe pulse. For ns-TAS, pulses from an optical parametric oscillator (EKSPLA, NT242, 410 nm, 3 ns) were utilized as the excitation pulse, produced through pumping by the third harmonic generation of a Nd:YAG laser. A magic angle (~54.7 deg.) was employed between the pump and probe polarizations. The probe pulse after the sample was dispersed by a polychromator (JASCO, CT-10, 300 grooves/500 nm) and detected by a multichannel detection system equipped with a CMOS sensor (UNISOKU, USP-PSMM-NP). Measurements were conducted in quartz glass cuvettes under an inert gas atmosphere.

The fsTAS and nsTAS raw data were evaluated using multiwavelength and global analysis using the GloTarAn package. Global analysis was performed on the TAS raw data sets using the proposed kinetic models in Figure 8. The analytic solution to the coupled differential equations that describe the kinetic model is convoluted with a Gaussian instrument response function. After the least-squares fitting has converged, the raw data matrix is deconvoluted using the specific solution to the kinetic model and parameters from the fit to obtain the evolution-associated spectra and their populations as a function of time.

High-resolution TEM (HR-TEM) images were acquired using JEM-ARM200CF (JEOL, Ltd) to characterize the crystalline nature of materials.

**Photophysical Characterization**


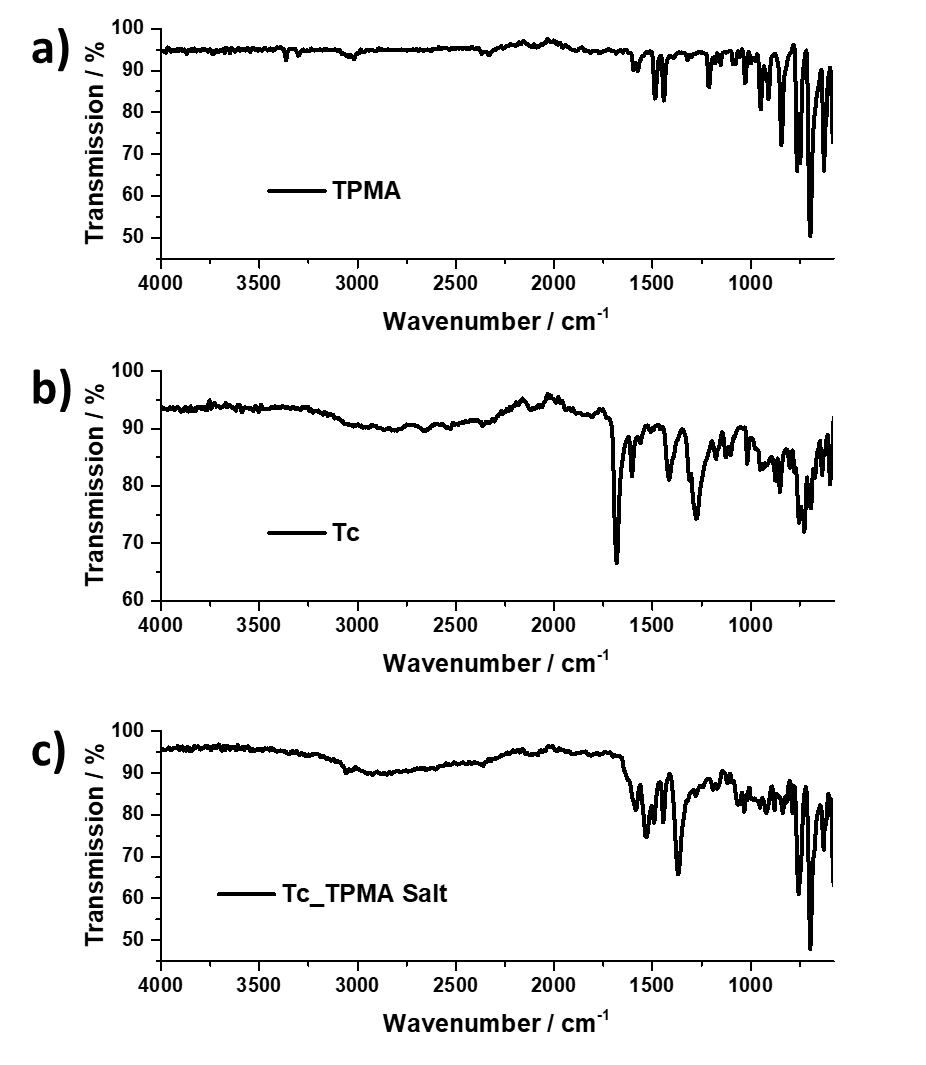


**Figure S1.** Comparison between the FT-IR spectra of a) **TPMA**, b) **Tc,** and c) **Tc_TPMA** salt.


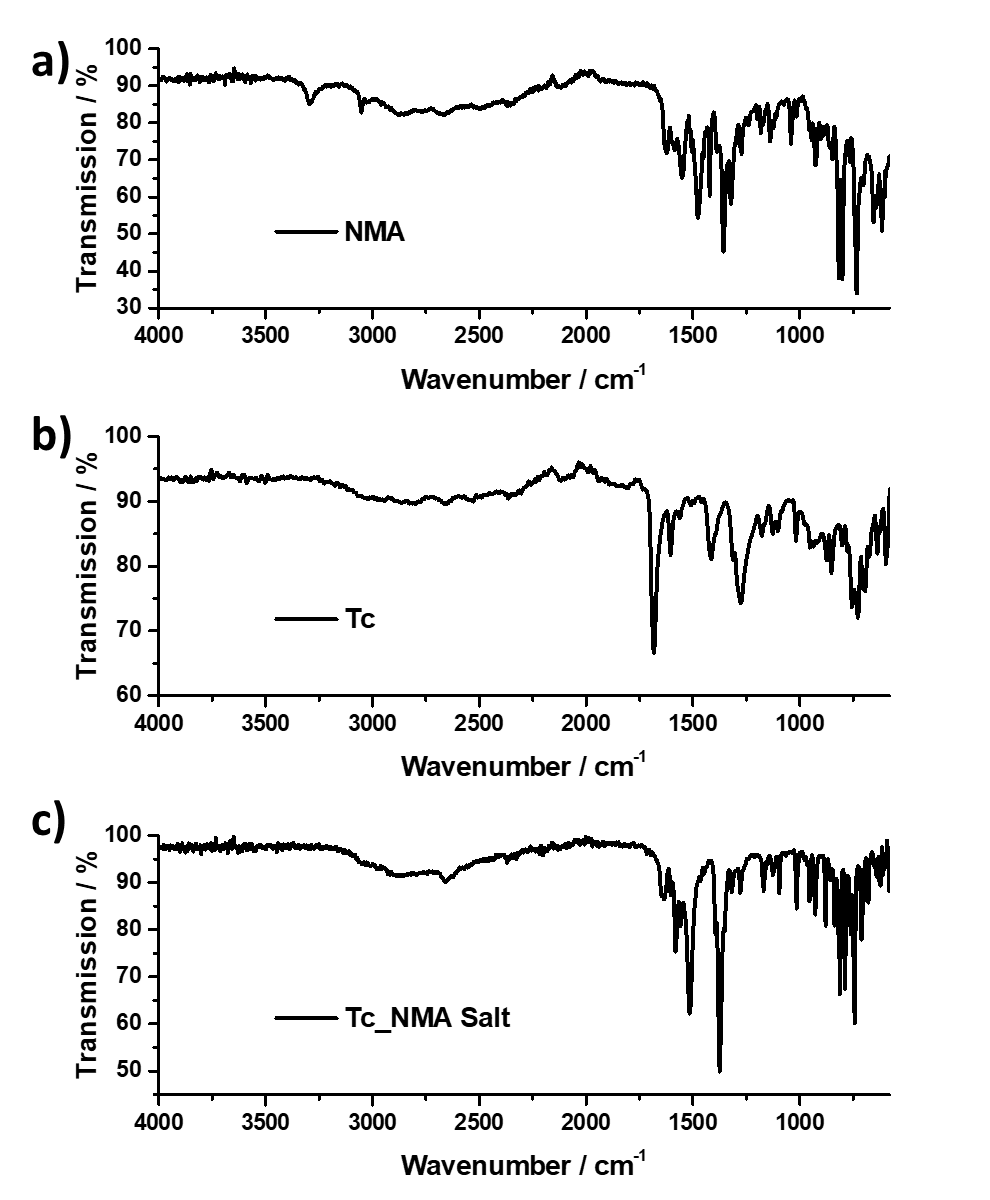


**Figure S2.** Comparison between the FT-IR spectra of a) **NMA**, b) **Tc,** and c) **Tc_NMA** salt.


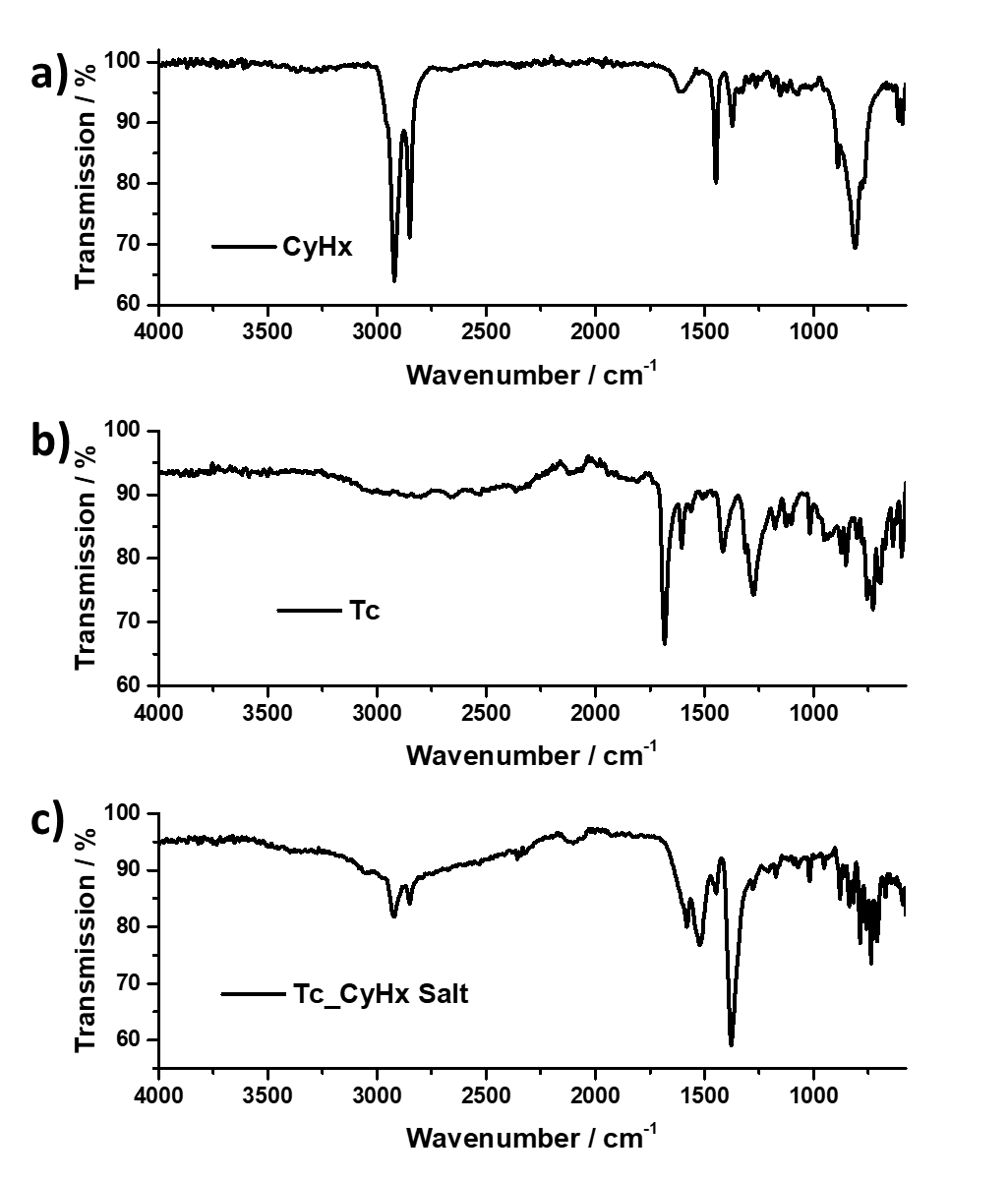


**Figure S3.** Comparison between the FT-IR spectra of a) **CyHx**, b) **Tc,** and c) **Tc_CyHx** salt.


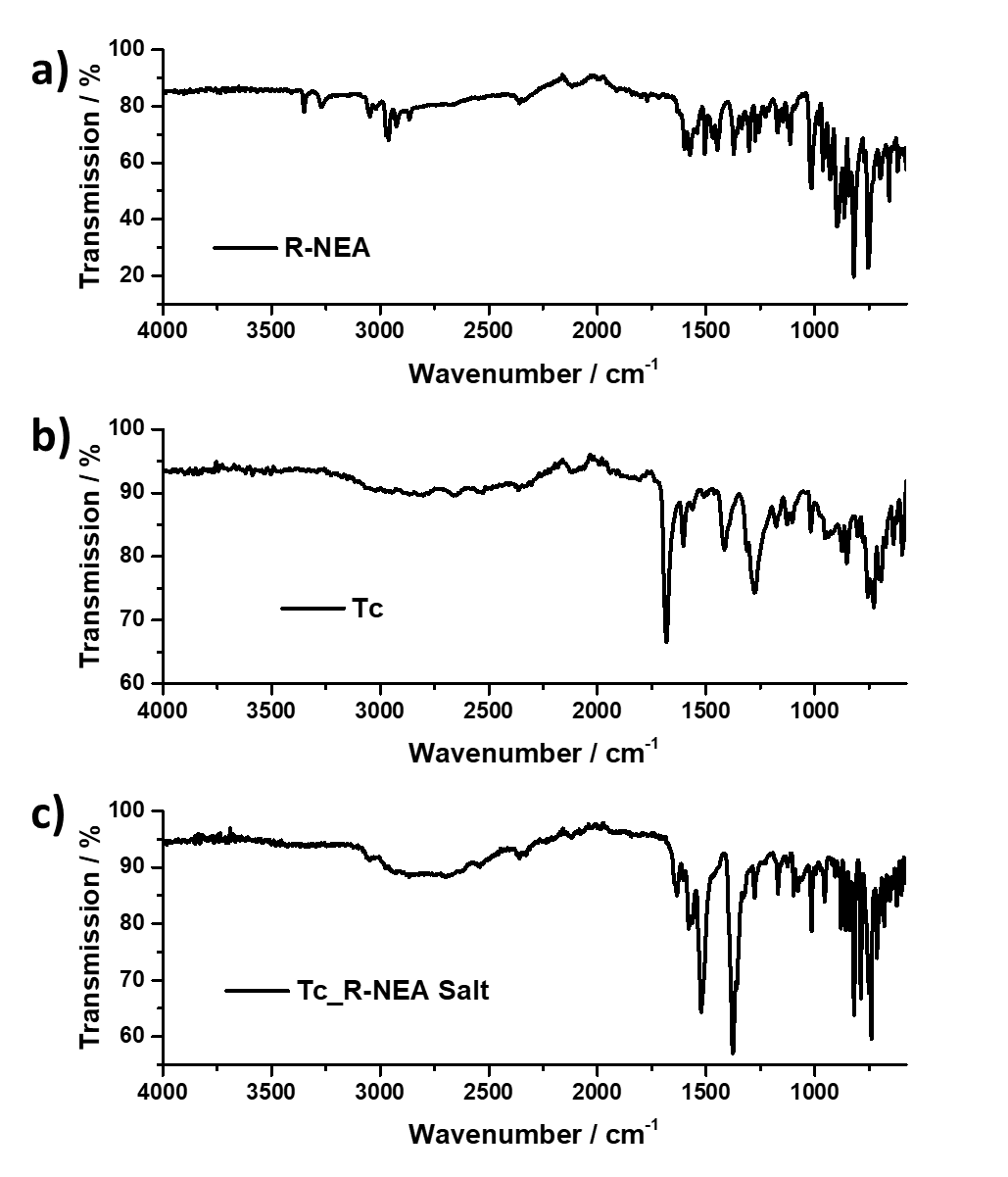


**Figure S4.** Comparison between the FT-IR spectra of a) **R-NEA**, b) **Tc,** and c) **Tc_R-NEA** salt.


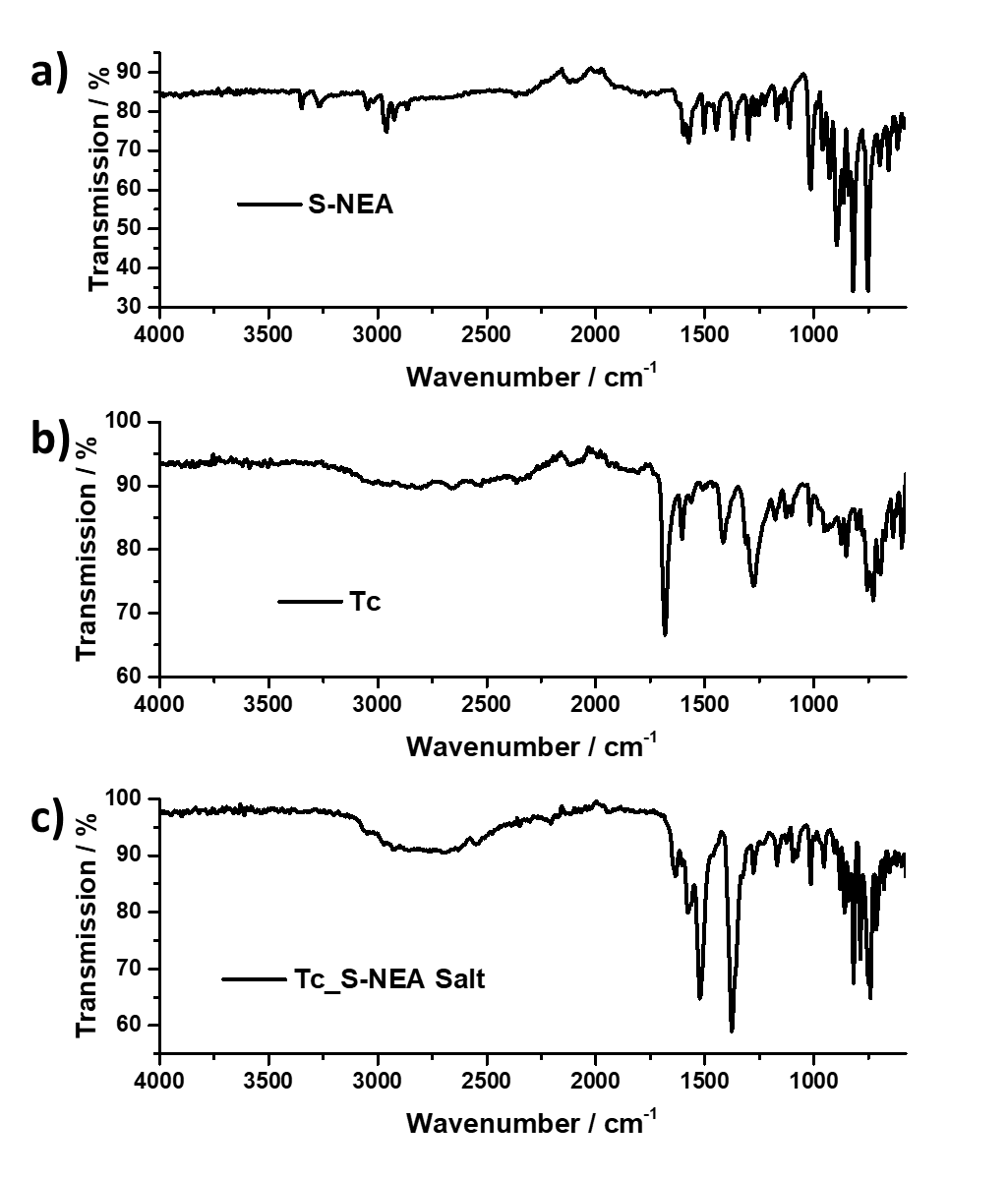


**Figure S5.** Comparison between the FT-IR spectra of a) **S-NEA**, b) **Tc,** and c) **Tc_S-NEA** salt.


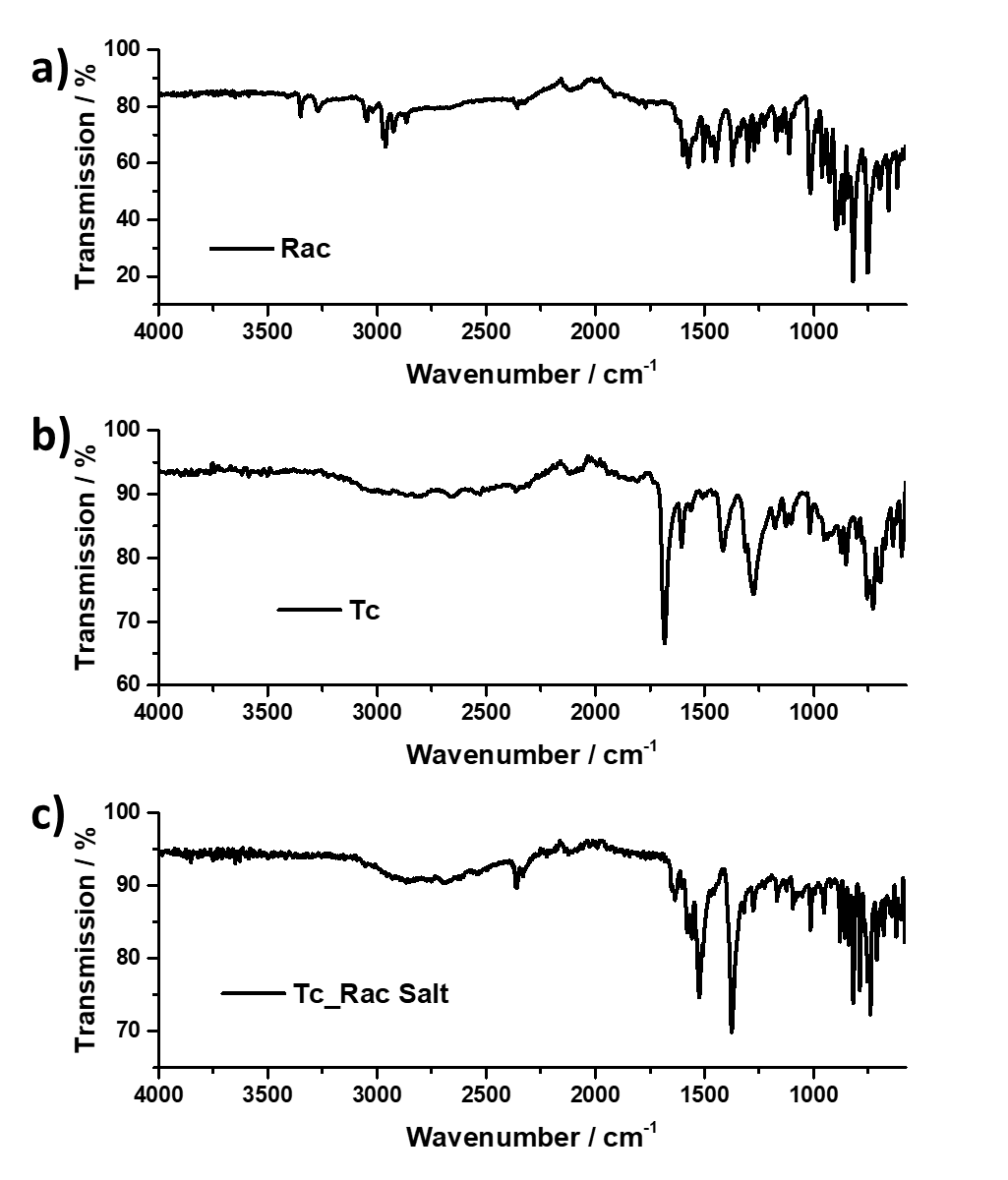


**Figure S6.** Comparison between the FT-IR spectra of a) **Rac**, b) **Tc,** and c) **Tc_Rac** salt.


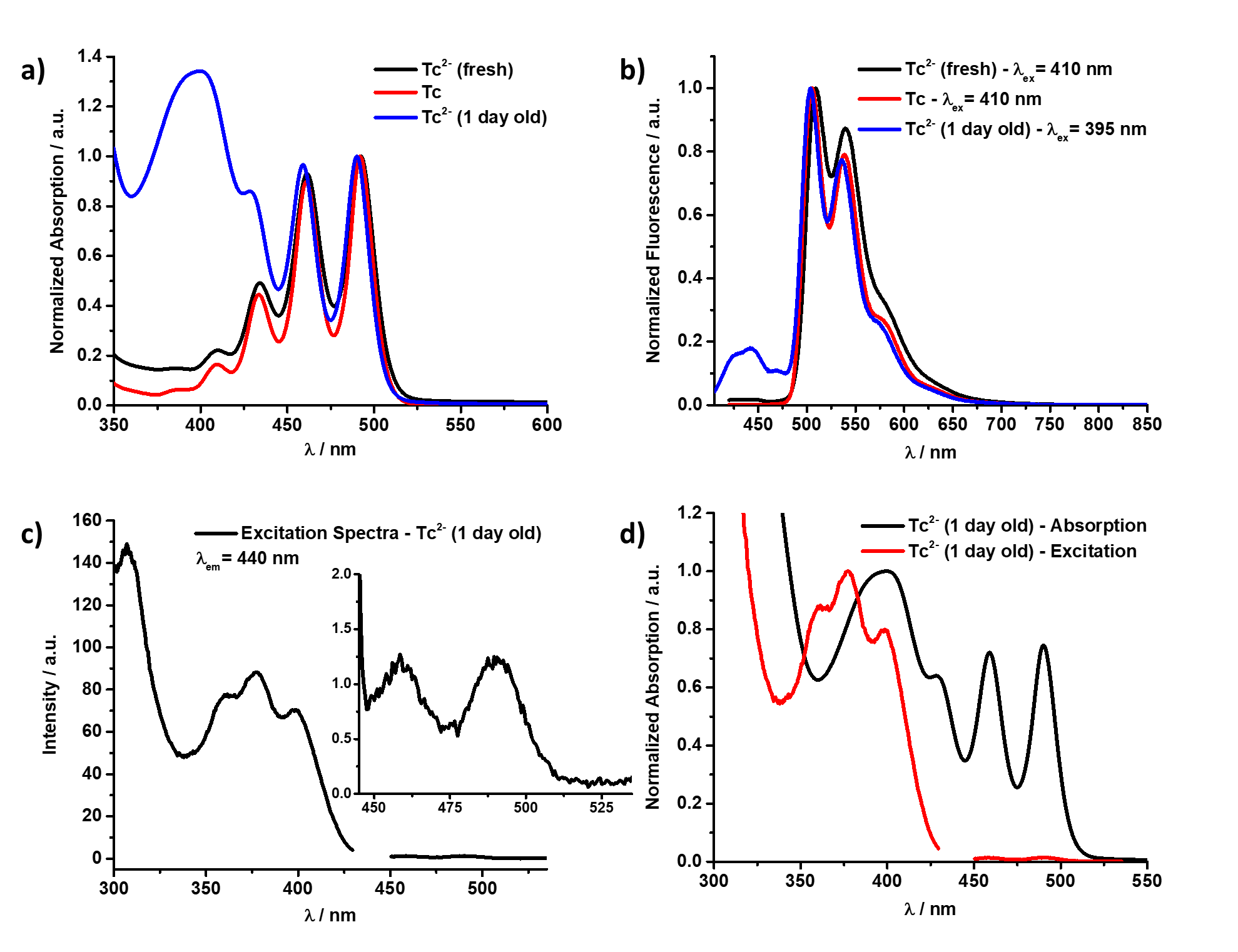


**Figure S7.** Comparison of a) absorption and b) fluorescence spectra between freshly deprotonated Tc (**Tc**^2-^, black), **Tc** (red), and 1 day-old solution of deprotonated Tc (**Tc**^2-^, blue), respectively. [**Tc**] = [**Tc**^2-^] = 0.1 mM in THF.


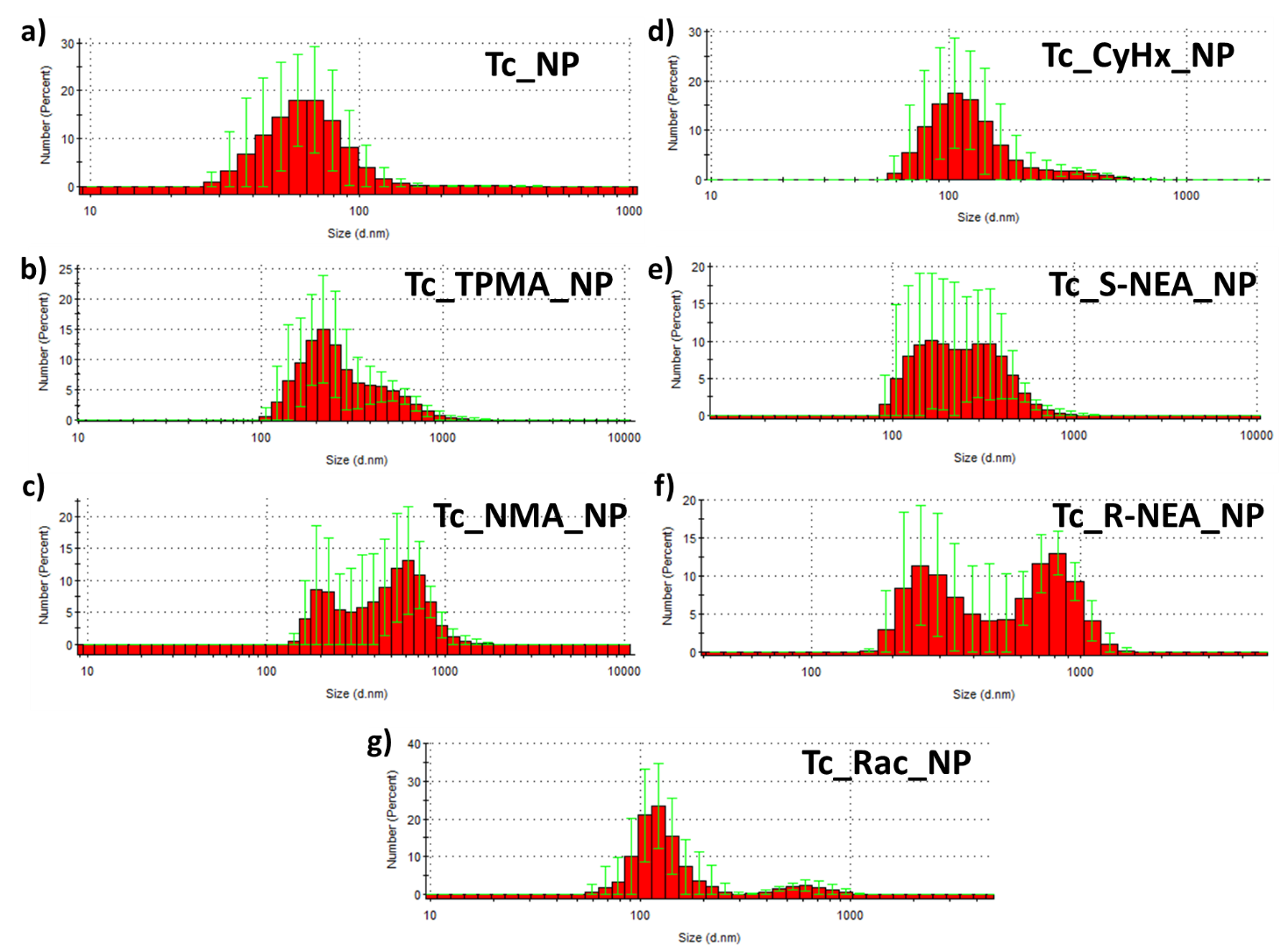


**Figure S8.** Dynamic light scattering (DLS) data showing the size distribution of a) **Tc_NP**, b) **Tc_TPMA_NP**, c) **Tc_NMA_NP**, d) **Tc_CyHx_NP,** e) **Tc_S-NEA_NP**, f) **Tc_R-NEA** and g) **Tc_Rac_NP**.


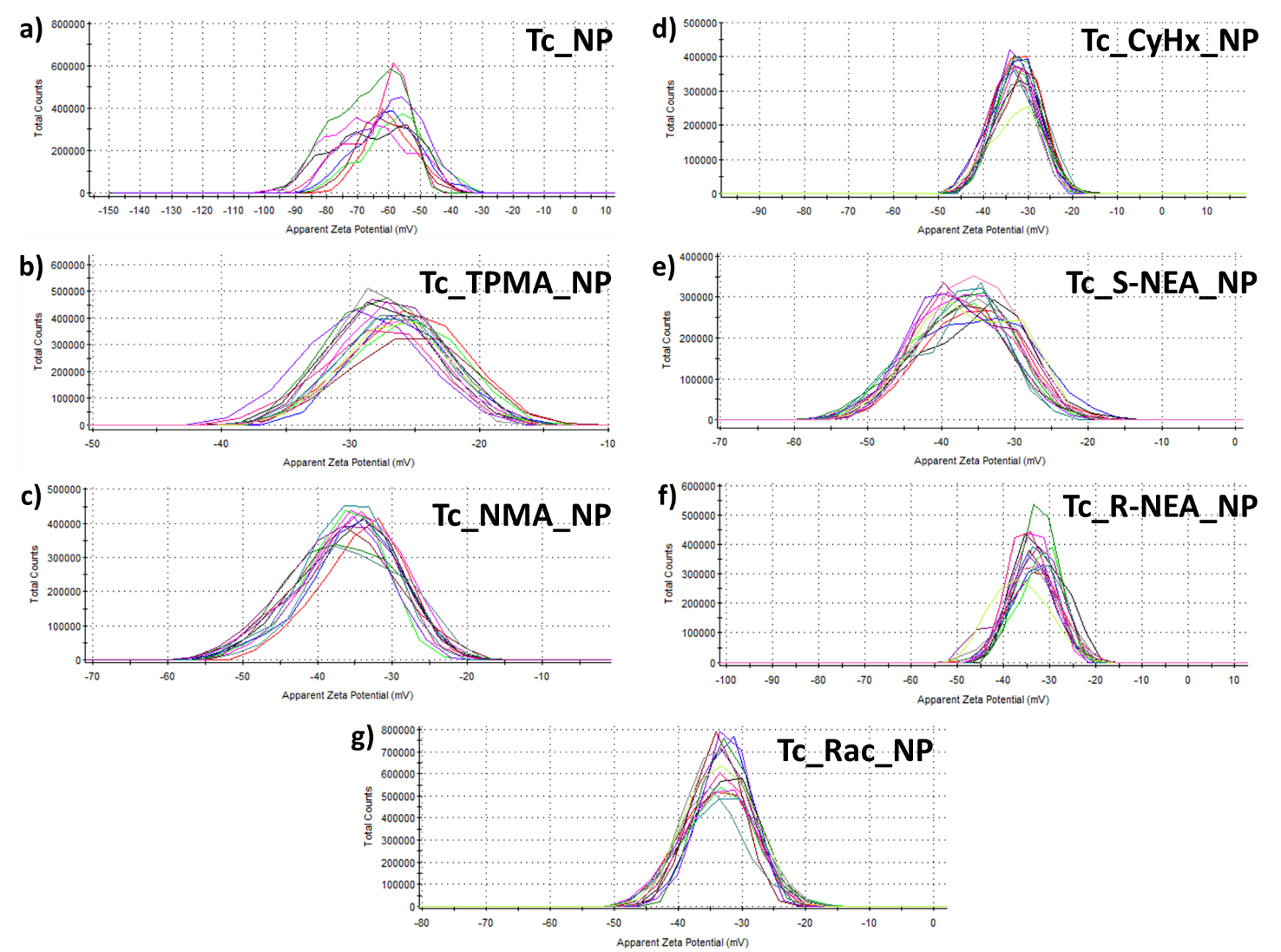


**(-35 mV)**

**(-60 mV)**

**(-33 mV)**

**(-33 mV)**

**(-34 mV)**

**(-34 mV)**

**(-26 mV)**

**Figure S9.** Zeta potential of a) **Tc_NP**, b) **Tc_TPMA_NP**, c) **Tc_NMA_NP**, d) **Tc_CyHx_NP,** e) **Tc_S-NEA_NP**, f) **Tc_R-NEA** and g) **Tc_Rac_NP**. Overlapping colored lines represent multiple measurement cycles.


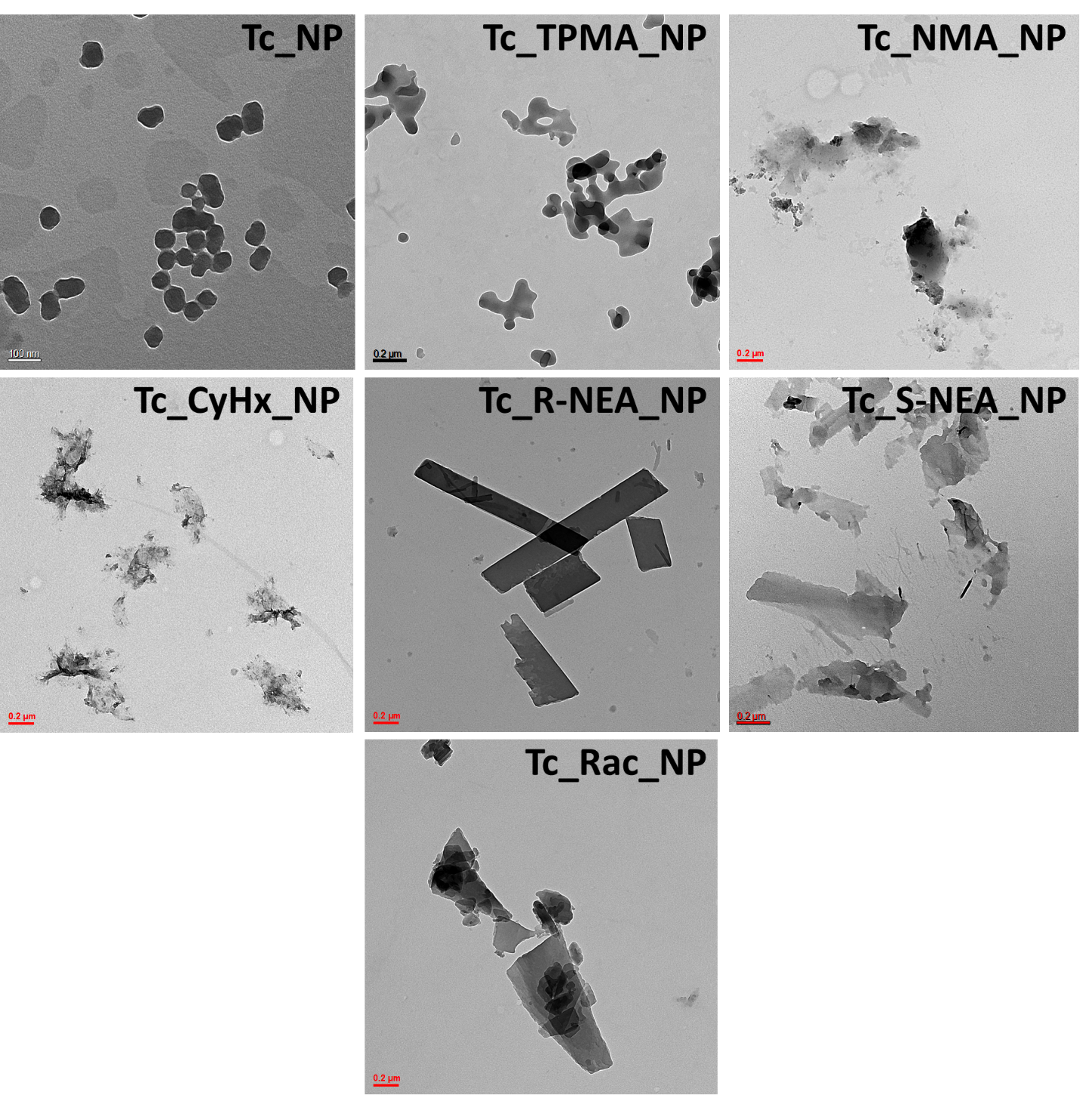


**(f)**

**(e)**

**(d)**

**(g)**

**(c)**

**(b)**

**(a)**

**Figure S10.** Transmission electron microscopy (TEM) images of (a) **Tc_NP**, (b) **Tc_TPMA_NP**, **(c) Tc_NMA_NP**, **(d) Tc_CyHx_NP**, (e) **Tc_R-NEA_NP**, **(f) Tc_S-NEA_NP** and (g) **Tc_Rac_NP**.


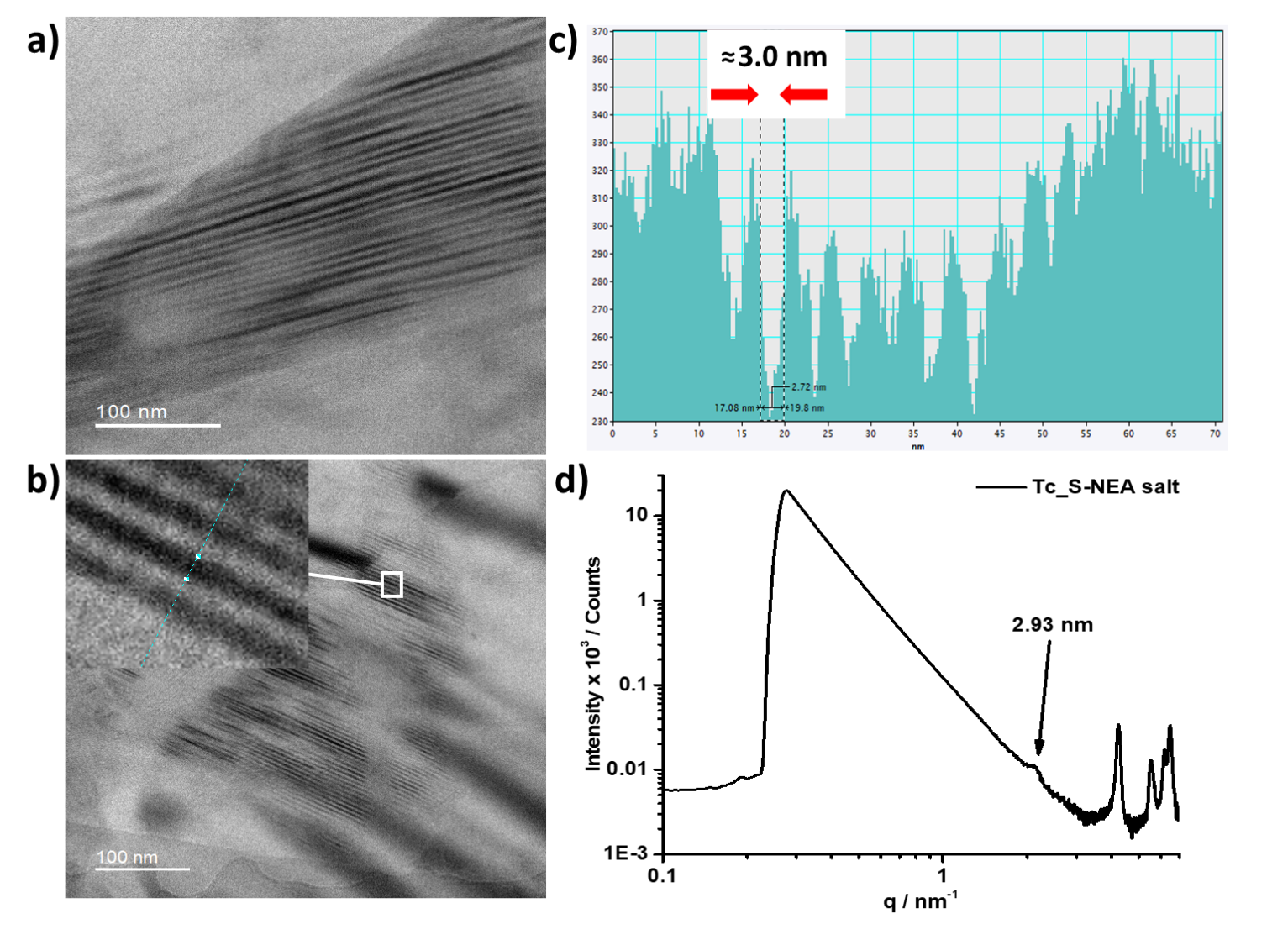


**Figure S11.** High-resolution transmission electron microscopy (HR-TEM) images of **Tc_S‑NEA_NP** (a, b), corresponding depth profile (c) of the inset shown in b) and grazing-incidence small‑angle X-ray scattering (GI-SAXS) analysis (d) corroborating the observed thickness in the depth profile of c).


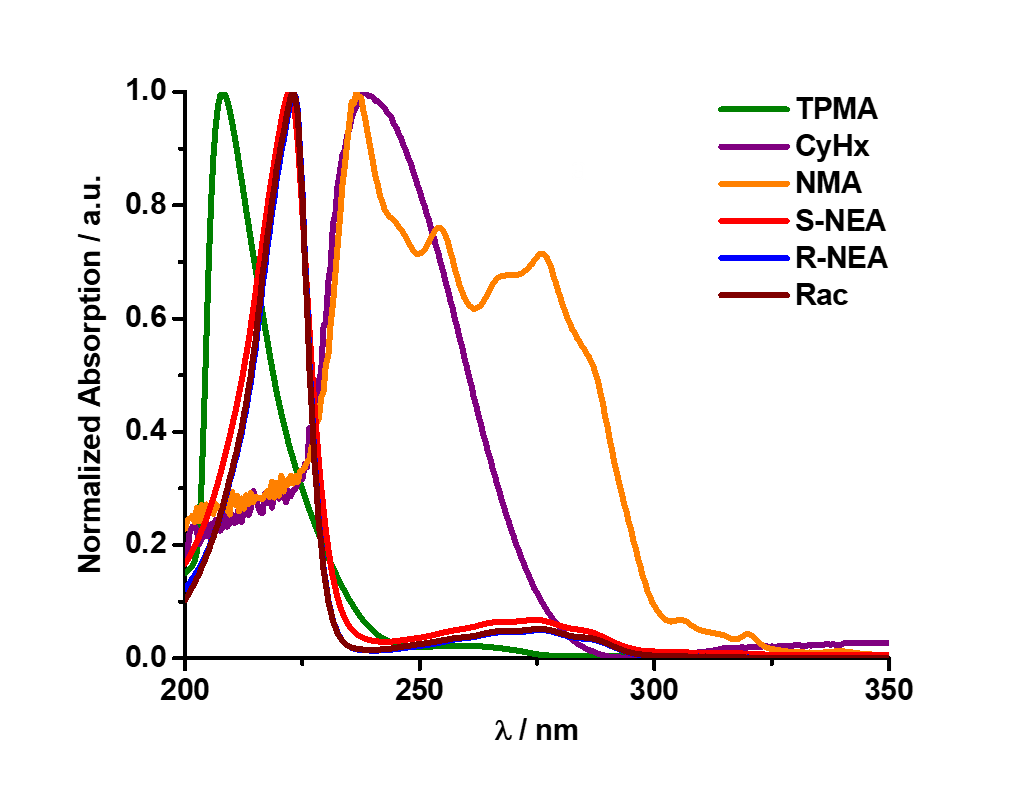


**Figure S12.** Normalized absorption spectra of **TPMA** (green), **CyHx** (purple), **NMA** (orange), **S-NEA** (red), **R-NEA** (blue) and **Rac** (brown) in THF, respectively.


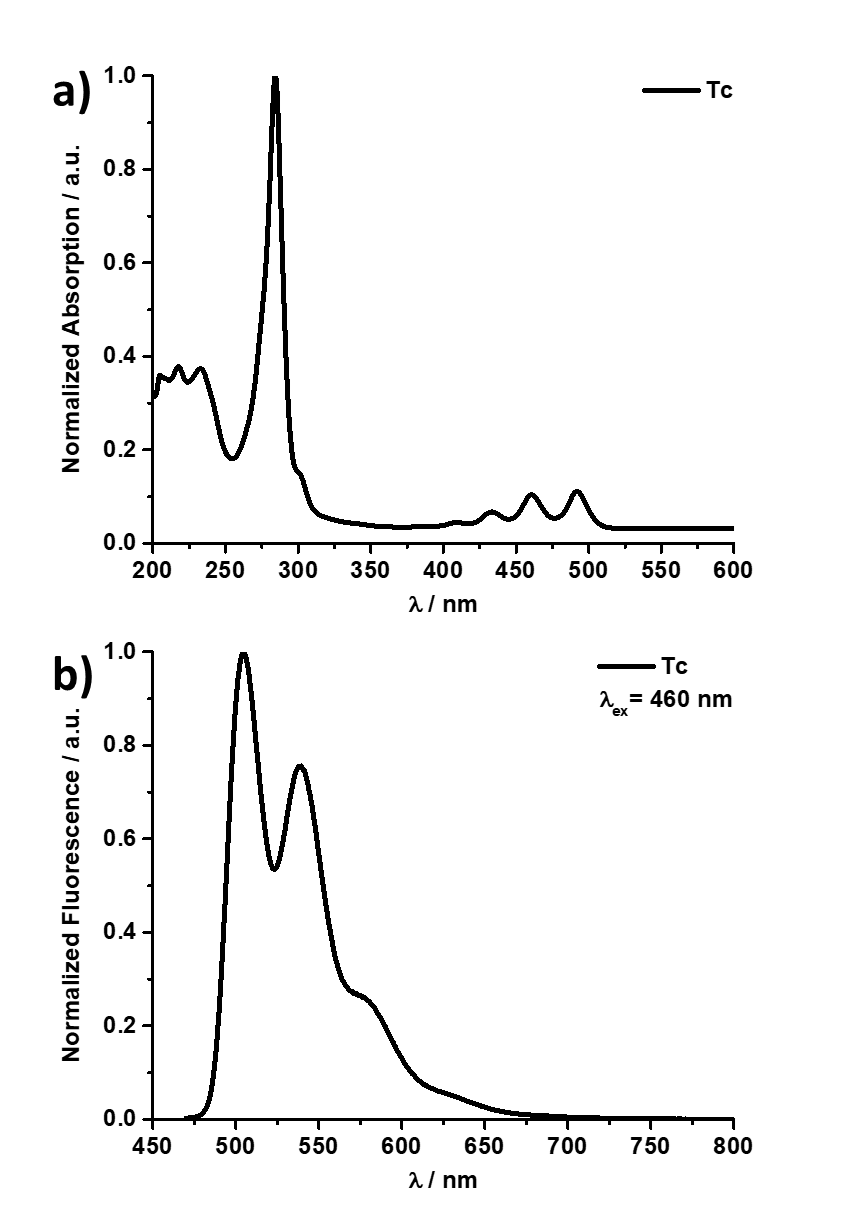


**Figure S13.** Normalized a) absorption and b) fluorescence spectra of **Tc** in THF.


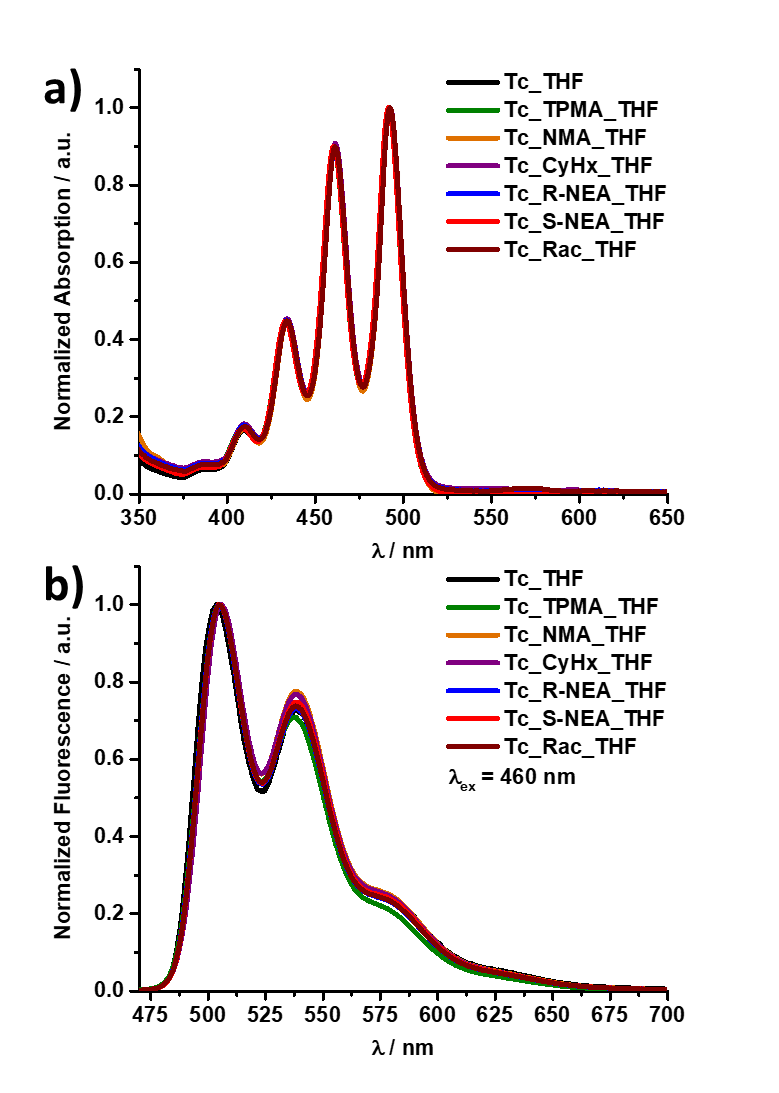


**Figure S14.** Comparison of the normalized a) absorption and b) fluorescence spectra of **Tc** (black), **Tc_TPMA** salt (green), **Tc_NMA** salt (orange), **Tc_CyHx** salt (purple), **Tc_R‑NEA** salt (blue), **Tc_S-NEA** salt (red) and **Tc_Rac** salt (brown) in THF, respectively.


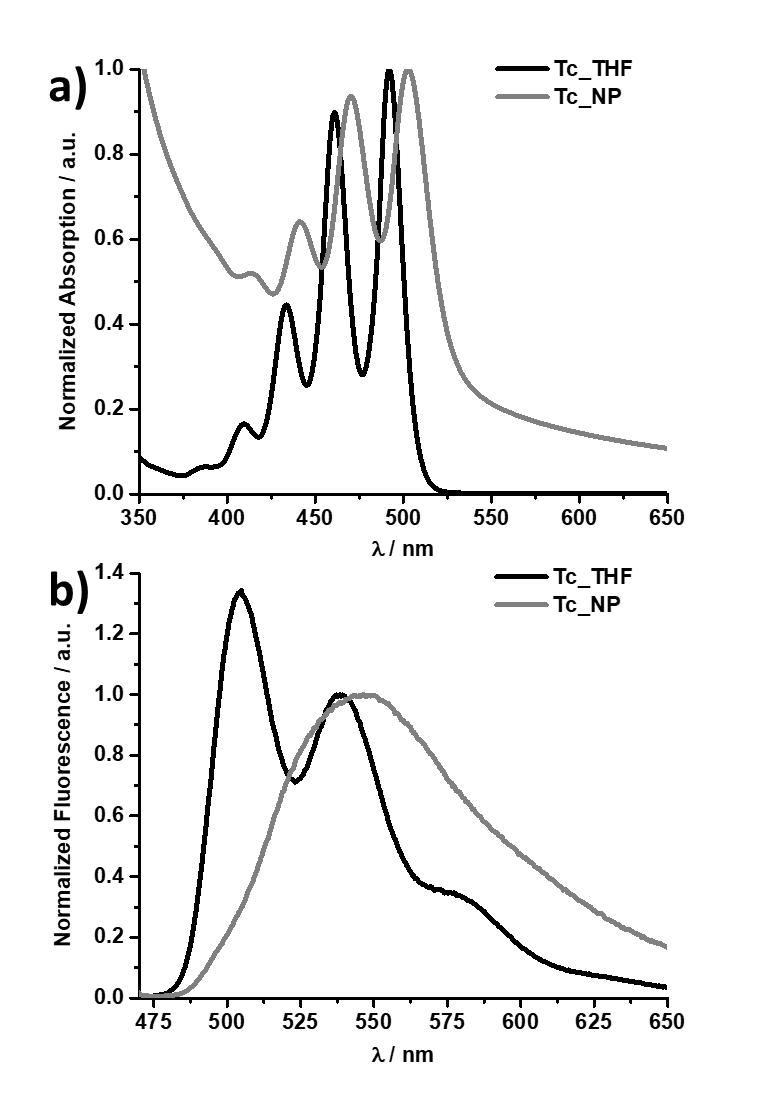


**Figure S15.** Normalized steady-state a) absorption and b) fluorescence spectra of **Tc** in THF (black) and **Tc_NP** in water (grey), respectively. The fluorescence spectra were recorded at an excitation wavelength of 460 nm.

**Table S1.** Listing of the steady-state absorption and fluorescence maxima and the fluorescence quantum yield of **Tc**, **Tc_TPMA** salt, **Tc_NMA** salt, **Tc_CyHx** salt, **Tc_R‑NEA** salt, **Tc_S-NEA** salt and **Tc_Rac** salt molecularly dissolved in THF, respectively.

| System / Solvent | λ_abs_ / nm | λ_em_ / nm | Φ_F_ / % |
| --- | --- | --- | --- |
| Tc / THF | 492 | 503.5 | 50.8 |
| Tc_TPMA / THF | 492.5 | 505 | 51.0 |
| Tc_NMA / THF | 492 | 505.5 | 51.2 |
| Tc_CyHx / THF | 492.5 | 506 | 53.4 |
| Tc_R-NEA / THF | 492.5 | 505 | 53.6 |
| Tc_S-NEA / THF | 491.5 | 504.5 | 54.0 |
| Tc_Rac / THF | 492.5 | 505 | 51.2 |

**Table S2.** Comparison of the steady-state absorption and fluorescence maxima and the fluorescence quantum yield of **Tc** in THF and **Tc_TPMA_NP**, **Tc_NMA_NP**, **Tc‑CyHx_NP**, **Tc_S‑NEA_NP**, **Tc_R-NEA_NP** and **Tc_Rac_NP** in water, respectively.

| System / Solvent | λ_abs_ / nm | λ_em_ / nm | Φ_F_ / % |
| --- | --- | --- | --- |
| Tc / THF | 492 | 505 | 50.8 |
| Tc_NPs / water | 502.5 | 542 | 2.7 |
| Tc_TPMA_NPs / water | 496.5 | 510 | 43.5 |
| Tc_ NMA_NPs / water | 508.5 | 515 | 8.4 |
| Tc_CyHx_NPs / water | 493.5 | 510 | 10.1 |
| Tc_S-NEA_NPs / water | 498.5 | 507 | 6.4 |
| Tc_R-NEA_NPs / water | 503 | 509 | 7.9 |
| Tc_Rac_NPs / water | 503 | 508 | 2.0 |

**Table S3.** Listing of the fluorescence lifetimes (τ) and their respective relative amplitudes (Rel. A) from time‑correlated single photon counting measurements of **Tc** in THF, **Tc_TPMA_NP**, **Tc_NMA_NP**, **Tc_CyHx_NP**, **Tc_R‑NEA_NP**, **Tc_S-NEA_NP** and **Tc_Rac_NP**, respectively.

| System / Solvent | τ_1_ / ns  (Rel. A_1_ / %) | τ_2_ / ns  **(Rel. A_2_ / %)** |
| --- | --- | --- |
| Tc / THF | 9.61 (100) | - |
| Tc_TPMA_NPs / water | 9.33 (100) | - |
| Tc_ NMA_NPs / water | 10.35 (100) | - |
| Tc_CyHx_NPs / water | 0.32 (99.10) | 9.17 (0.90) |
| Tc_R-NEA_NPs / water | 0.46 (98.13) | 10.50 (1.87) |
| Tc_S-NEA_NPs / water | 0.44 (98.87) | 14.48 (1.13) |
| Tc_Rac_NPs / water | 0.99 (92.78) | 12.99 (7.22) |


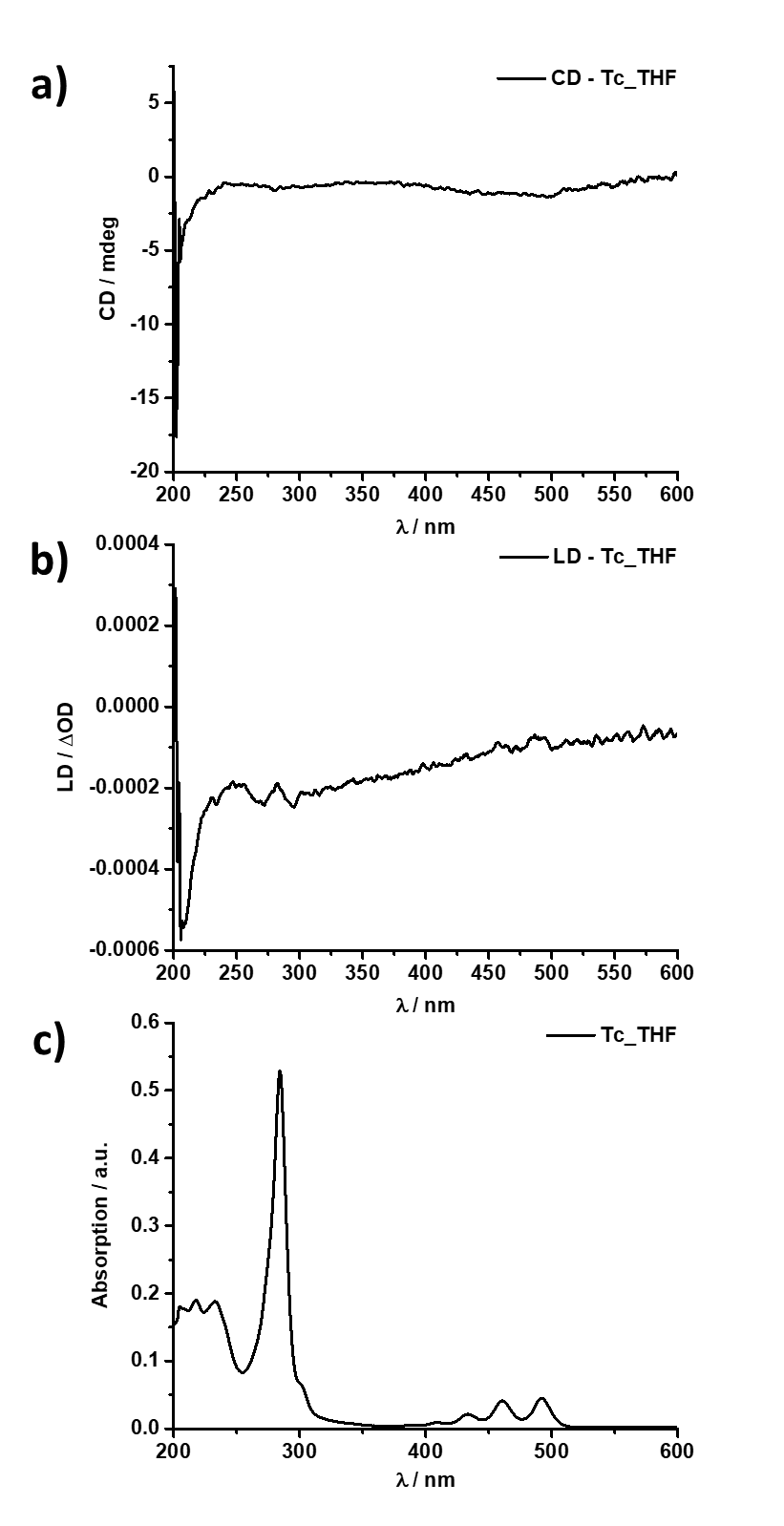


**Figure S16.** a) Circular dichroism (CD), b) linear dichroism spectra (LD), and c) corresponding absorption spectra of **Tc** in THF.


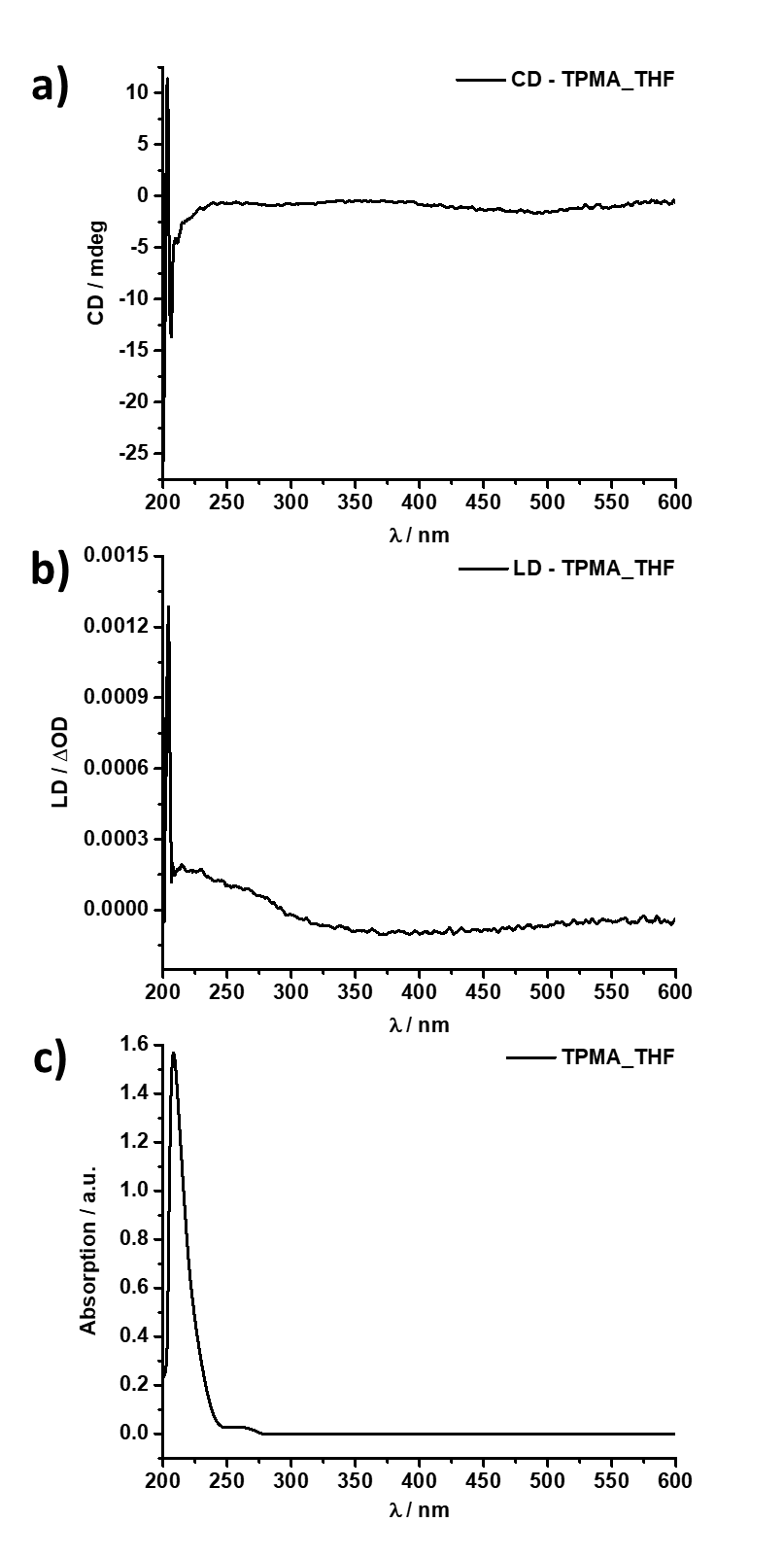


**Figure S17.** a) Circular dichroism (CD), b) linear dichroism spectra (LD) and c) corresponding absorption spectra of **TPMA** in THF.


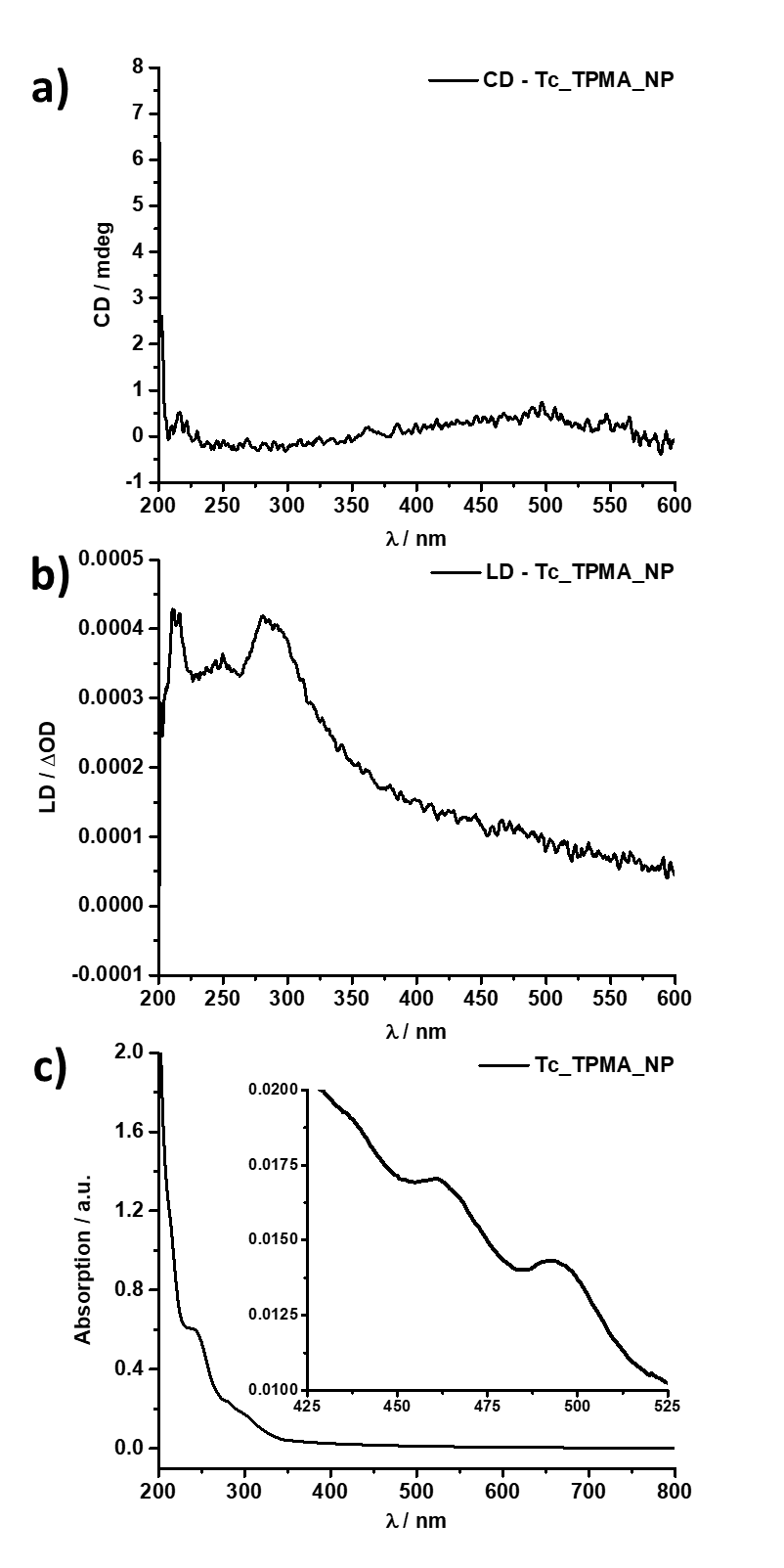


**Figure S18.** a) Circular dichroism (CD), b) linear dichroism spectra (LD) and c) corresponding absorption spectra of aqueous **Tc_TPMA** NPs.


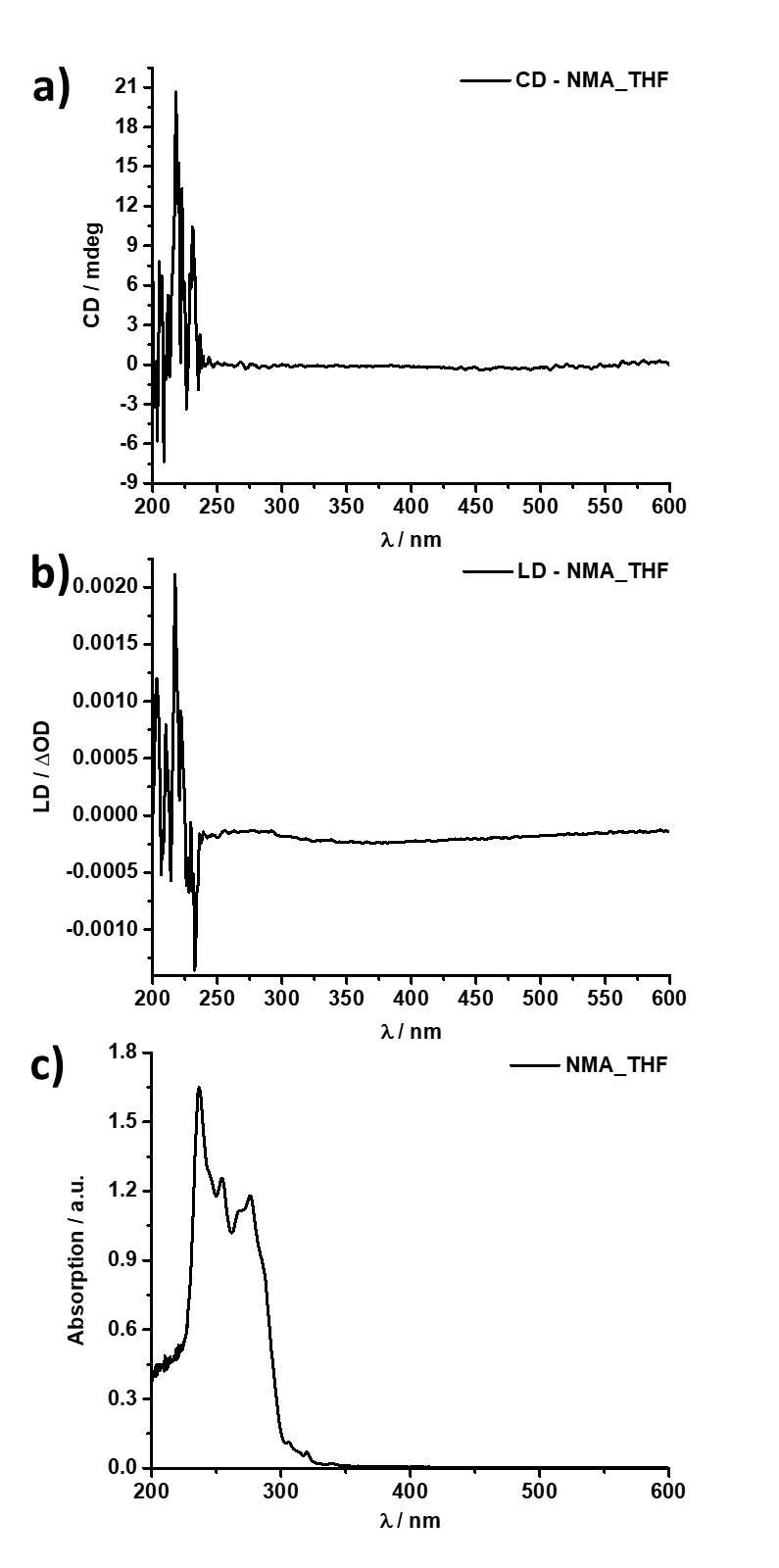


**Figure S19.** a) Circular dichroism (CD), b) linear dichroism spectra (LD) and c) corresponding absorption spectra of **NMA** in THF.


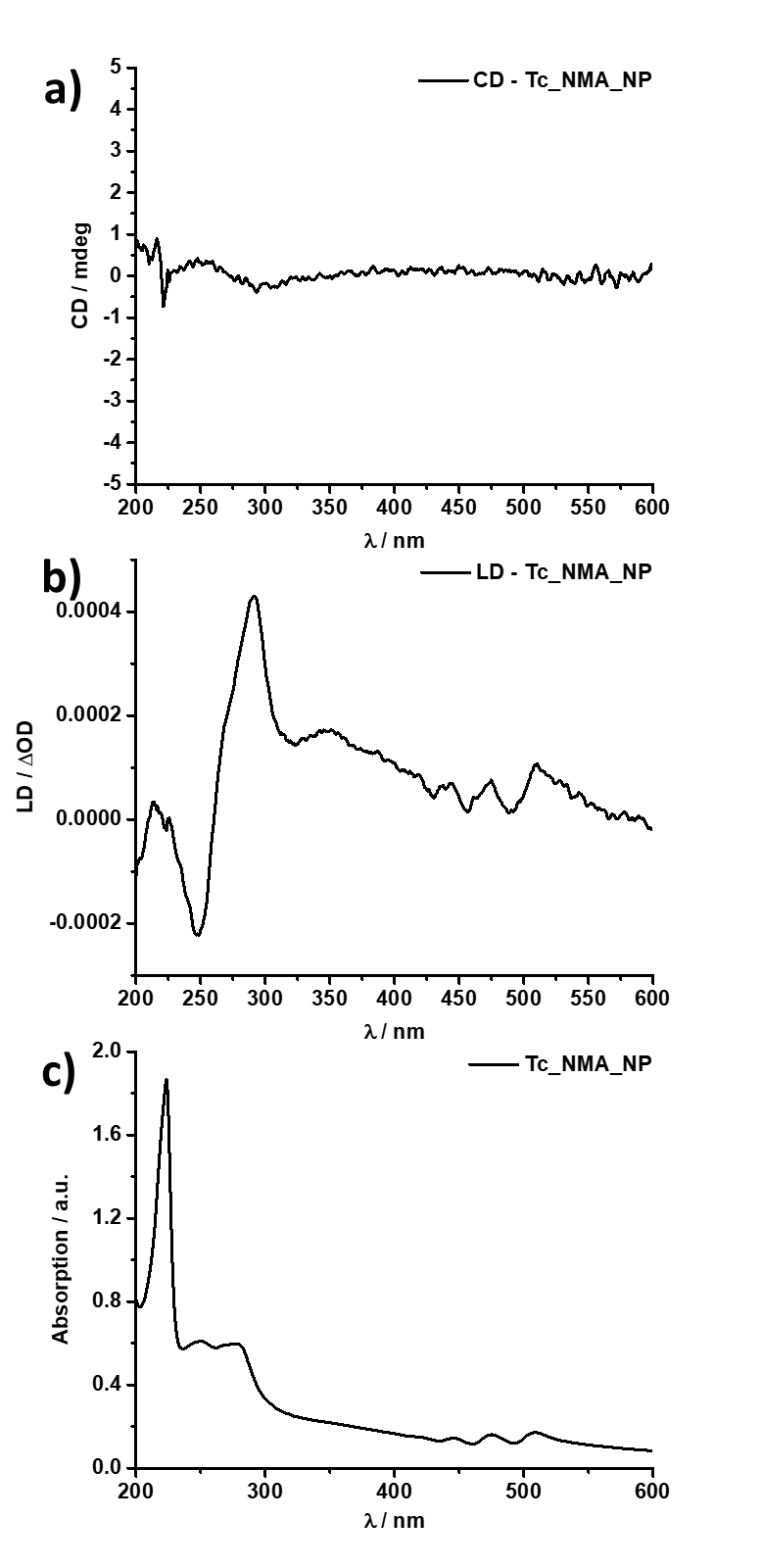


**Figure S20.** a) Circular dichroism (CD), b) linear dichroism spectra (LD) and c) corresponding absorption spectra of aqueous **Tc_NMA** NPs.


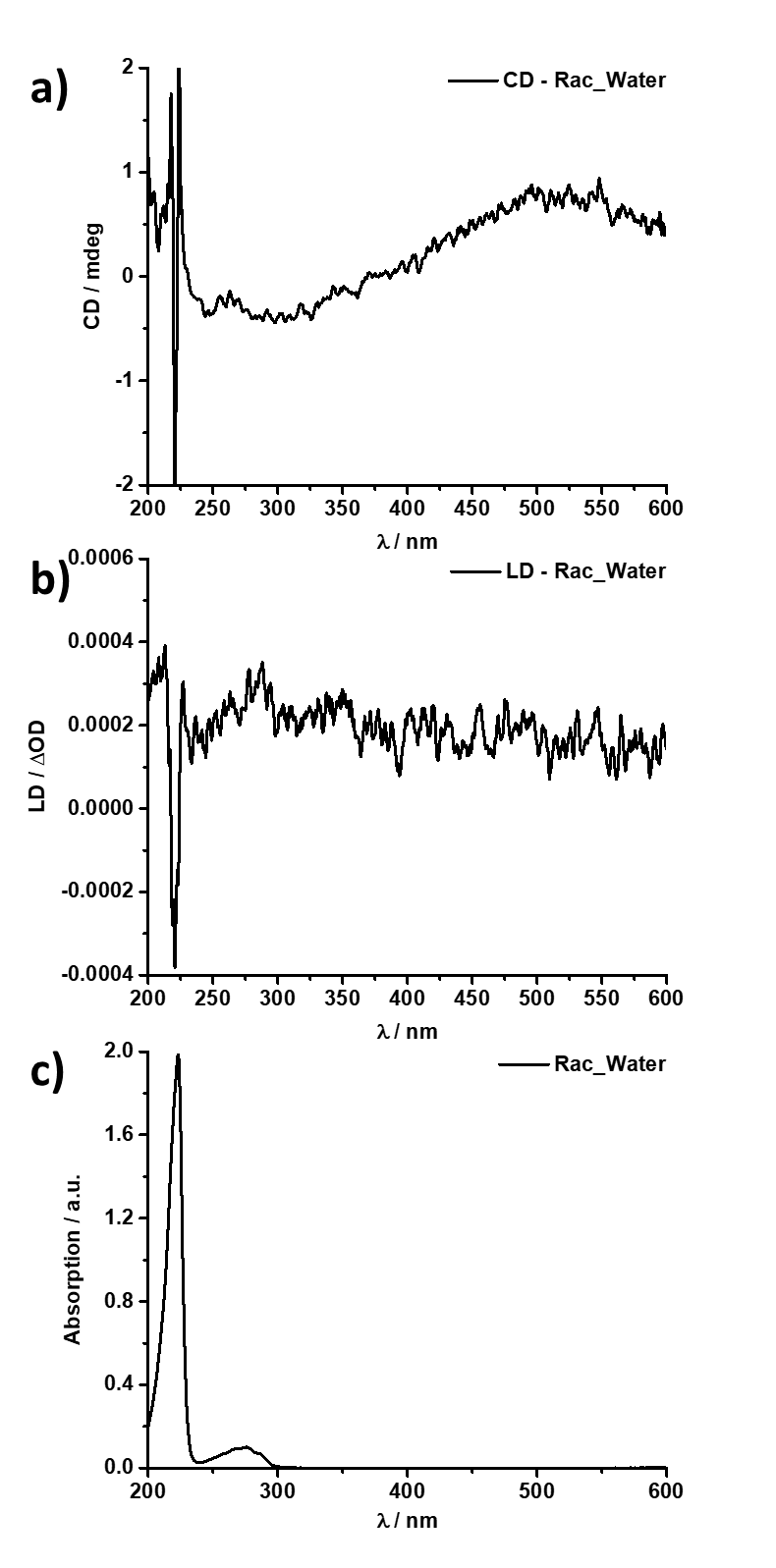


**Figure S21.** a) Circular dichroism (CD), b) linear dichroism spectra (LD) and c) corresponding absorption spectra of **Rac** in deionized water.


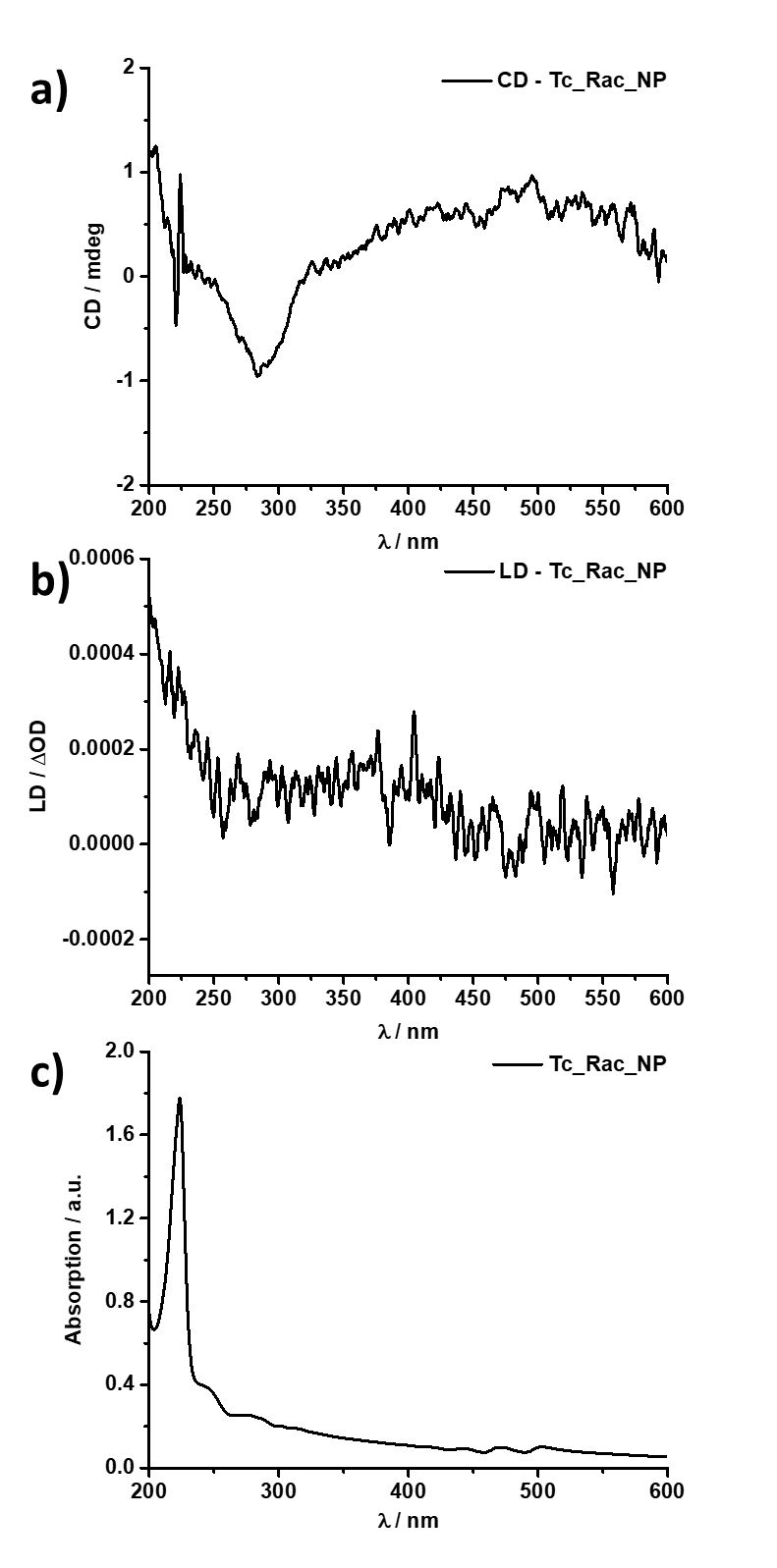


**Figure S22.** a) Circular dichroism (CD), b) linear dichroism spectra (LD) and c) corresponding absorption spectra of **Tc_Rac_NP**.


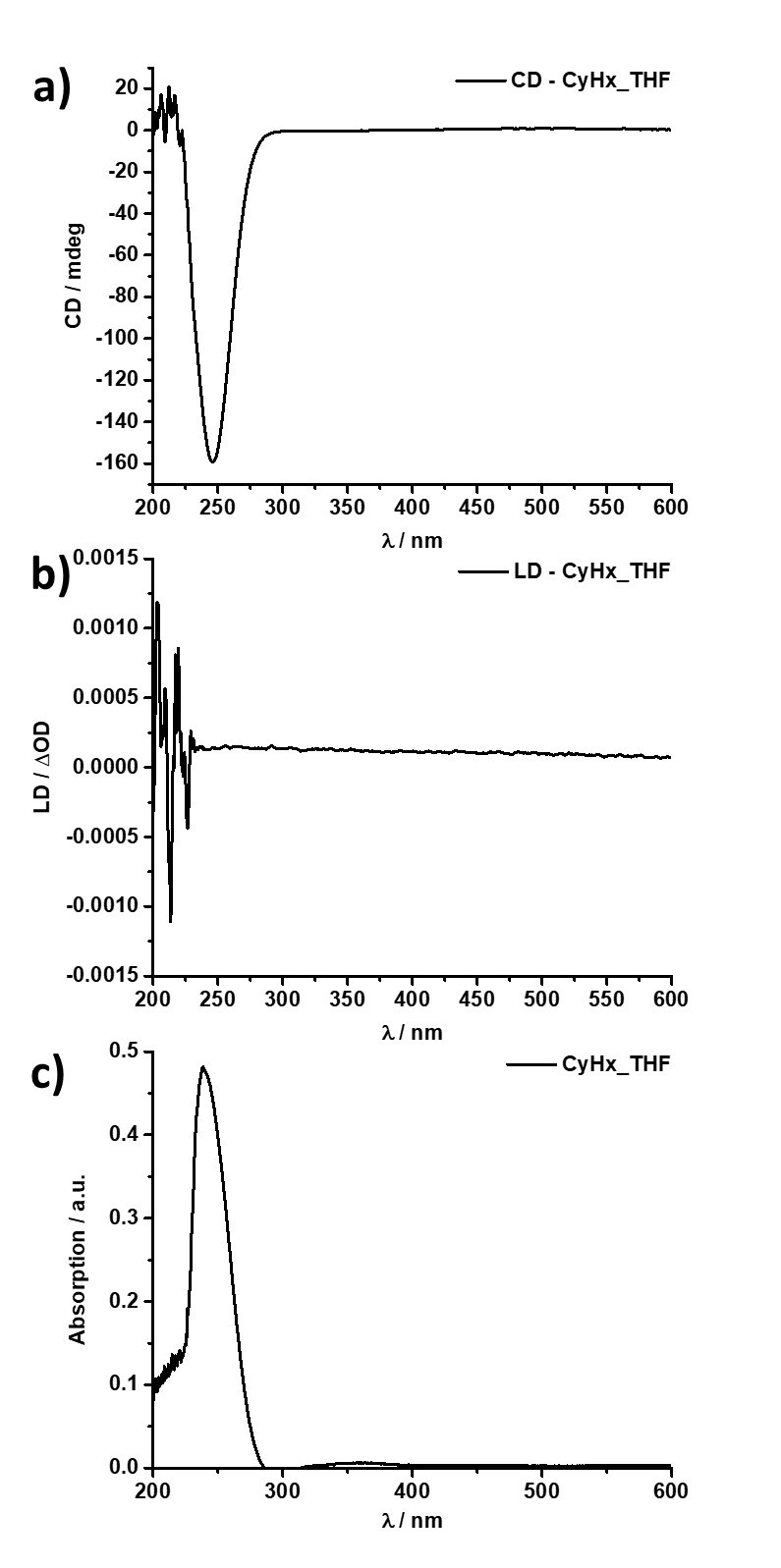


**Figure S23.** a) Circular dichroism (CD), b) linear dichroism spectra (LD) and c) corresponding absorption spectra of **CyHx** in THF.


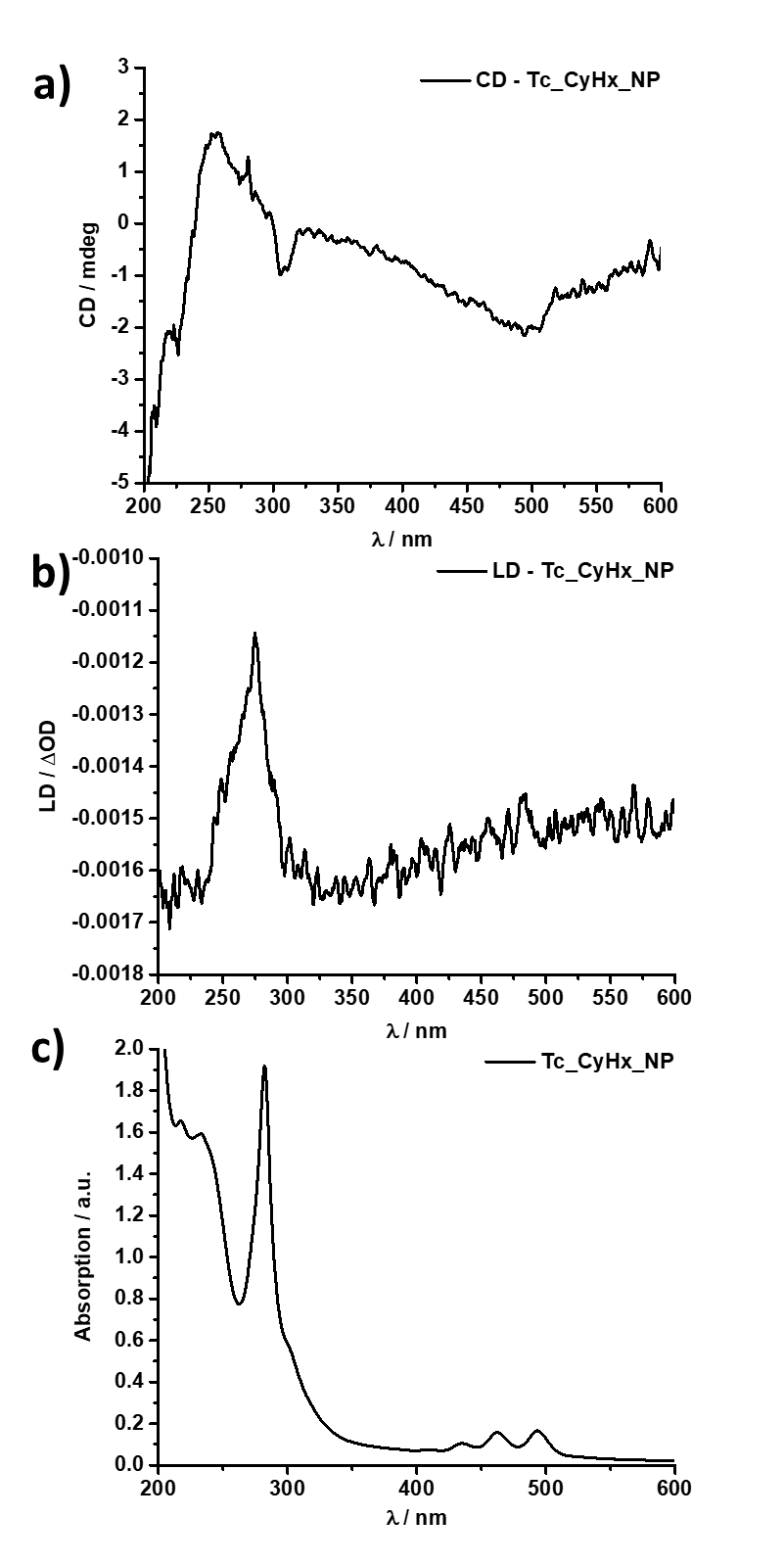


**Figure S24.** a) Circular dichroism (CD), b) linear dichroism spectra (LD) and c) corresponding absorption spectra of aqueous **Tc_CyHx** NPs.


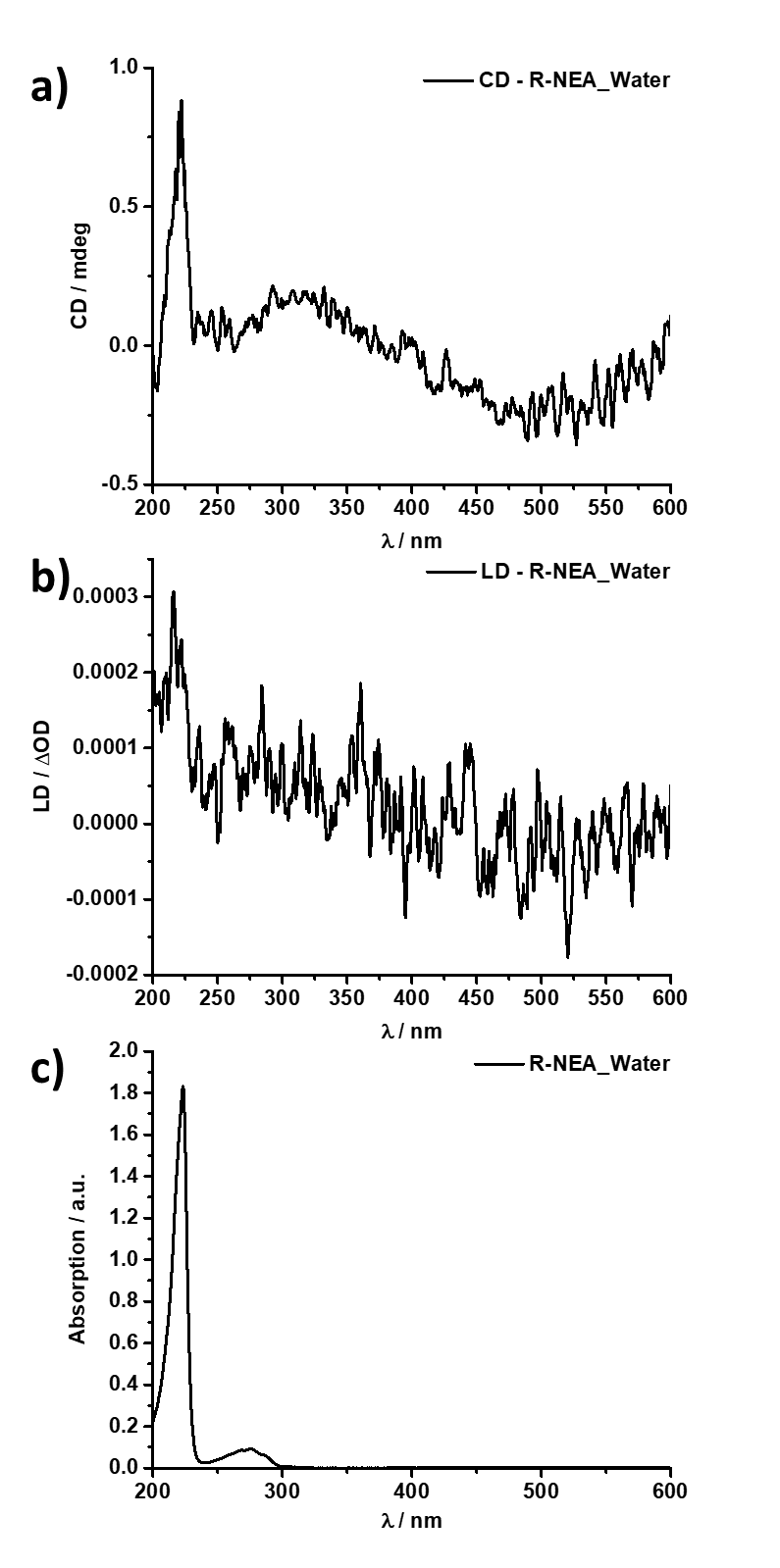


**Figure S25.** a) Circular dichroism (CD), b) linear dichroism spectra (LD) and c) corresponding absorption spectra of **R-NEA** in deionized water.


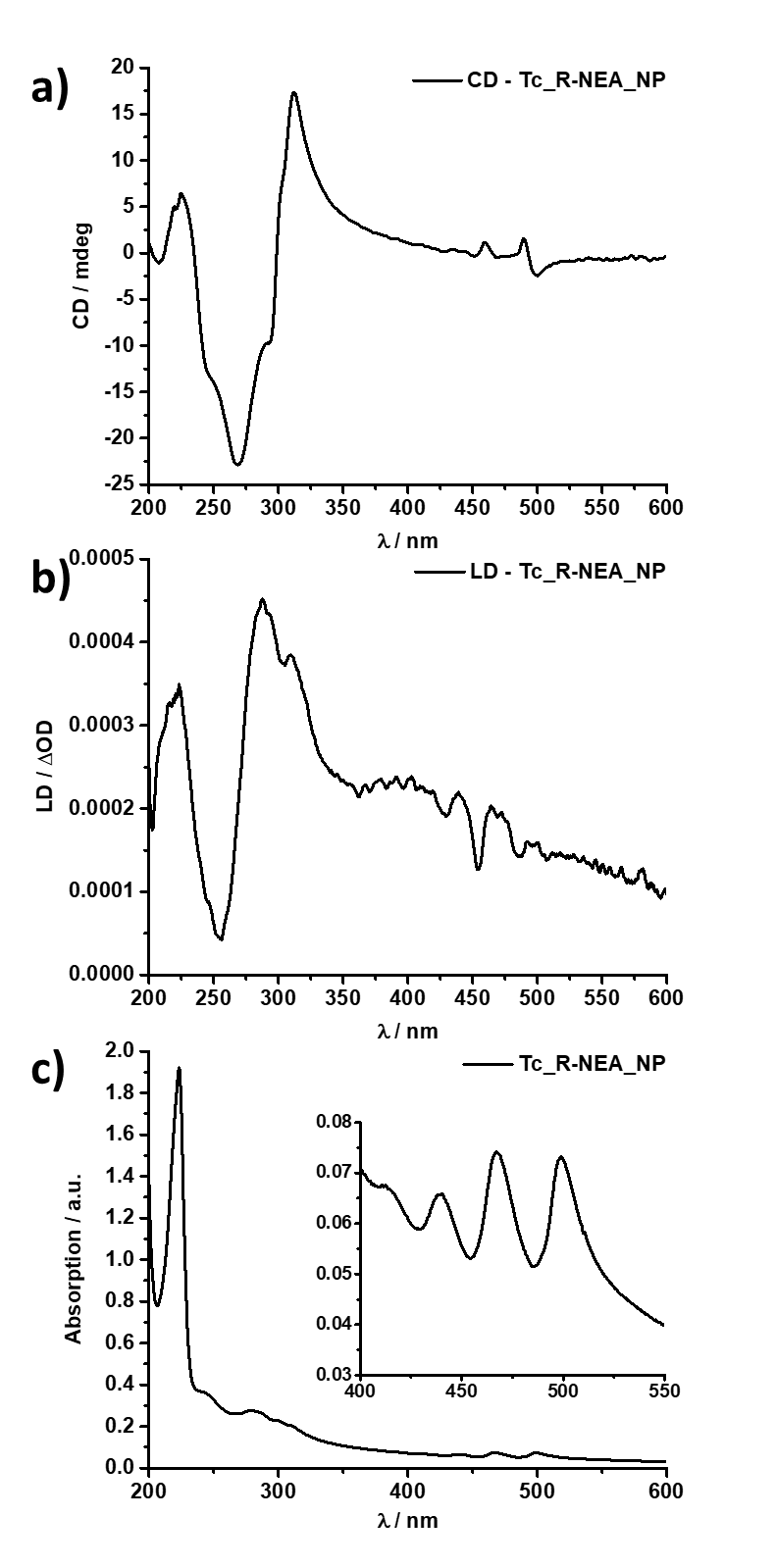


**Figure S26.** a) Circular dichroism (CD), b) linear dichroism spectra (LD) and c) corresponding absorption spectra of **Tc_R-NEA_NP**.


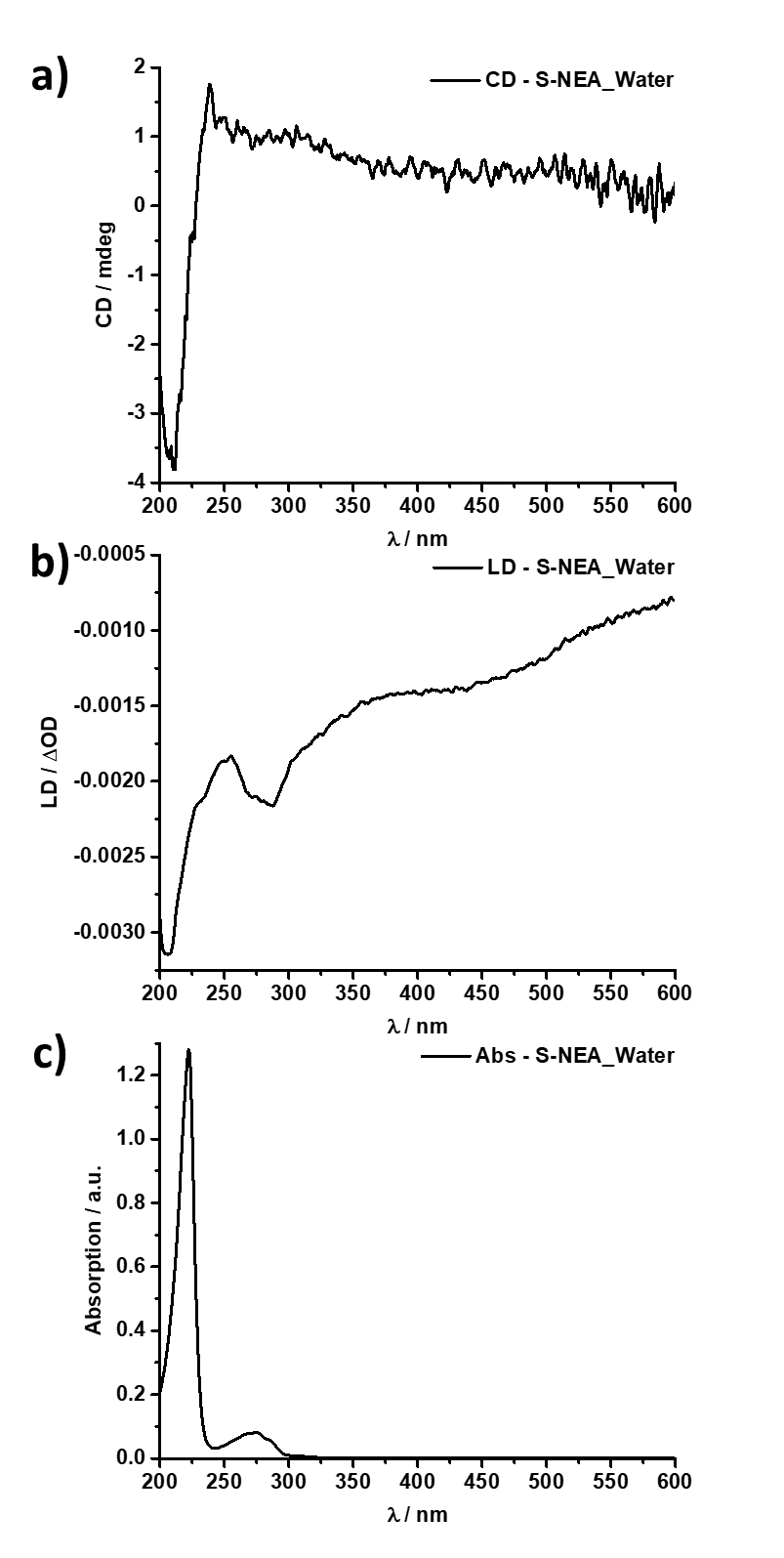


**Figure S27.** a) Circular dichroism (CD), b) linear dichroism spectra (LD) and c) corresponding absorption spectra of **S-NEA** in deionized water.


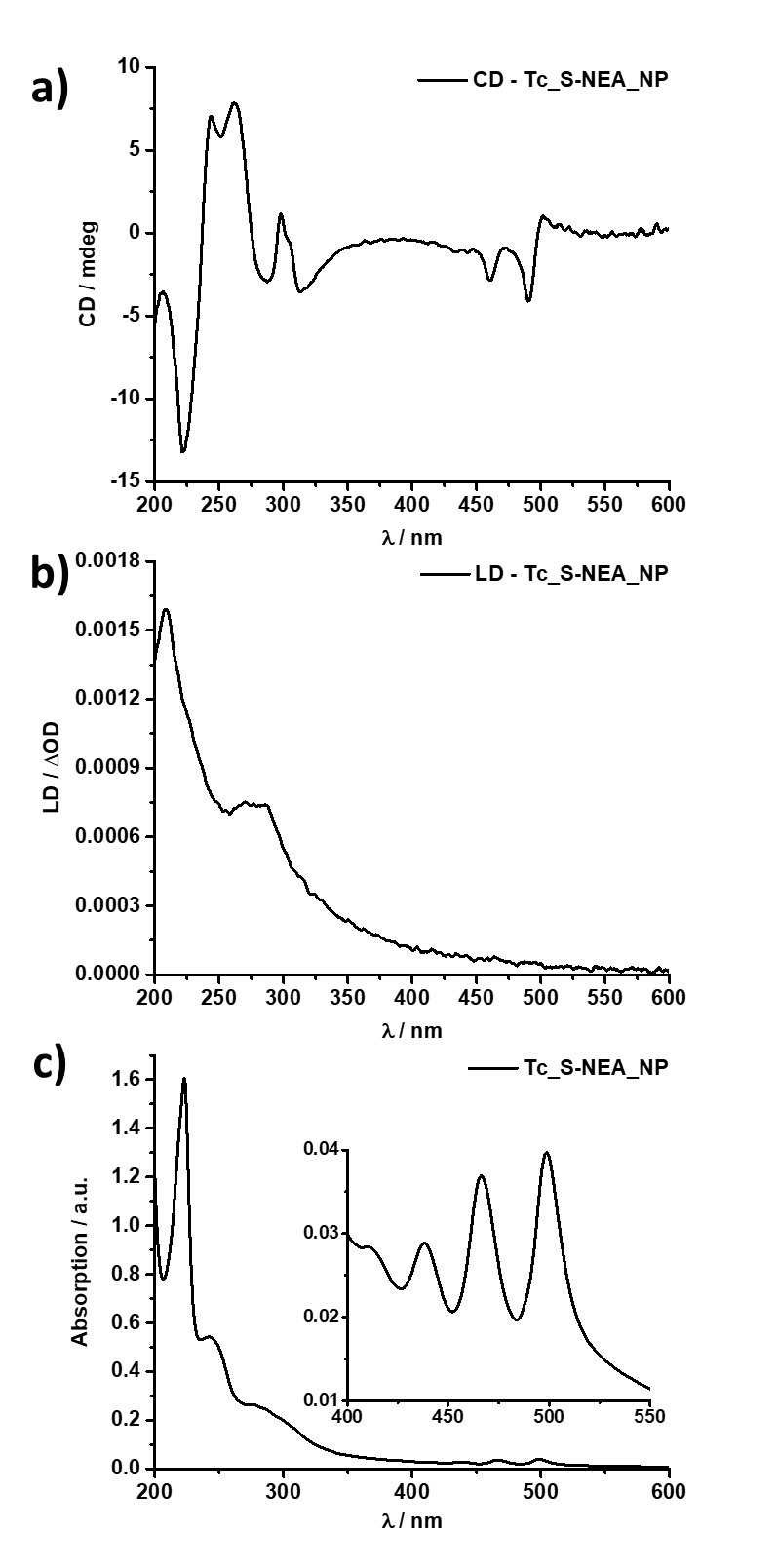


**Figure S28.** a) Circular dichroism (CD), b) linear dichroism spectra (LD) and c) corresponding absorption spectra of **Tc_S-NEA_NP**.


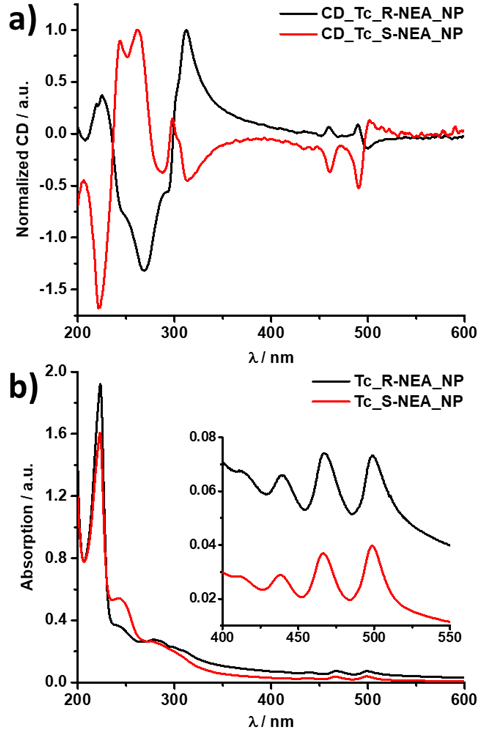


**Figure S29.** Circular dichroism (CD) spectra (a) and corresponding absorption spectra (b) of aqueous **Tc_R‑NEA_NP** (black) and **Tc_S-NEA_NP** (red).


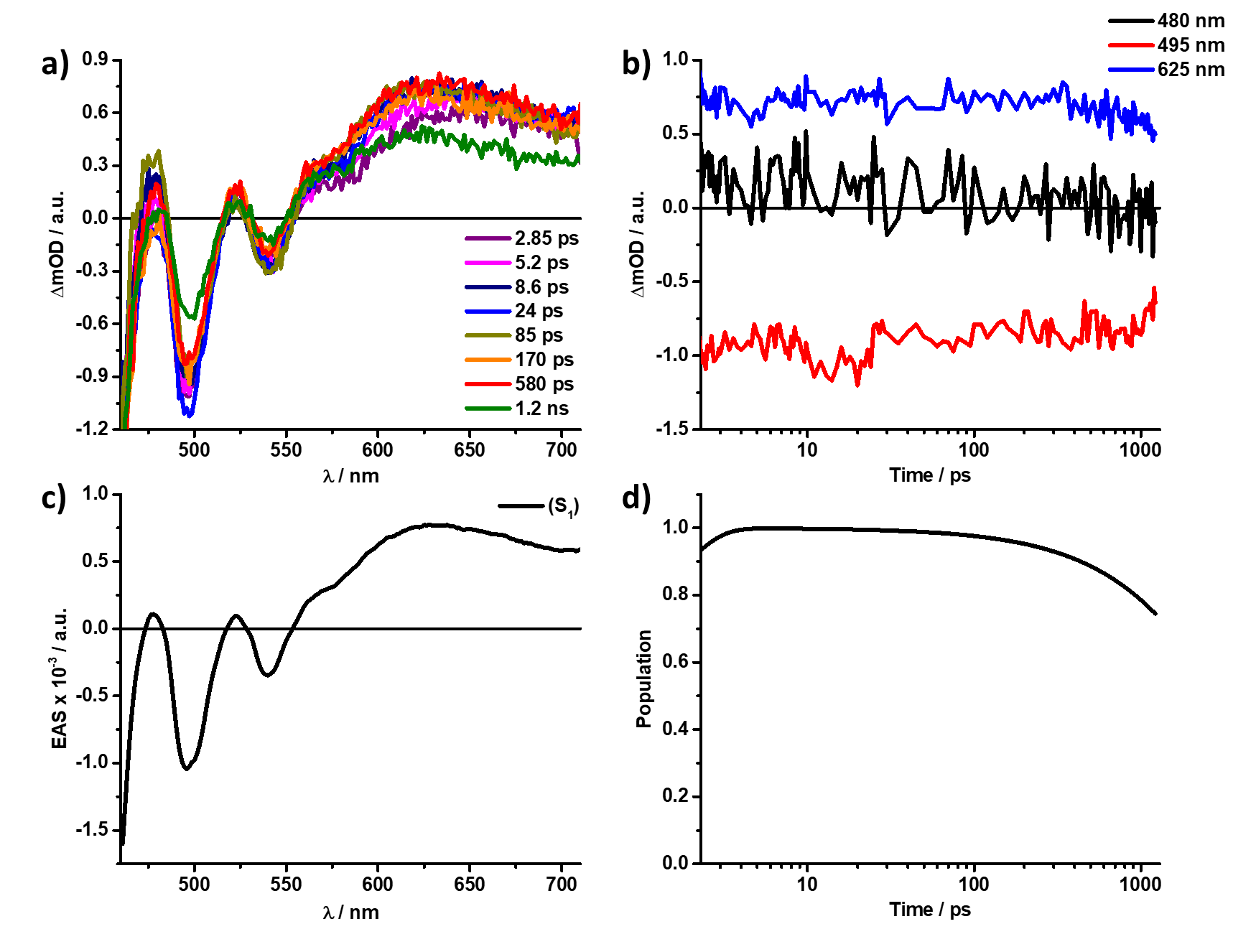


**Figure S30.** (a) Femtosecond transient absorption spectra (λ_ex_ = 400 nm) of **Tc** in THF at the indicated time delays, together with (b) the respective time absorption profiles at the indicated wavelengths. (c) Deconvoluted evolution-associated spectra (EAS) showcasing the singlet excited state (S_1_) (black) as obtained from global analysis. (d) Respective population kinetic of c).


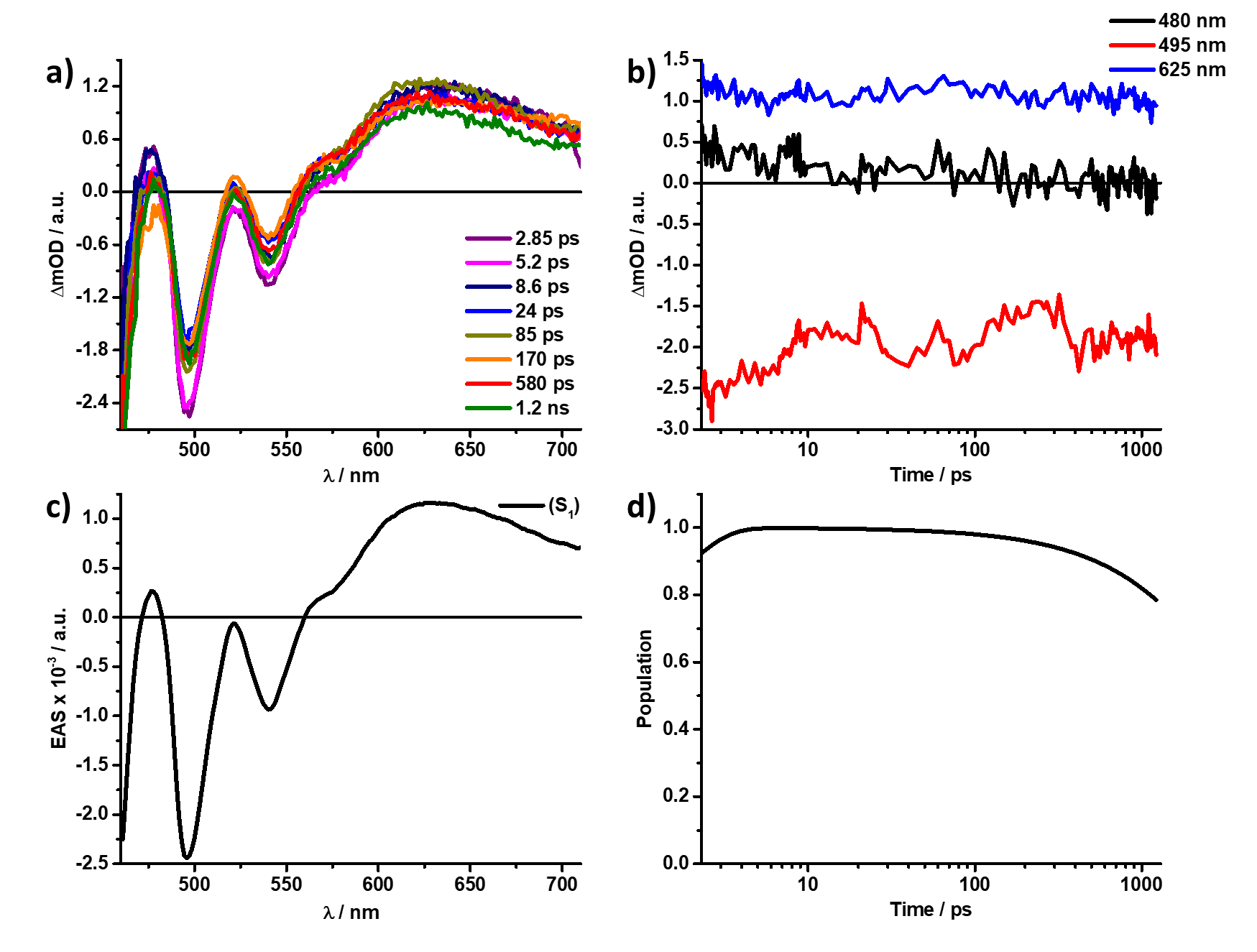


**Figure S31.** (a) Femtosecond transient absorption spectra (λ_ex_ = 400 nm) of **Tc_TPMA** salt in THF with time delays at the indicated time delays, together with (b) the respective time absorption profiles at the indicated wavelengths. (c) Deconvoluted evolution‑associated spectra (EAS) showcasing the singlet excited state (S_1_) (black) as obtained from global analysis. (d) Respective population kinetic of c).


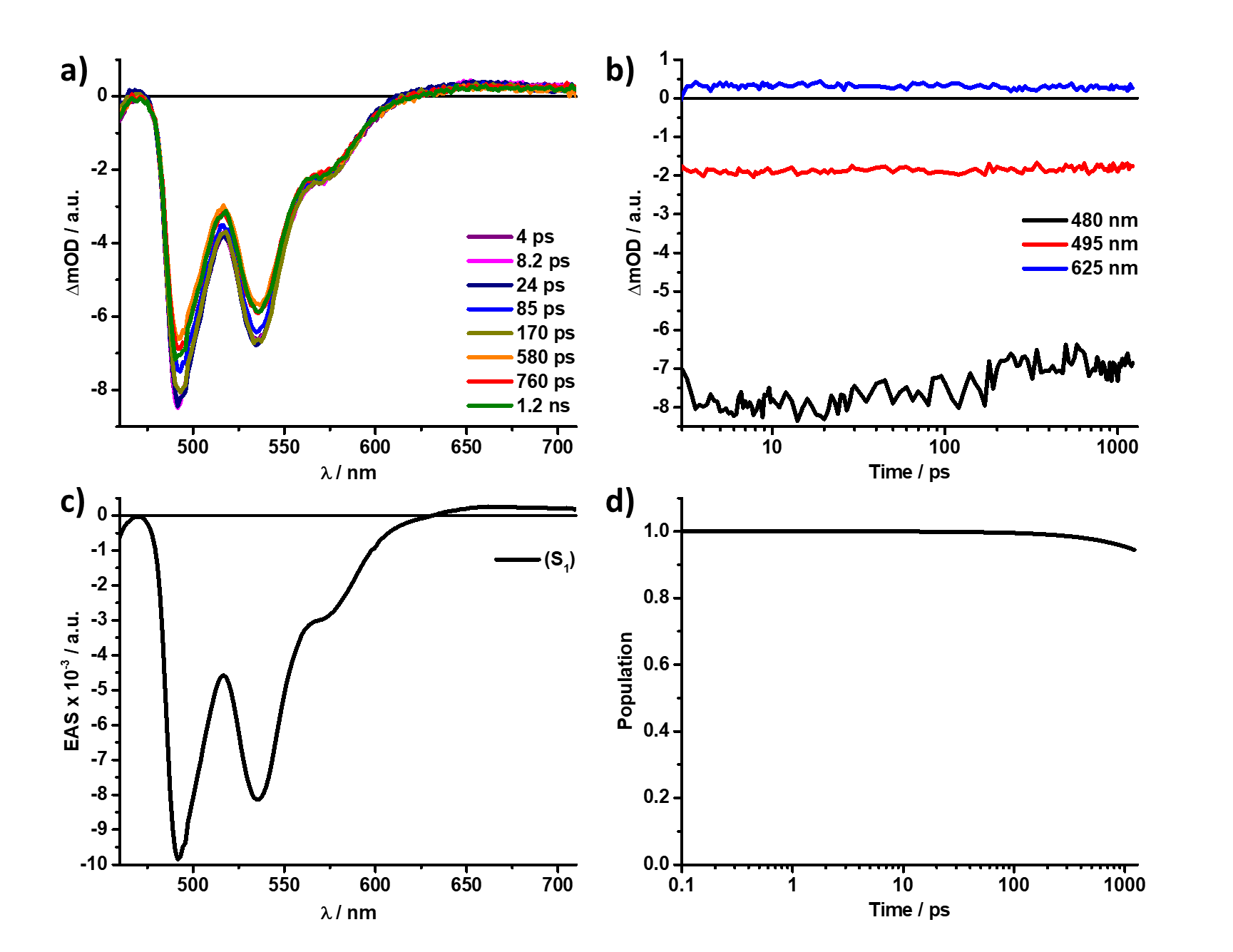


**Figure S32.** (a) Femtosecond transient absorption spectra (λ_ex_ = 400 nm) of **Tc_NMA** salt in THF at the indicated time delays, together with (b) the respective time absorption profiles at the indicated wavelengths. (c) Deconvoluted evolution‑associated spectra (EAS) showcasing the singlet excited state (S_1_) (black) as obtained from global analysis. (d) Respective population kinetic of c).


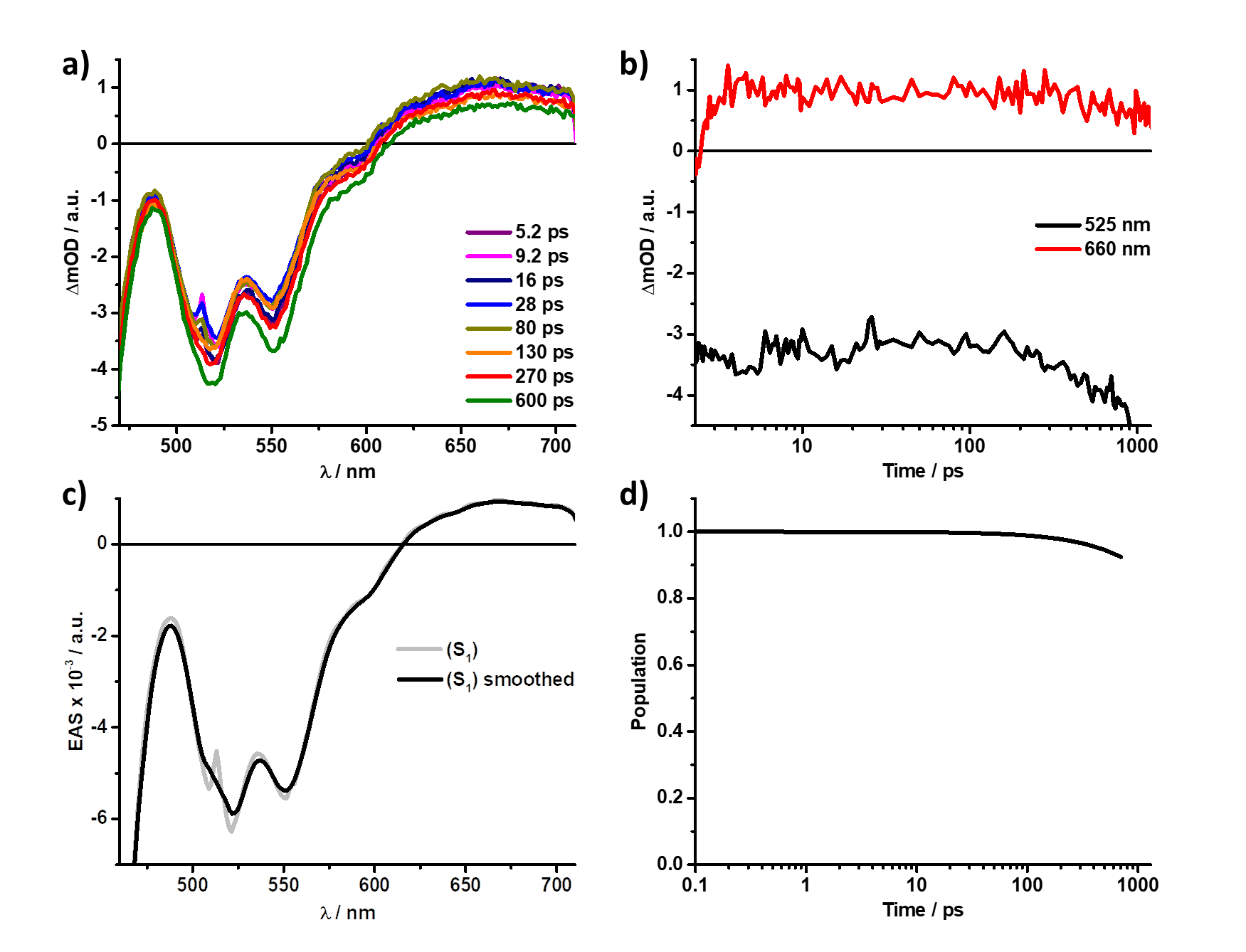


**Figure S33.** (a) Femtosecond transient absorption spectra (λ_ex_ = 400 nm) of **Tc_CyHx** salt in THF at the indicated time delays, together with (b) the respective time absorption profiles at the indicated wavelengths. (c) Deconvoluted evolution‑associated spectra (EAS) showcasing the singlet excited state (S_1_) (black; smoothed curve) as obtained from global analysis. (d) Respective population kinetic of c).


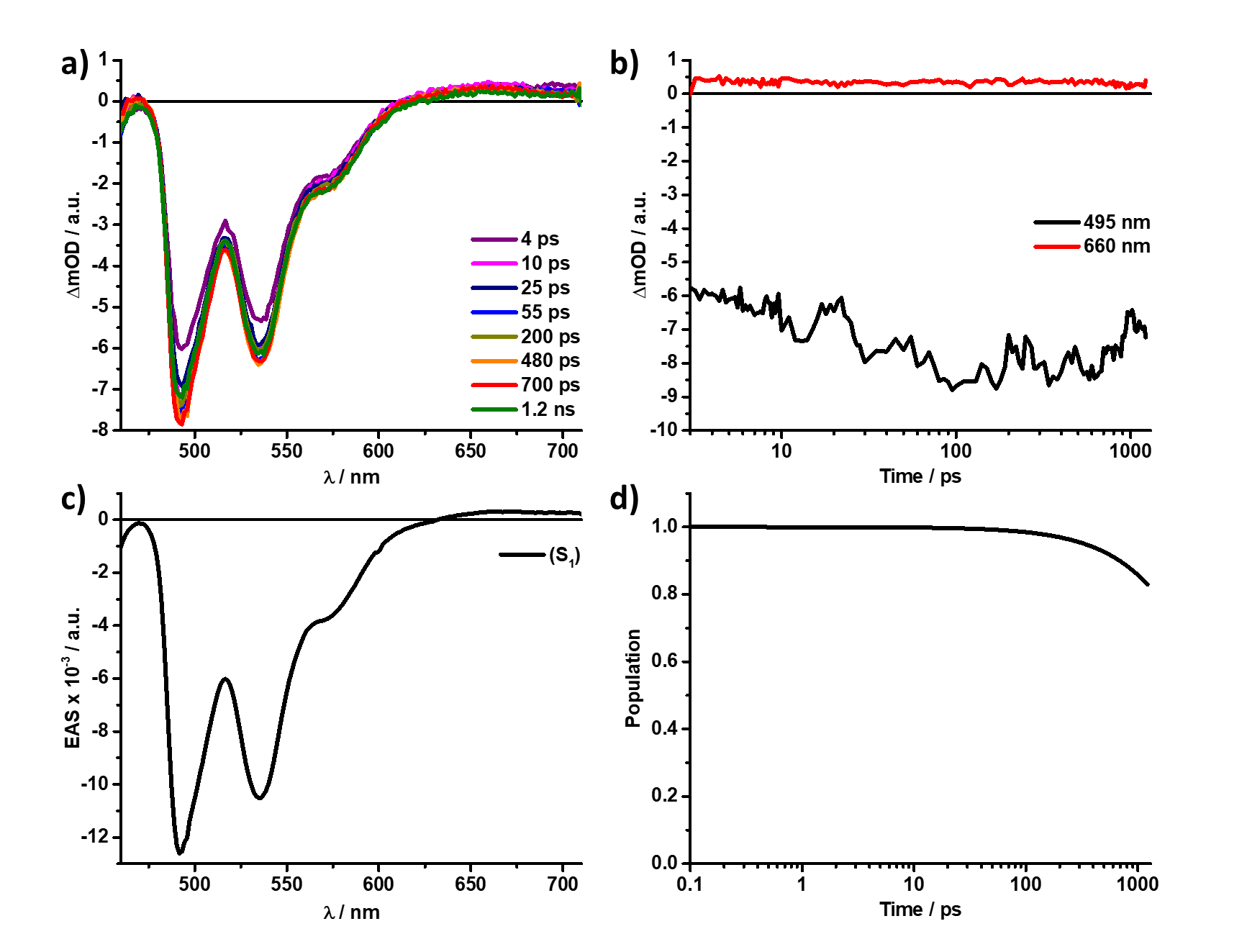


**Figure S34.** (a) Femtosecond transient absorption spectra (λ_ex_ = 400 nm) of **Tc_R-NEA** salt in THF at the indicated time delays, together with (b) the respective time absorption profiles at the indicated wavelengths. (c) Deconvoluted evolution‑associated spectra (EAS) showcasing the singlet excited state (S_1_) (black; smoothed curve) as obtained from global analysis. (d) Respective population kinetic of c).


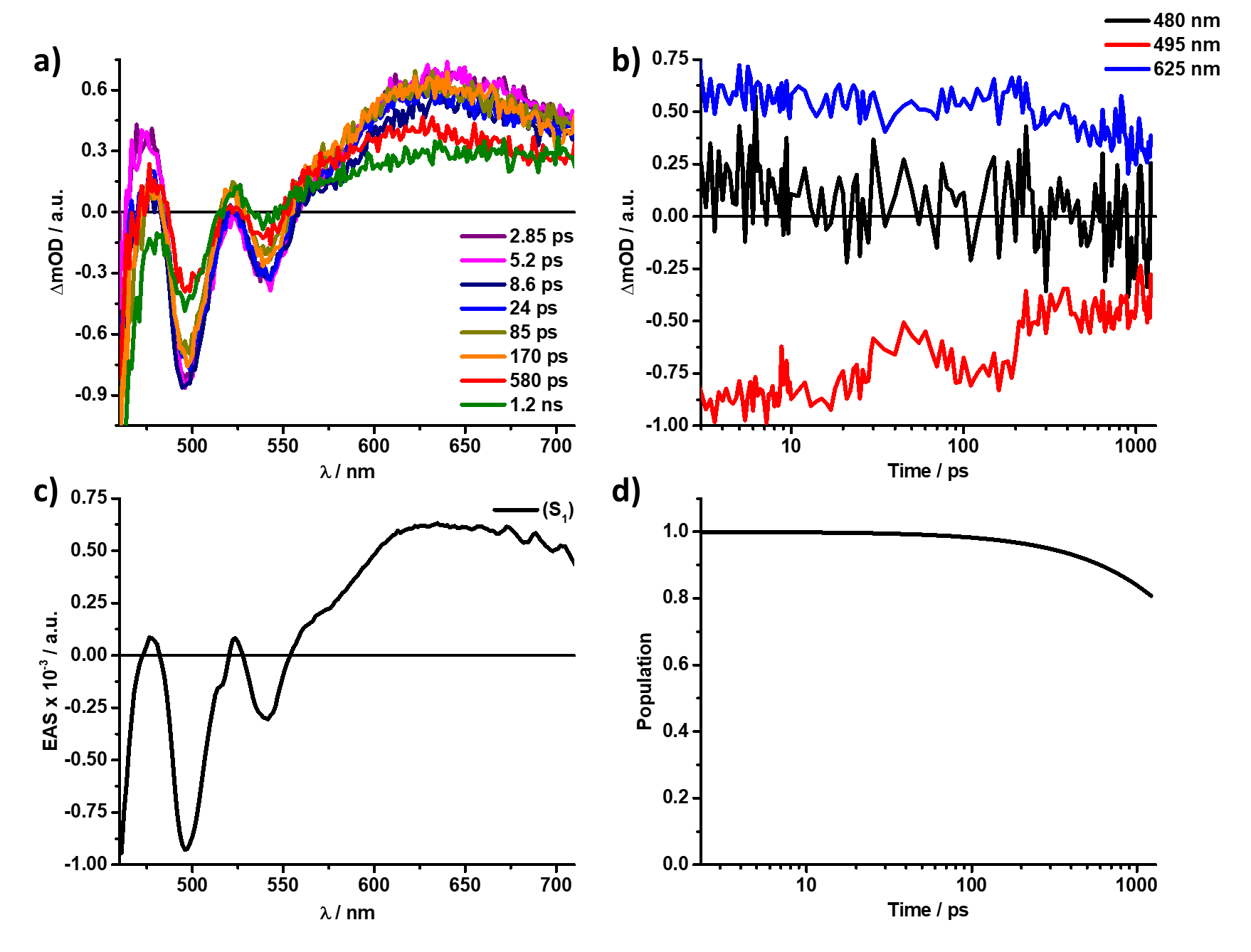


**Figure S35.** (a) Femtosecond transient absorption spectra (λ_ex_ = 400 nm) of **Tc_S-NEA** salt in THF at the indicated time delays, together with (b) the respective time absorption profiles at the indicated wavelengths. (c) Deconvoluted evolution‑associated spectra (EAS) showcasing the singlet excited state (S_1_) (black; smoothed curve) as obtained from global analysis. (d) Respective population kinetic of c).


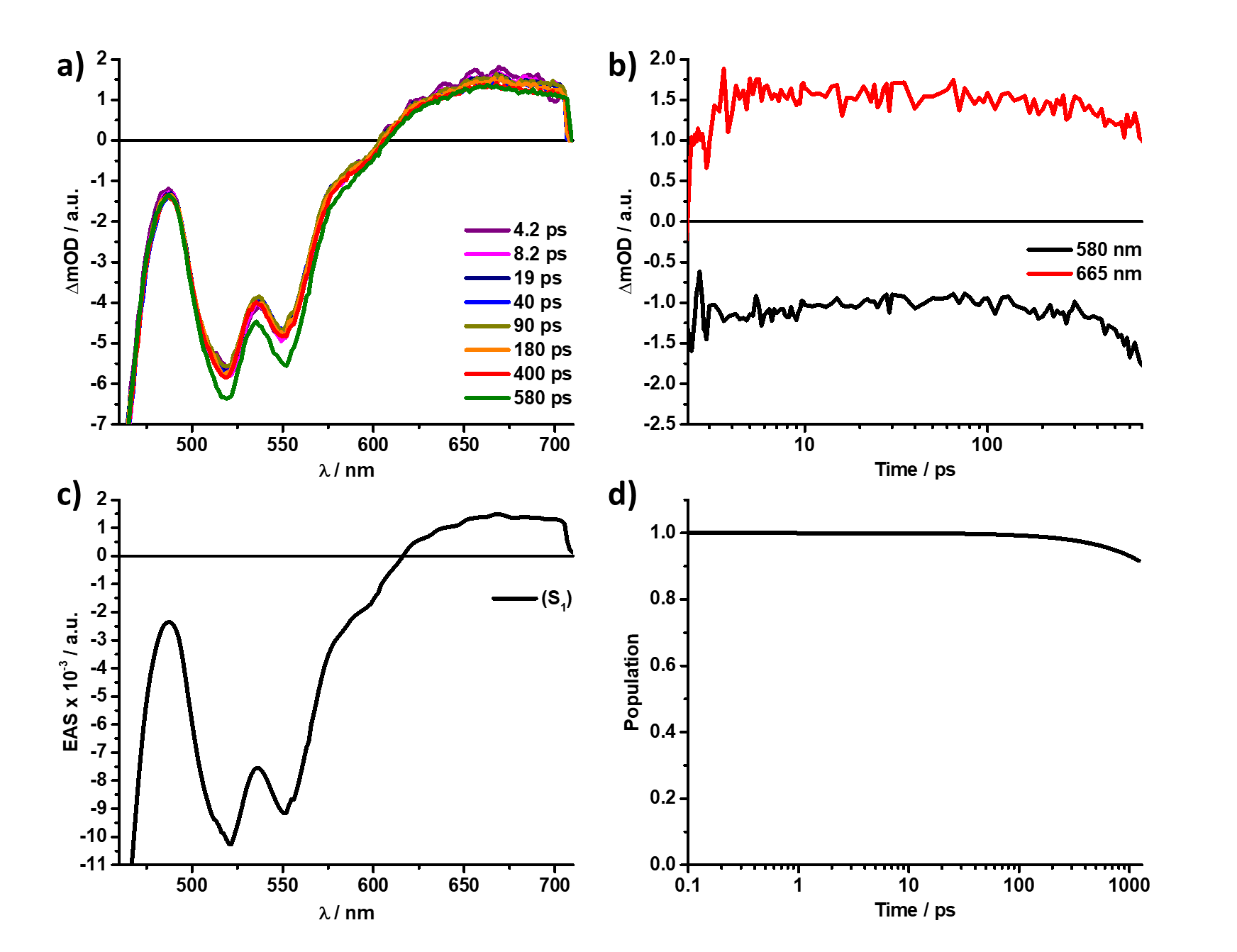


**Figure S36.** (a) Femtosecond transient absorption spectra (λ_ex_ = 400 nm) of **Tc_Rac** salt in THF at the indicated time delays, together with (b) the respective time absorption profiles at the indicated wavelengths. (c) Deconvoluted evolution‑associated spectra (EAS) showcasing the singlet excited state (S_1_) (black; smoothed curve) as obtained from global analysis. (d) Respective population kinetic of c).


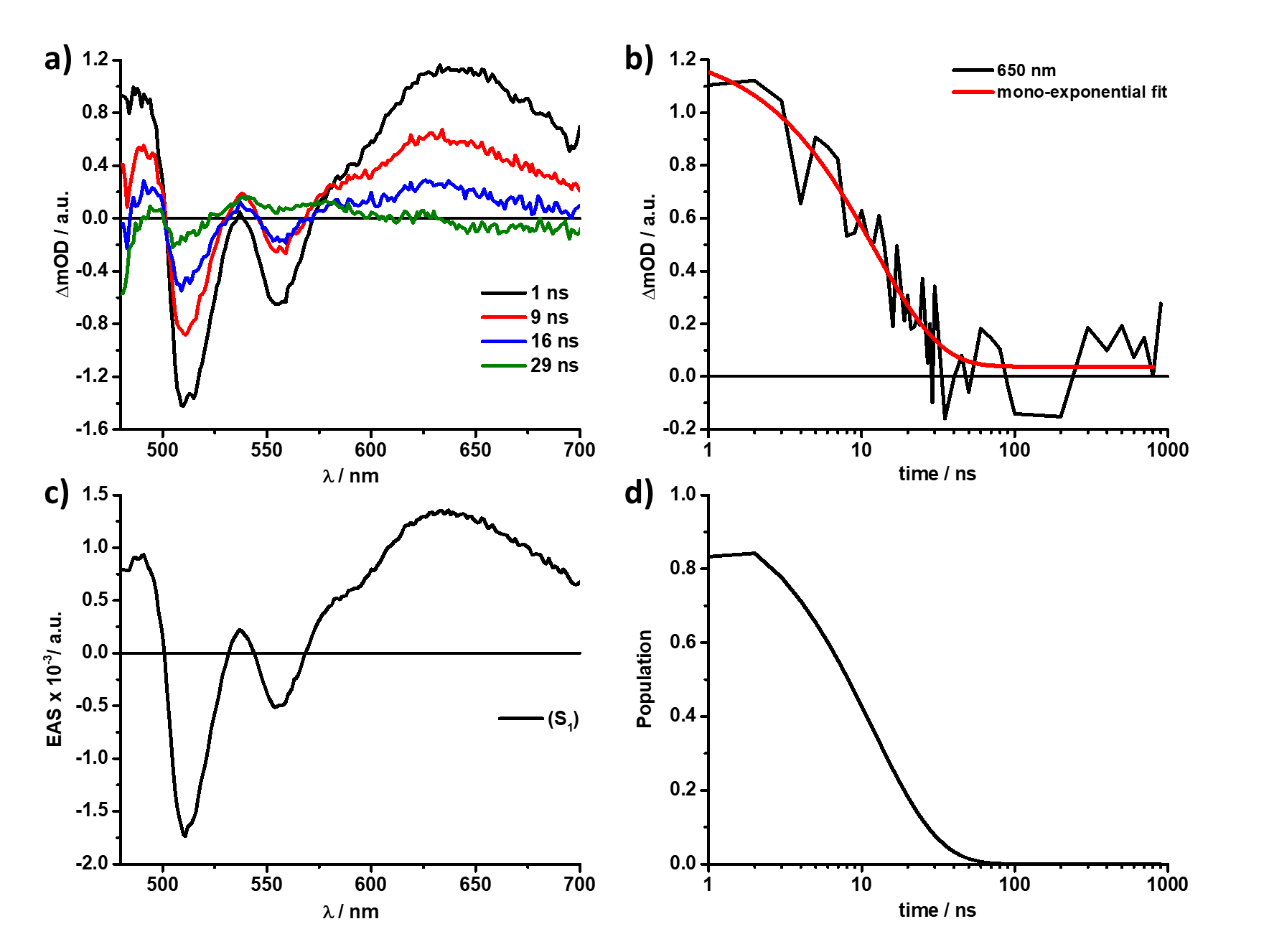


**Figure S37.** (a) Nanosecond transient absorption spectra (λ_ex_ = 410 nm) of **Tc** in THF at the indicated time delays, together with (b) the respective time absorption profile and fit at the indicated wavelength. (c) Deconvoluted evolution-associated spectra (EAS) showcasing the singlet excited state (S_1_) (black) as obtained from global analysis. (d) Respective population kinetic of c).


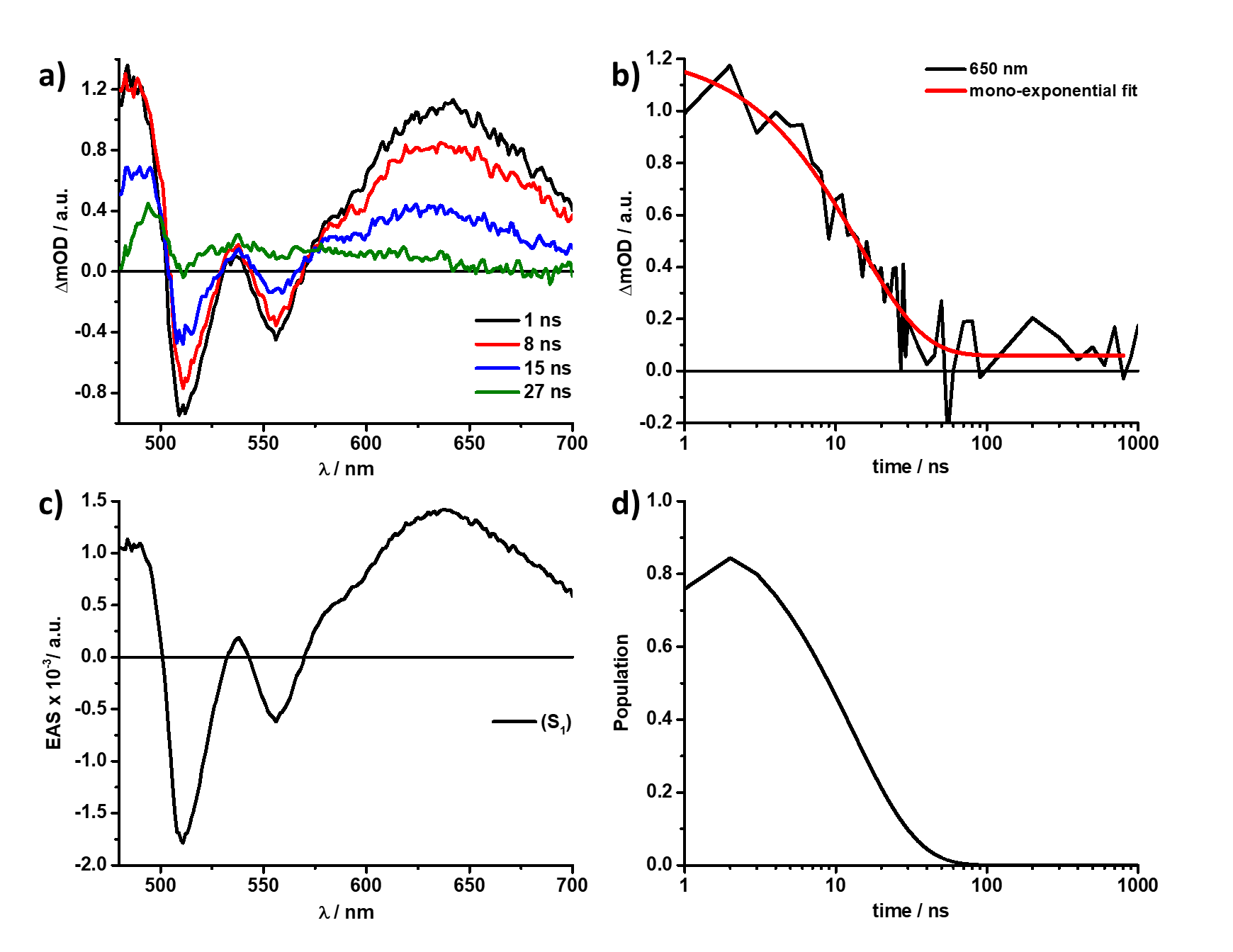


**Figure S38.** (a) Nanosecond transient absorption spectra (λ_ex_ = 410 nm) of **Tc_TPMA** salt in THF at the indicated time delays, together with (b) the respective time absorption profile and fit at the indicated wavelength. (c) Deconvoluted evolution-associated spectra (EAS) showcasing the singlet excited state (S_1_) (black) as obtained from global analysis. (d) Respective population kinetic of c).


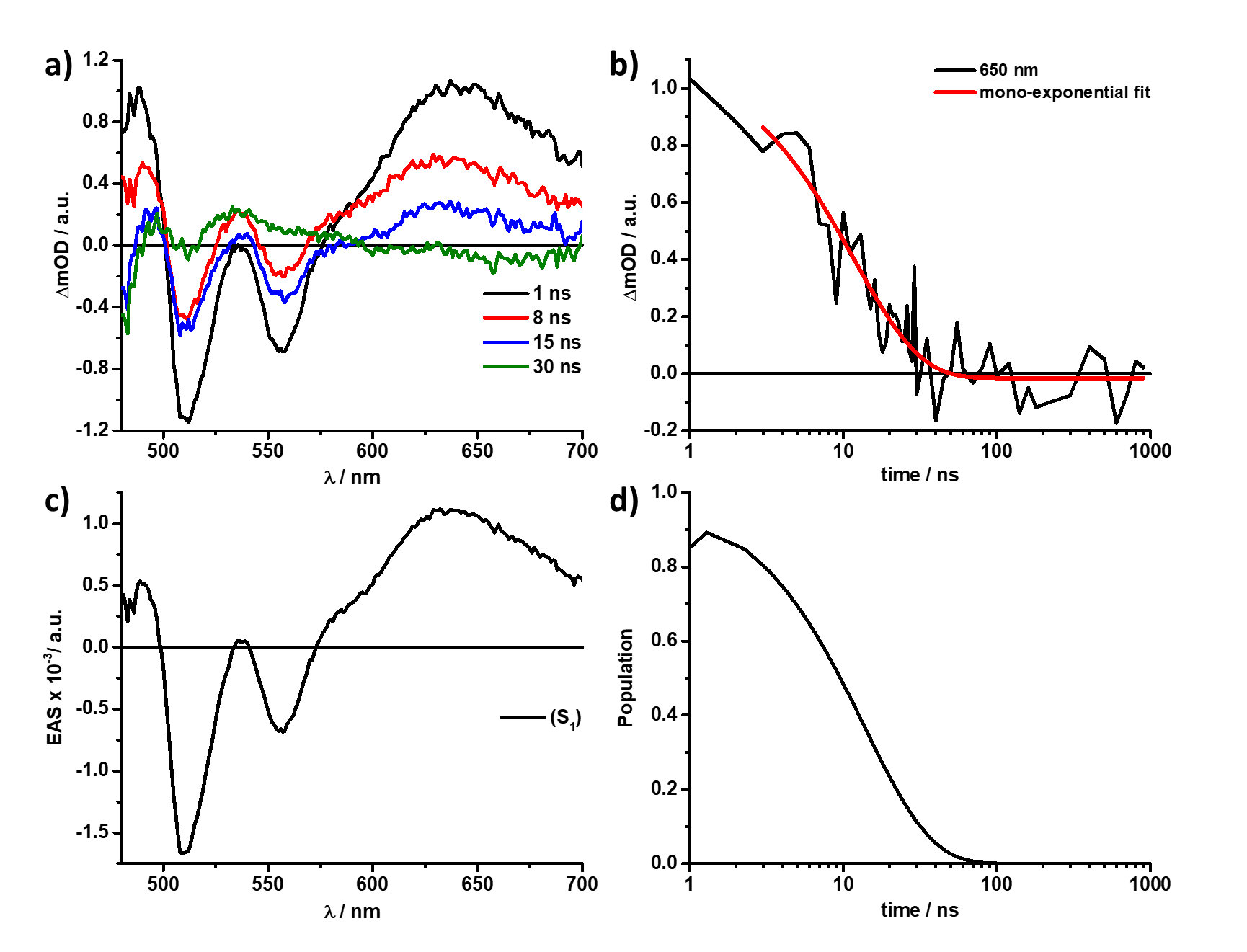


**Figure S39.** (a) Nanosecond transient absorption spectra (λ_ex_ = 410 nm) of **Tc_NMA** salt in THF at the indicated time delays, together with (b) the respective time absorption profile and fit at the indicated wavelength. (c) Deconvoluted evolution-associated spectra (EAS) showcasing the singlet excited state (S_1_) (black) as obtained from global analysis. (d) Respective population kinetic of c).


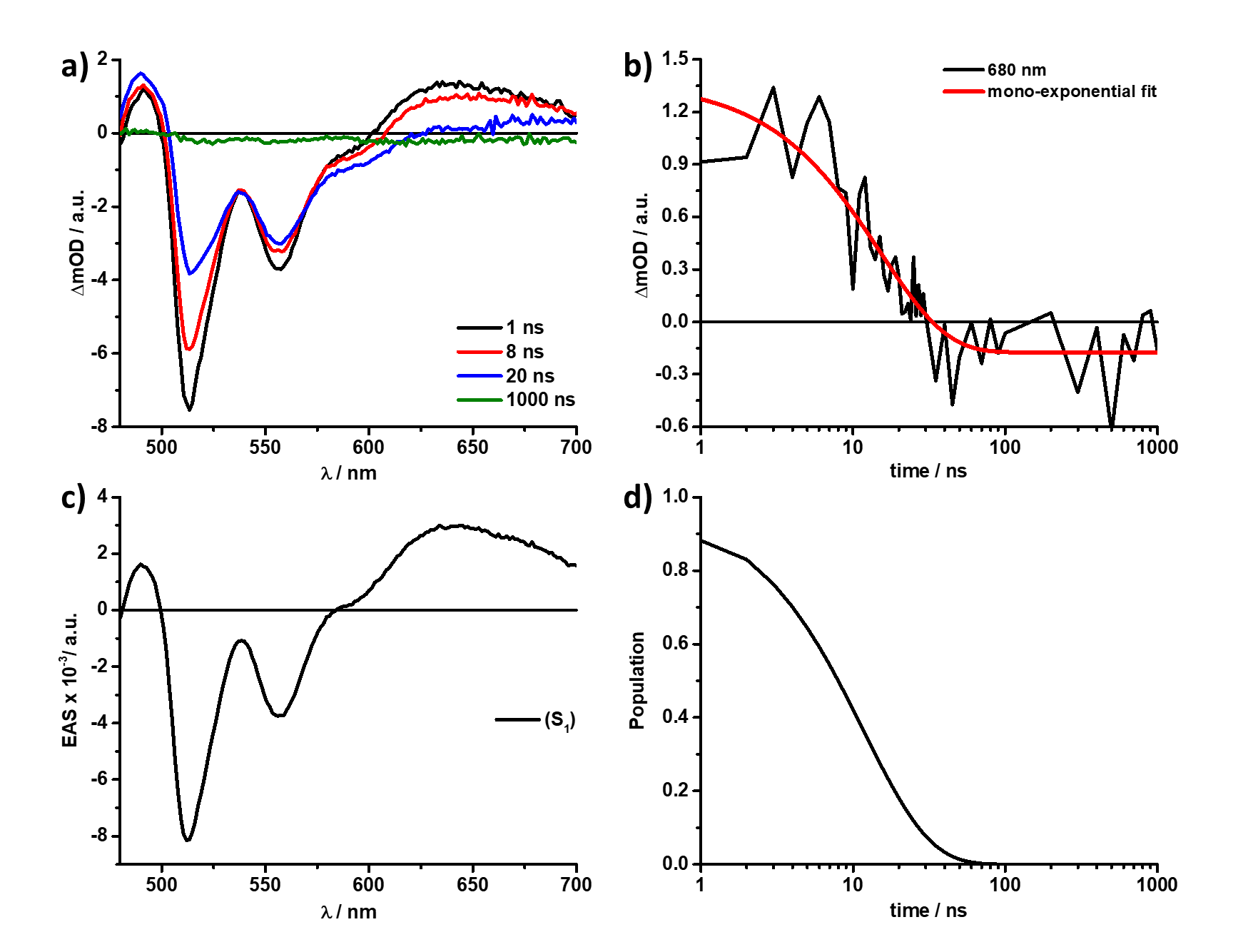


**Figure S40.** (a) Nanosecond transient absorption spectra (λ_ex_ = 410 nm) of **Tc_CyHx** salt in THF at the indicated time delays, together with (b) the respective time absorption profile and fit at the indicated wavelength. (c) Deconvoluted evolution-associated spectra (EAS) showcasing the singlet excited state (S_1_) (black) as obtained from global analysis. (d) Respective population kinetic of c).


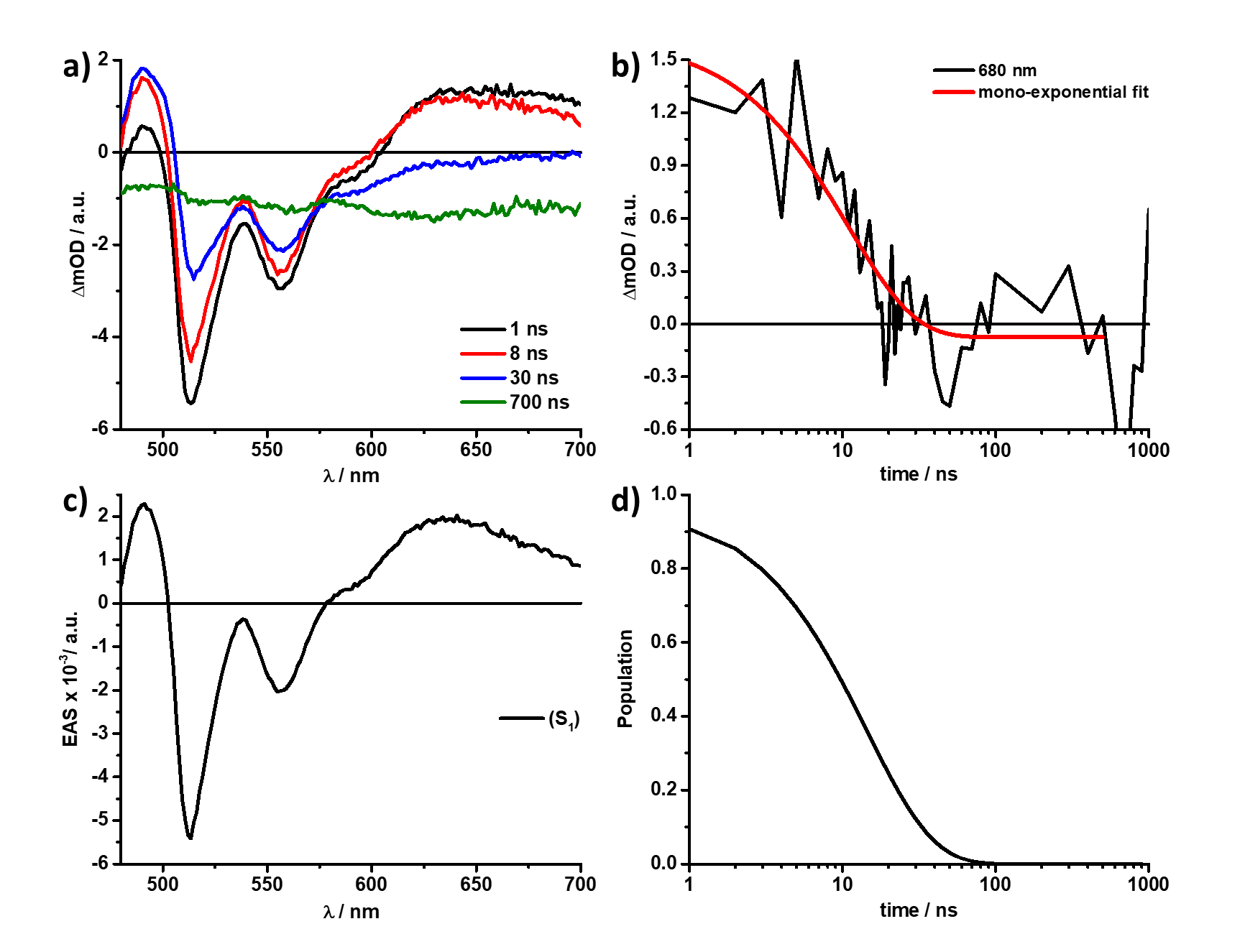


**Figure S41.** (a) Nanosecond transient absorption spectra (λ_ex_ = 410 nm) of **Tc_R-NEA** salt in THF at the indicated time delays, together with (b) the respective time absorption profile and fit at the indicated wavelength. (c) Deconvoluted evolution-associated spectra (EAS) showcasing the singlet excited state (S_1_) (black) as obtained from global analysis. (d) Respective population kinetic of c).


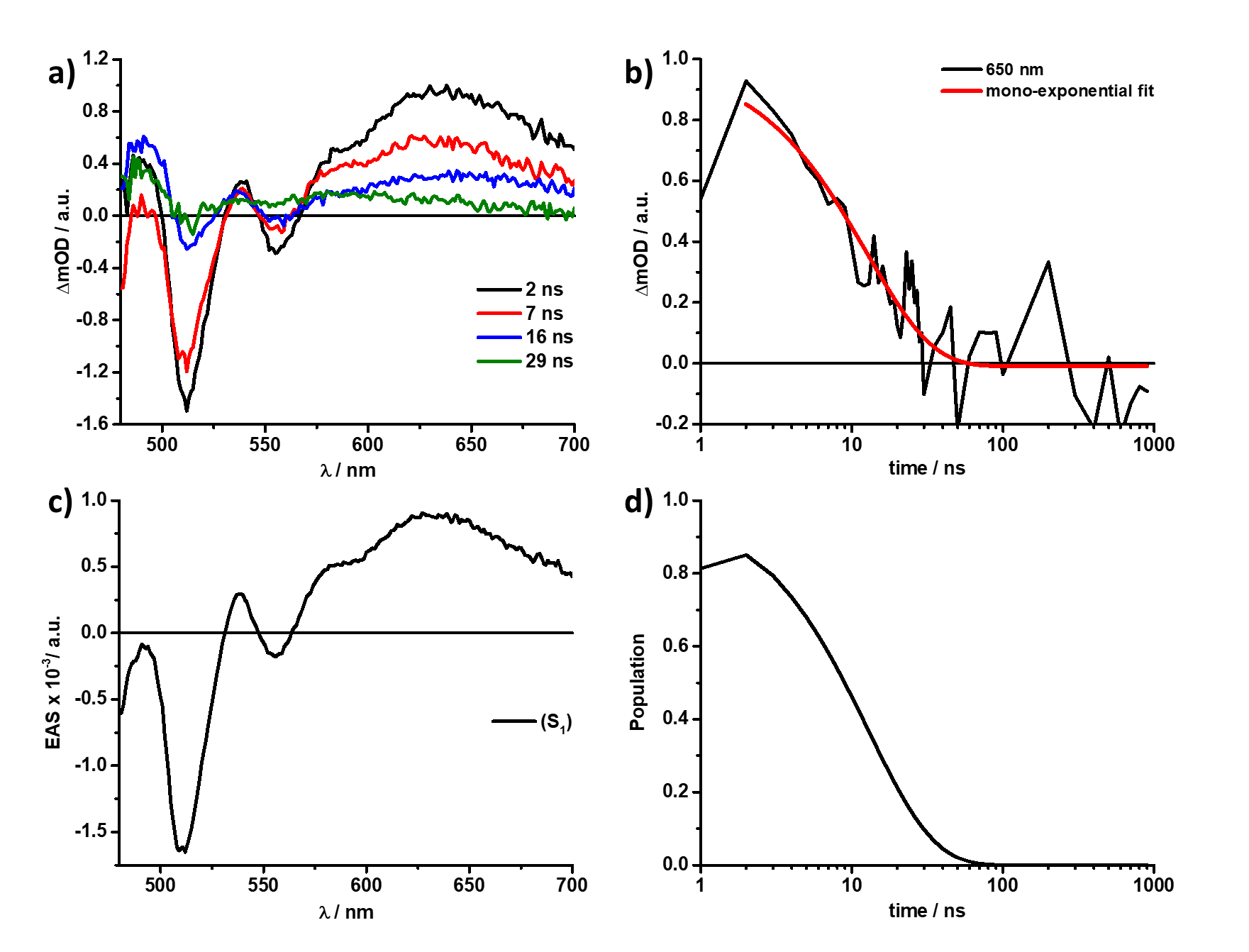


**Figure S42.** (a) Nanosecond transient absorption spectra (λ_ex_ = 410 nm) of **Tc_S-NEA** salt in THF at the indicated time delays, together with (b) the respective time absorption profile and fit at the indicated wavelength. (c) Deconvoluted evolution-associated spectra (EAS) showcasing the singlet excited state (S_1_) (black) as obtained from global analysis. (d) Respective population kinetic of c).


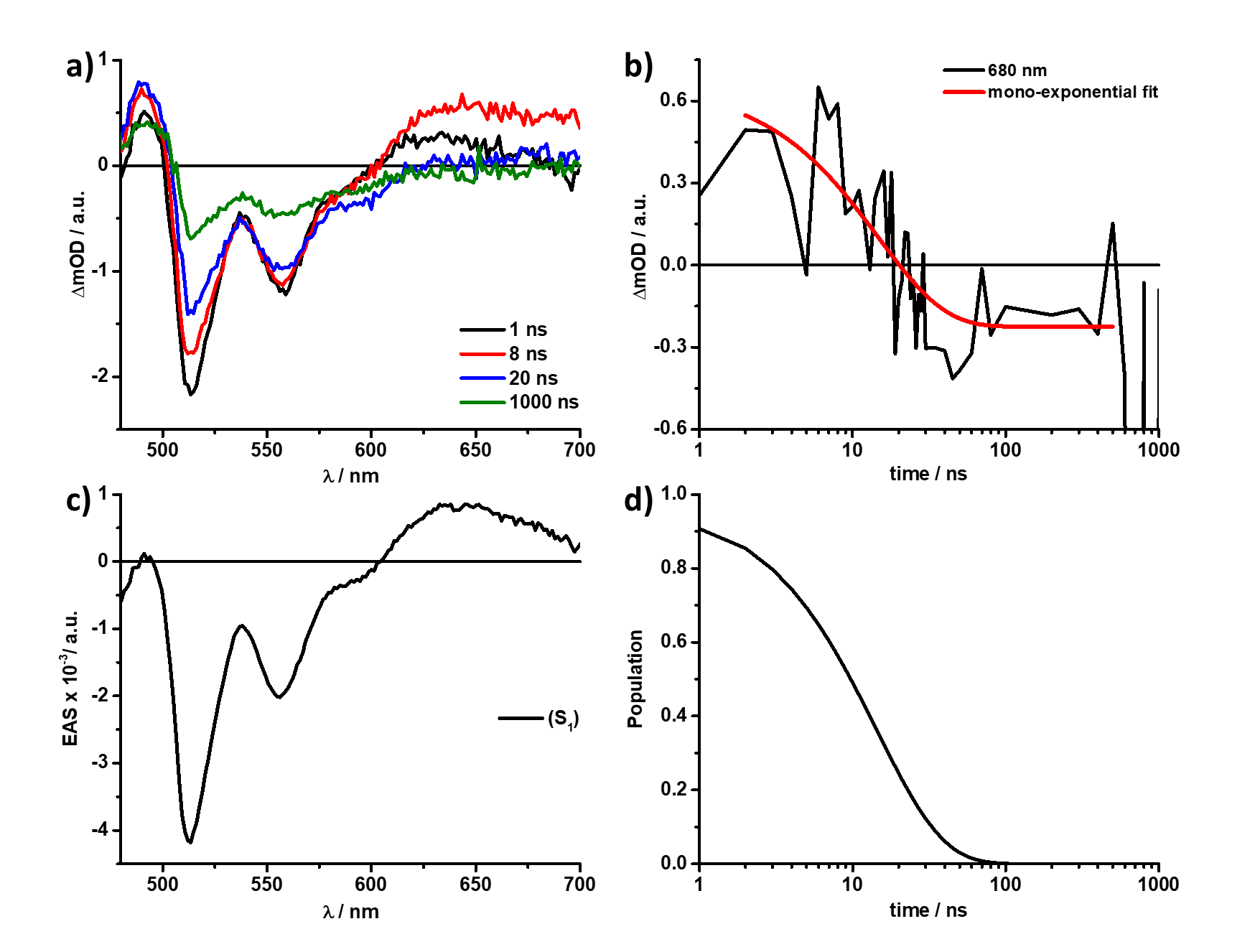


**Figure S43.** (a) Nanosecond transient absorption spectra (λ_ex_ = 410 nm) of **Tc_Rac** salt in THF at the indicated time delays, together with (b) the respective time absorption profile and fit at the indicated wavelength. (c) Deconvoluted evolution-associated spectra (EAS) showcasing the singlet excited state (S_1_) (black) as obtained from global analysis. (d) Respective population kinetic of c).


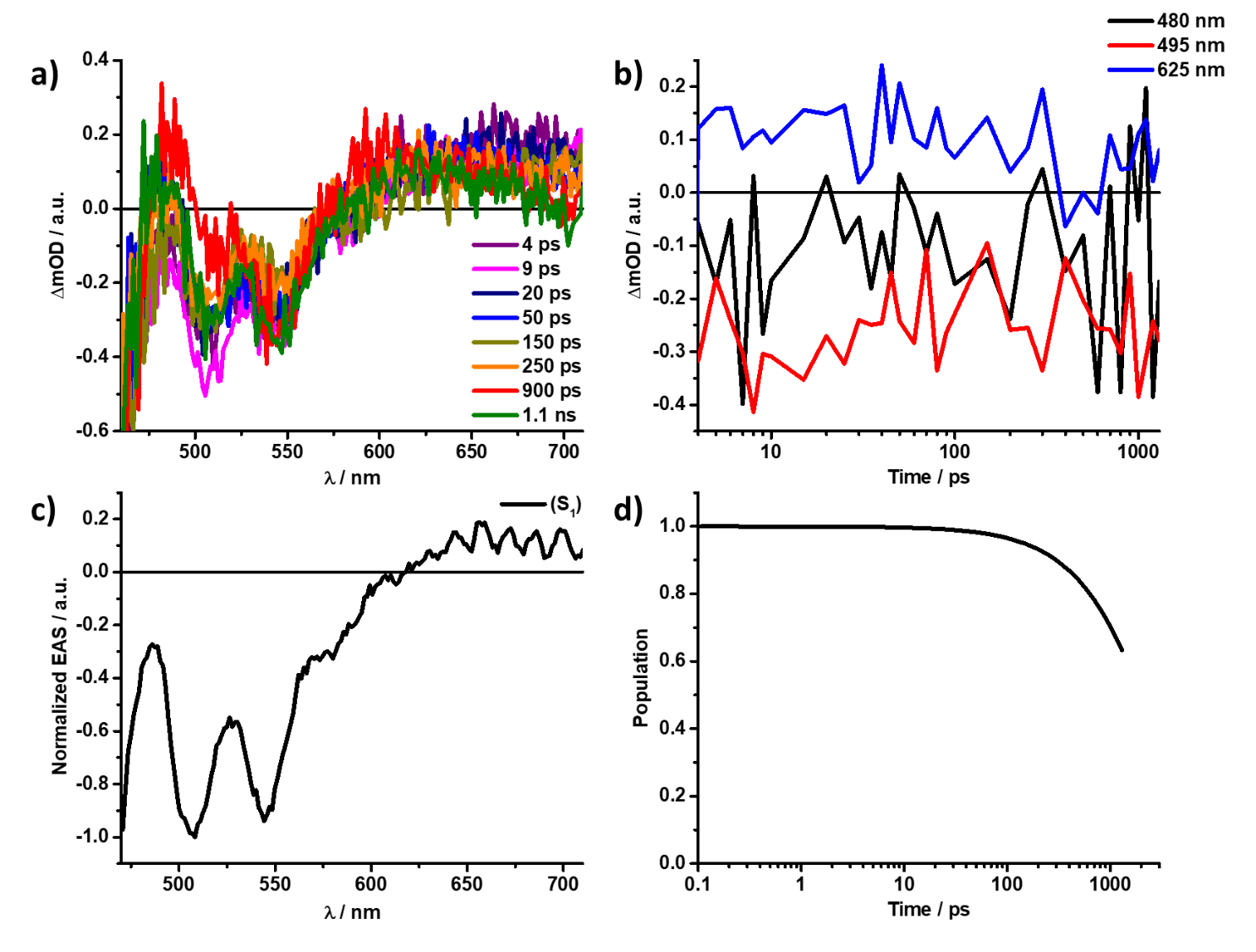


**Figure S44.** (a) Femtosecond transient absorption spectra (λ_ex_ = 400 nm) of aqueous **Tc_TPMA_NP** at the indicated time delays, together with (b) the respective time absorption profiles at the indicated wavelengths. (c) Deconvoluted evolution-associated spectra (EAS) showcasing the singlet excited state (S_1_) (black) as obtained from global analysis. (d) Respective population kinetic of c).

The stability of the aqueous **Tc_TPMA_NP**s under laser irradiation was relatively low, and the opaque nature of the nanoparticle dispersions and reflections of the laser beam led to a low signal-to-noise ratio. In addition, the broad absorption of the nanoparticle dispersion due to aggregation led to stronger GSB, slightly obscuring the maxima found at 480 and 520 nm in THF.


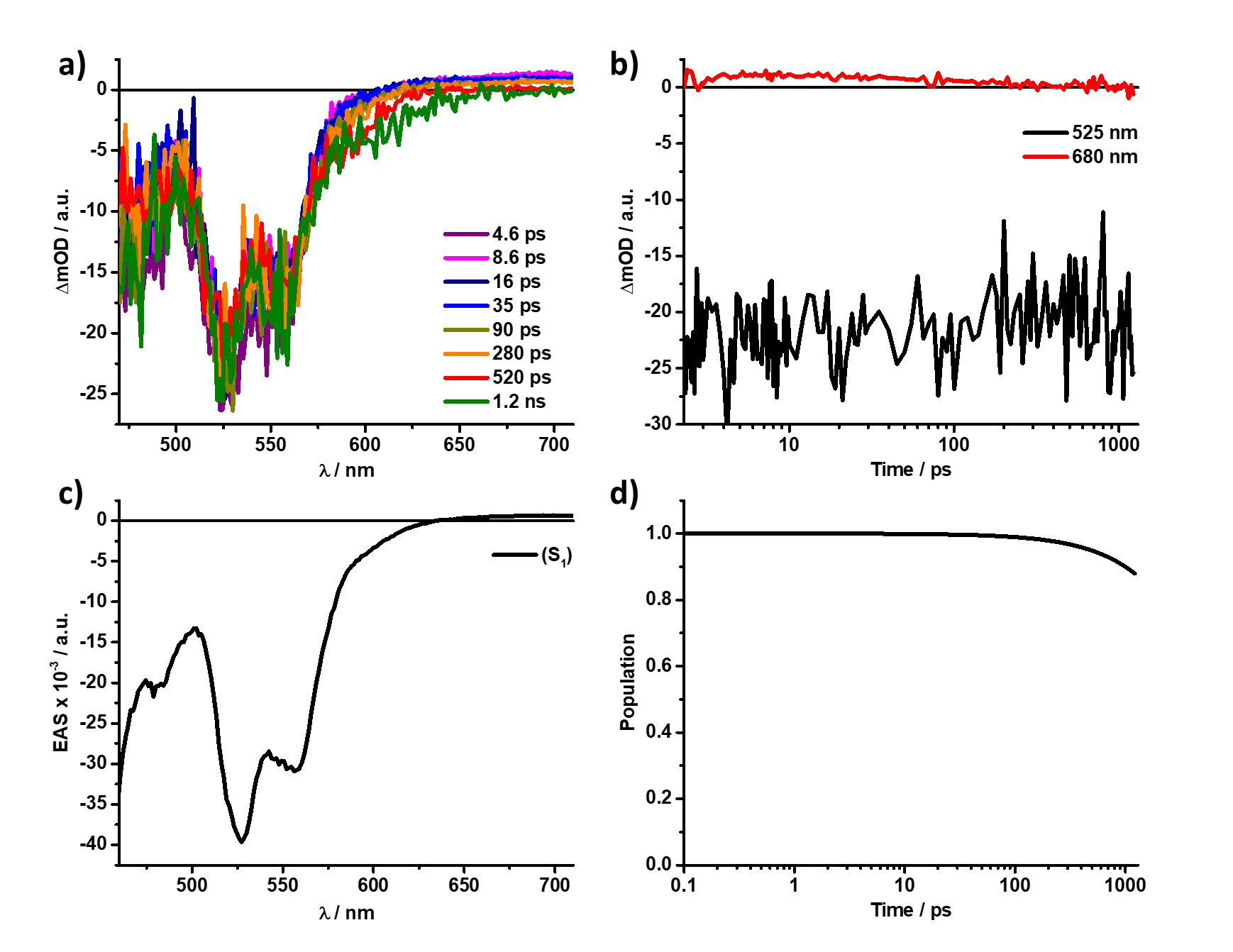


**Figure S45.** (a) Femtosecond transient absorption spectra (λ_ex_ = 400 nm) of aqueous **Tc_NMA_NP** at the indicated time delays, together with (b) the respective time absorption profiles at the indicated wavelengths. (c) Deconvoluted evolution‑associated spectra (EAS) showcasing the singlet excited state (S_1_) (black) as obtained from global analysis. (d) Respective population kinetic of c). Due to the low stability of the nanoparticle solution, the signal‑to‑noise ratio was rather low.


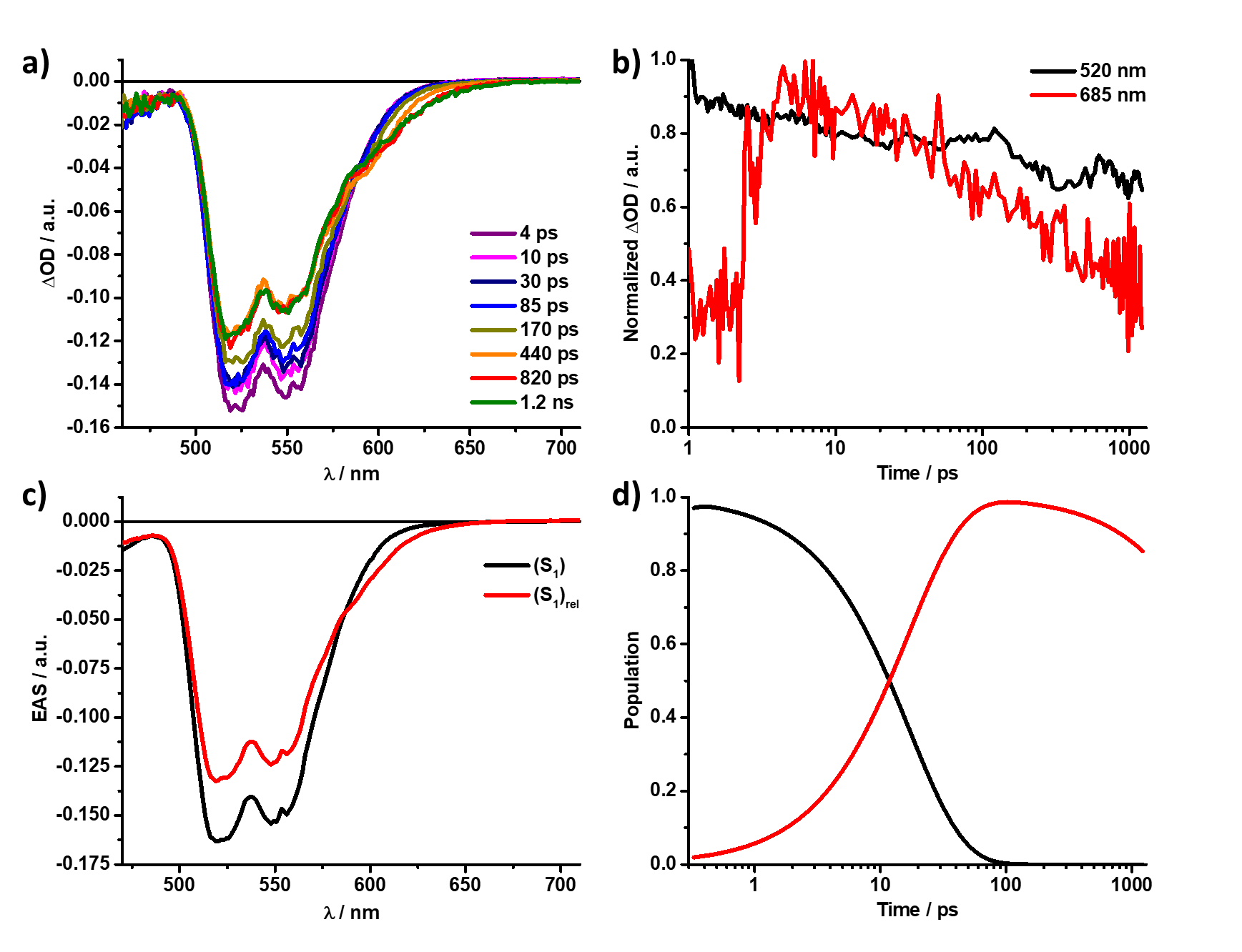


**Figure S46.** (a) Femtosecond transient absorption spectra (λ_ex_ = 400 nm) of aqueous **Tc_CyHx_NP** at the indicated time delays, together with (b) the respective time absorption profiles at the indicated wavelengths. (c) Deconvoluted evolution‑associated spectra (EAS) showcasing the singlet excited state (S_1_) (black) and relaxed singlet excited state (S­_1_)_rel_ as obtained from global analysis. (d) Respective population kinetics of c).


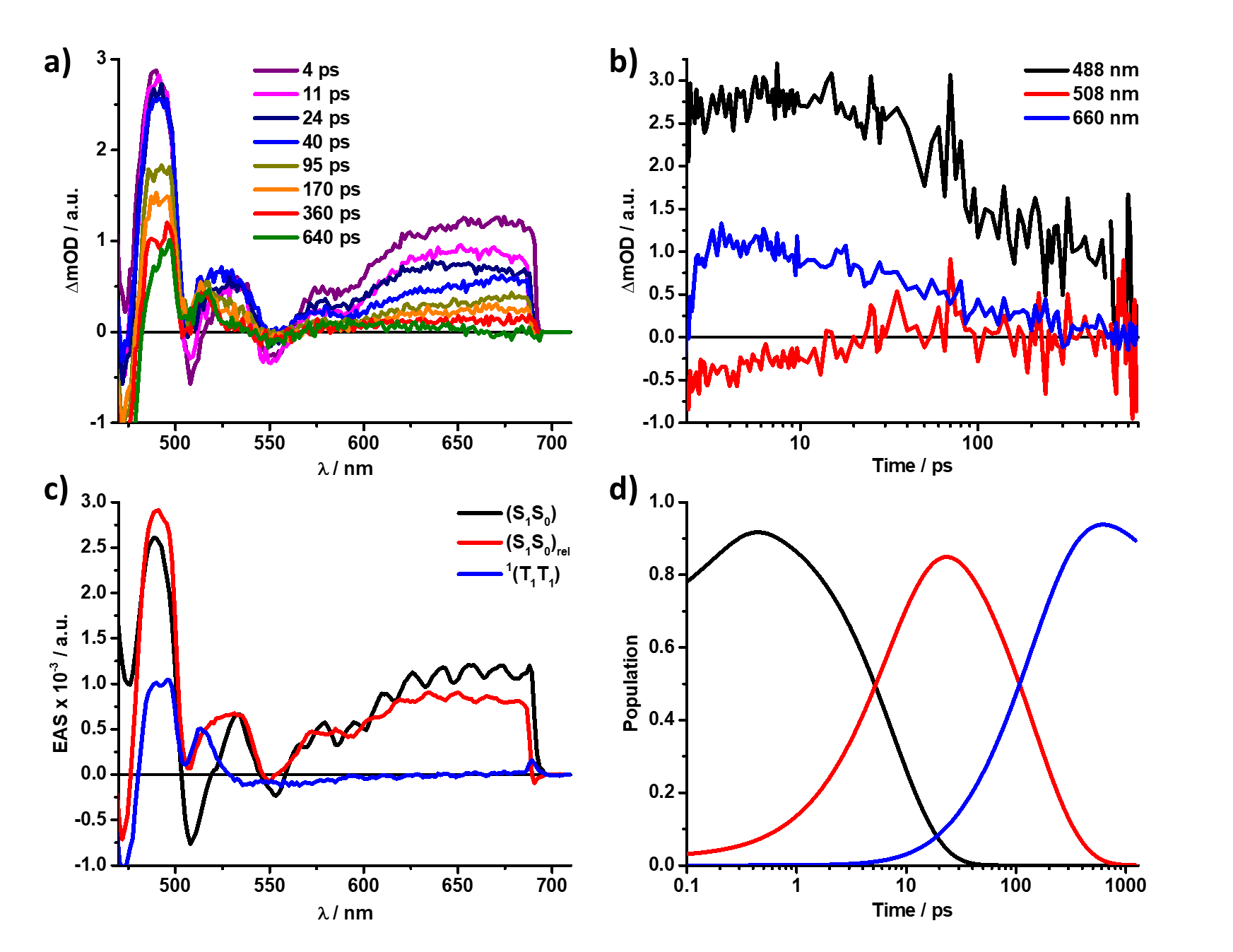


**Figure S47**. (a) Femtosecond transient absorption spectra (λ_ex_ = 400 nm) of aqueous **Tc_R‑NEA_NP** at the indicated time delays, together with (b) the respective time absorption profiles at the indicated wavelengths. (c) Deconvoluted evolution‑associated spectra (EAS) showcasing the initial hot singlet excited state (S_1_S_0_) (black), solvent and vibrational relaxed singlet excited state (S_1_S_0_)_rel_ (red) and singlet correlated triplet pair ^1^(T_1_T_1_) (blue) as obtained from global analysis. (d) Respective population kinetics of c).


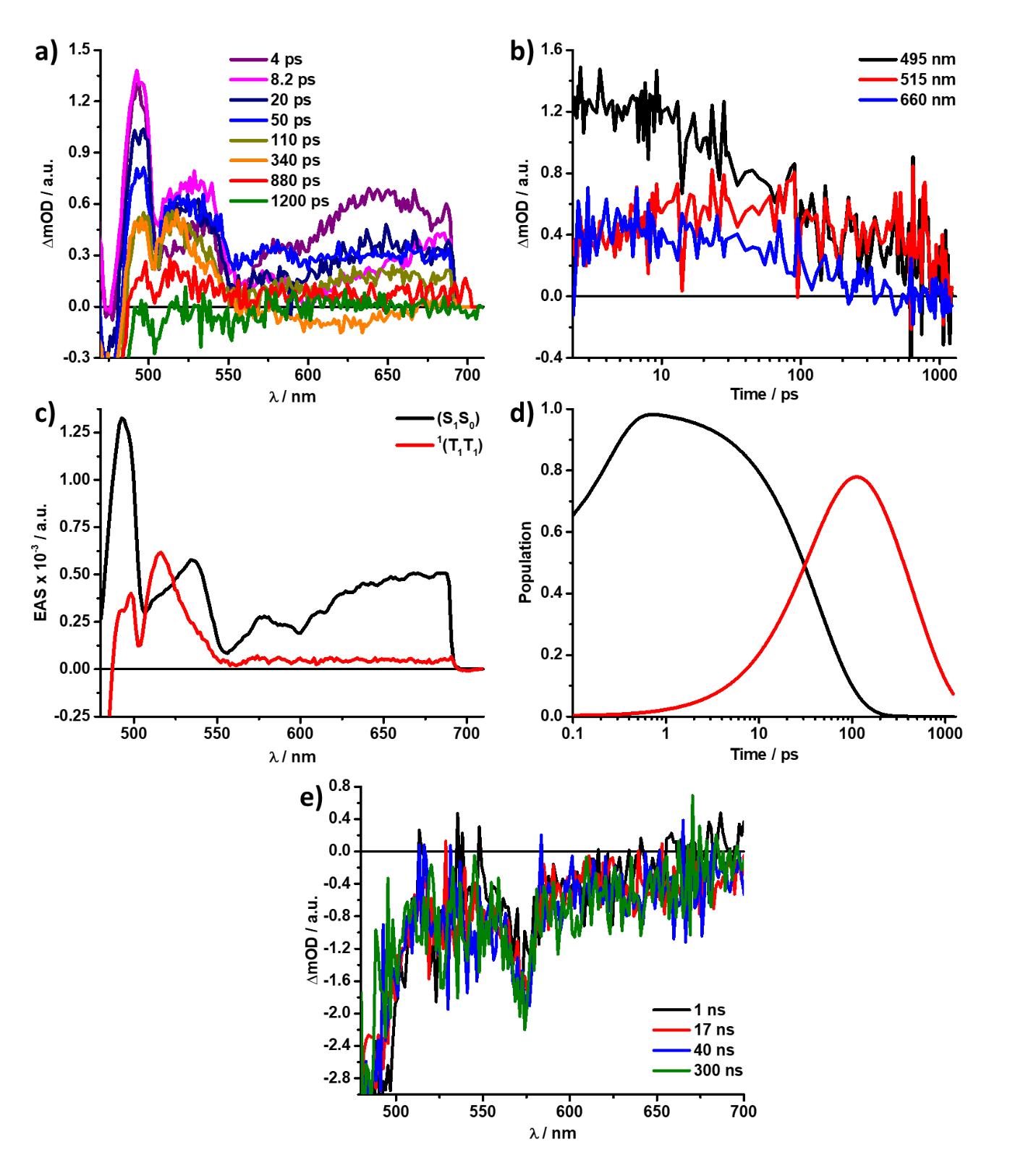


**Figure S48**. (a) Femtosecond transient absorption spectra (λ_ex_ = 400 nm) of aqueous **Tc_Rac_NP** at the indicated time delays, together with (b) the respective time absorption profiles at the indicated wavelengths. (c) Deconvoluted evolution-associated spectra (EAS) showcasing the singlet excited state (S_1_S_0_) (black) and singlet correlated triplet pair ^1^(T_1_T_1_) (red) as obtained from global analysis. (d) Respective population kinetics of c). (e) Corresponding nanosecond transient absorption spectra (λ_ex_ = 410 nm) at the indicated time delays, showcasing the absence of excited state absorption features on this timescale.


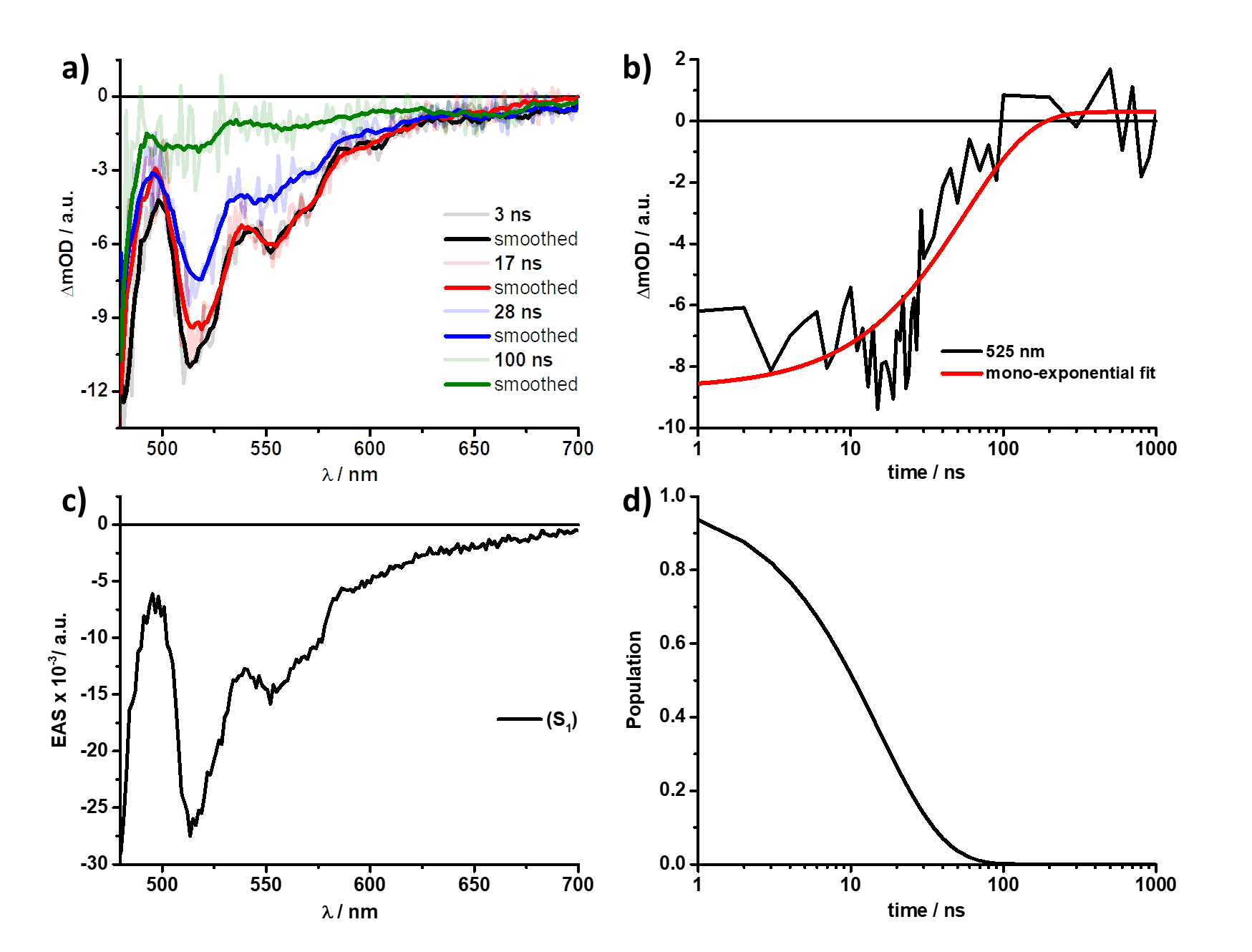


**Figure S49.** (a) Nanosecond transient absorption spectra (λ_ex_ = 410 nm) of aqueous **Tc_TPMA_NP** at the indicated time delays and (b) the respective time absorption profile and fit at the indicated wavelength. (c) Deconvoluted evolution-associated spectra (EAS) showcasing the singlet excited state (S_1_) (black) as obtained from global analysis. (d) Respective population kinetic of c). Due to the low stability of the nanoparticle solution, the signal-to-noise ratio was rather low.


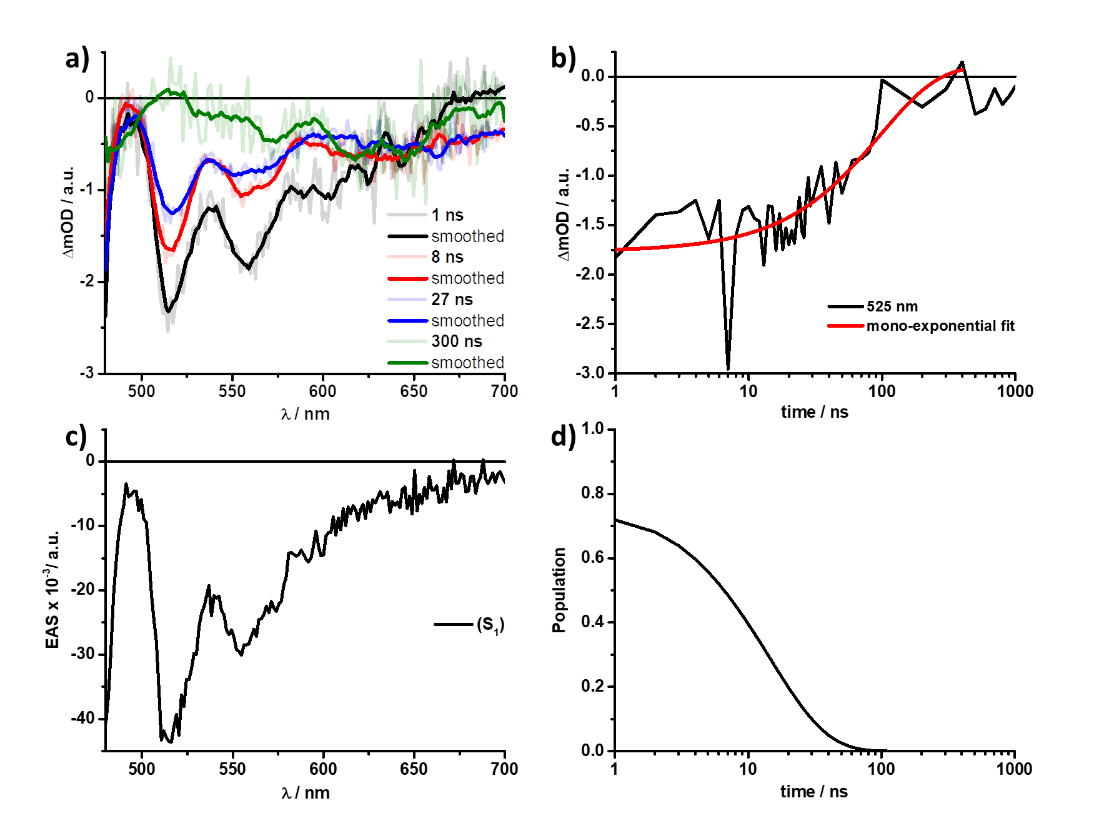


**Figure S50.** (a) Nanosecond transient absorption spectra (λ_ex_ = 410 nm) of aqueous **Tc_NMA_NP** at the indicated time delays and (b) the respective time absorption profile and fit at the indicated wavelength. (c) Deconvoluted evolution-associated spectra (EAS) showcasing the singlet excited state (S_1_) (black) as obtained from global analysis. (d) Respective population kinetic of c). Due to the low stability of the nanoparticle solution, the signal-to-noise ratio was rather low.


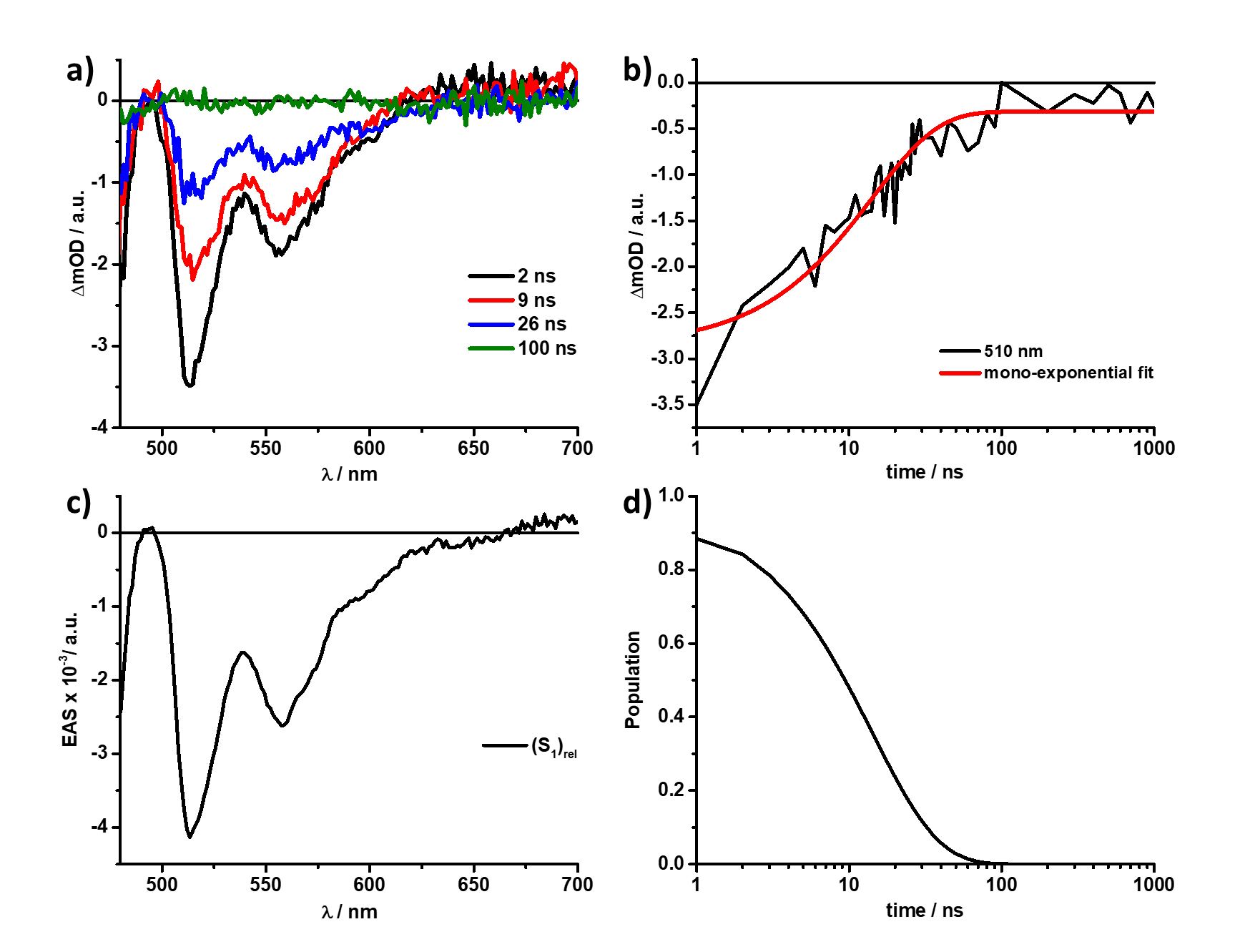


**Figure S51.** (a) Nanosecond transient absorption spectra (λ_ex_ = 410 nm) of **Tc_CyHx_NP** at the indicated time delays, together with (b) the respective time absorption profile and fit at the indicated wavelength. (c) Deconvoluted evolution-associated spectra (EAS) showcasing the relaxed singlet excited state (S­_1_)_rel_ (black) as obtained from global analysis. (d) Respective population kinetic of c).


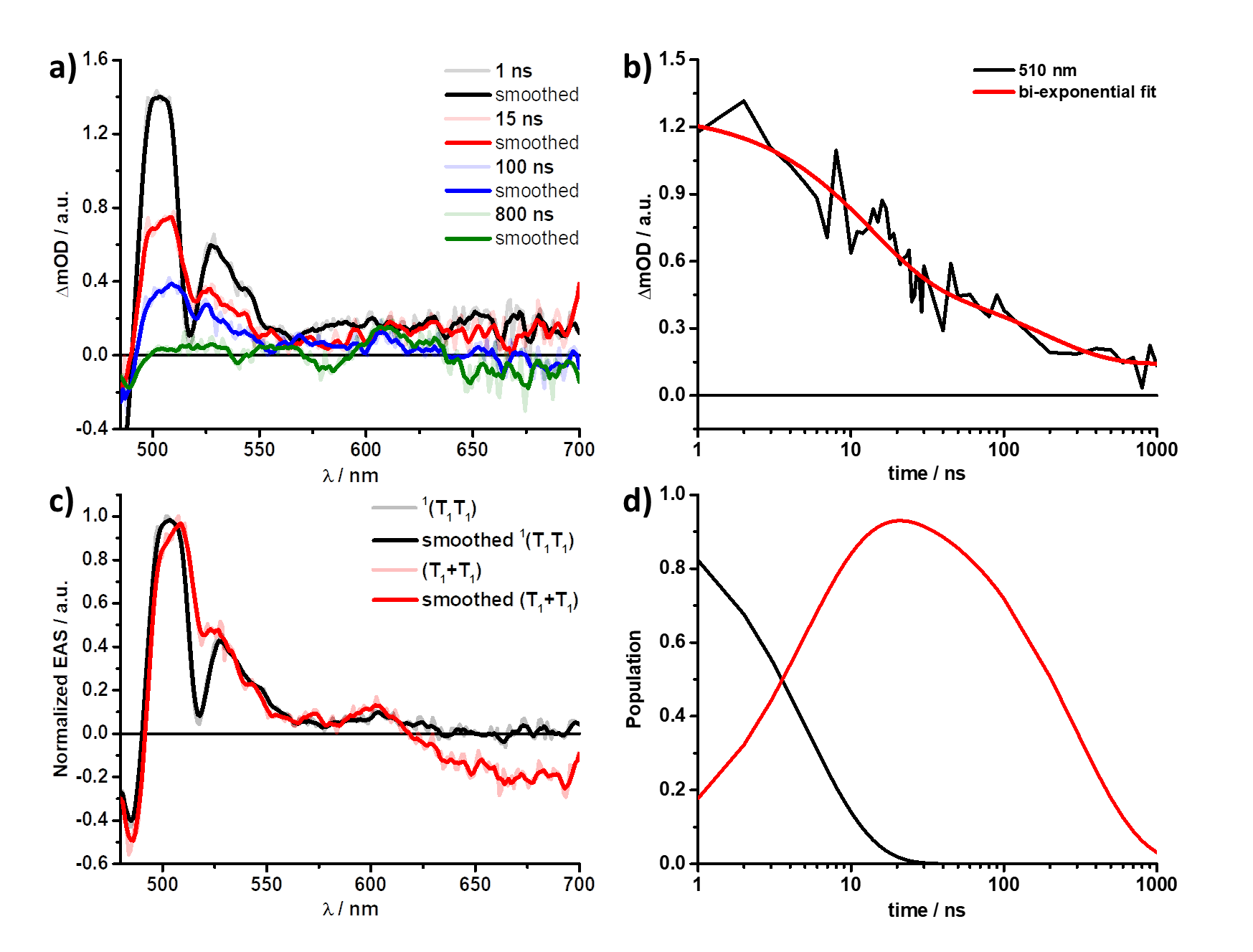


**Figure S52.** (a) Nanosecond transient absorption spectra (λ_ex_ = 410 nm) of **Tc_R‑NEA_NP** at the indicated time delays and (b) the respective time absorption profile and fit at the indicated wavelength. (c) Deconvoluted normalized evolution-associated spectra (EAS) showcasing the singlet correlated triplet pair ^1^(T_1_T_1_) (black) and uncorrelated triplet excited states (T_1_+T_1_) (red) as obtained from global analysis. (d) Respective population kinetics of c).

**Table S4.** Listing of the decay lifetimes from femtosecond and nanosecond transient absorption spectroscopy measurements of **Tc** and the respective Tc amine salts, **Tc_TPMA**, **Tc_NMA**, **Tc_CyHx**, **Tc_R‑NEA**, **Tc_S-NEA** and **Tc_Rac** in THF, together with the lifetimes of the respective nanoparticle solutions **Tc_TPMA_NP**, **Tc_NMA_NP**, **Tc_CyHx_NP**, **Tc_R‑NEA_NP**, **Tc_S-NEA_NP** and **Tc_Rac_NP**.

| System / Solvent | τ(S_1_) / ns |  |  |  |
| --- | --- | --- | --- | --- |
| Tc / THF | 11.75 |  |  |  |
| Tc_TPMA / THF | 11.99 |  |  |  |
| Tc_ NMA / THF | 13.75 |  |  |  |
| Tc_CyHx / THF | 11.81 |  |  |  |
| Tc_R-NEA / THF | 14.42 |  |  |  |
| Tc_S-NEA / THF | 12.88 |  |  |  |
| Tc_Rac / THF | 14.43 |  |  |  |
| Tc_TPMA_NPs / water | 15.13 |  |  |  |
| Tc_ NMA_NPs / water | 14.56 |  |  |  |
| System / Solvent | τ(S_1_) / ps | τ(S_1_)_rel_ / ns |  |  |
| Tc_CyHx_NPs / water | 17.00 | 14.19 |  |  |
| System / Solvent | τ(S_1_S_0_) / ps | τ(S_1_S_0_)_rel_ / ps | τ^1^(T_1_T_1_) / ns | τ(T_1_+T_1_) / ns |
| Tc_R-NEA_NPs / water | 7.55 | 143.44 | 5.10 | 287.24 |
| Tc_S-NEA_NPs / water | 4.53 | 157.08 | 4.35 | 208.31 |
| Tc_Rac_NPs / water | 43.35 |  | 0.45 |  |

**Triplet Quantum Yield Determination**

The triplet quantum yield (TQY) was determined using the bleaching method as described in the literature.^[44,45,52,53]^ In short, the TQY is directly proportional to the intensity of the ground state bleach (GSB) of (S_1_S_0_)_rel_ and ^1^(T_1_T_1_). As such, adequately scaled subtraction of the GSB can reproduce the pure GSB of (S_1_S_0_)_rel_ and ^1^(T_1_T_1_), respectively. The specific mathematic operation is to only add a specific amount of GSB to the (S_1_S_0_)_rel_ and ^1^(T_1_T_1_) spectra obtained from GloTarAn global analysis until the characteristic singlet and triplet excited state fingerprints are removed, in particular, the extremum at around 500 nm for (S_1_S_0_)_rel_ and at about 510 nm for ^1^(T_1_T_1_). Due to the scattering of the nanoparticle samples, and the ensuing differences and noise of the steady-state/transient absorption spectra, an error of ±20% and ±10% is considered for the evaluation of the ^1^(T_1_T_1_) and (T_1_+T_1_) TQYs, respectively.


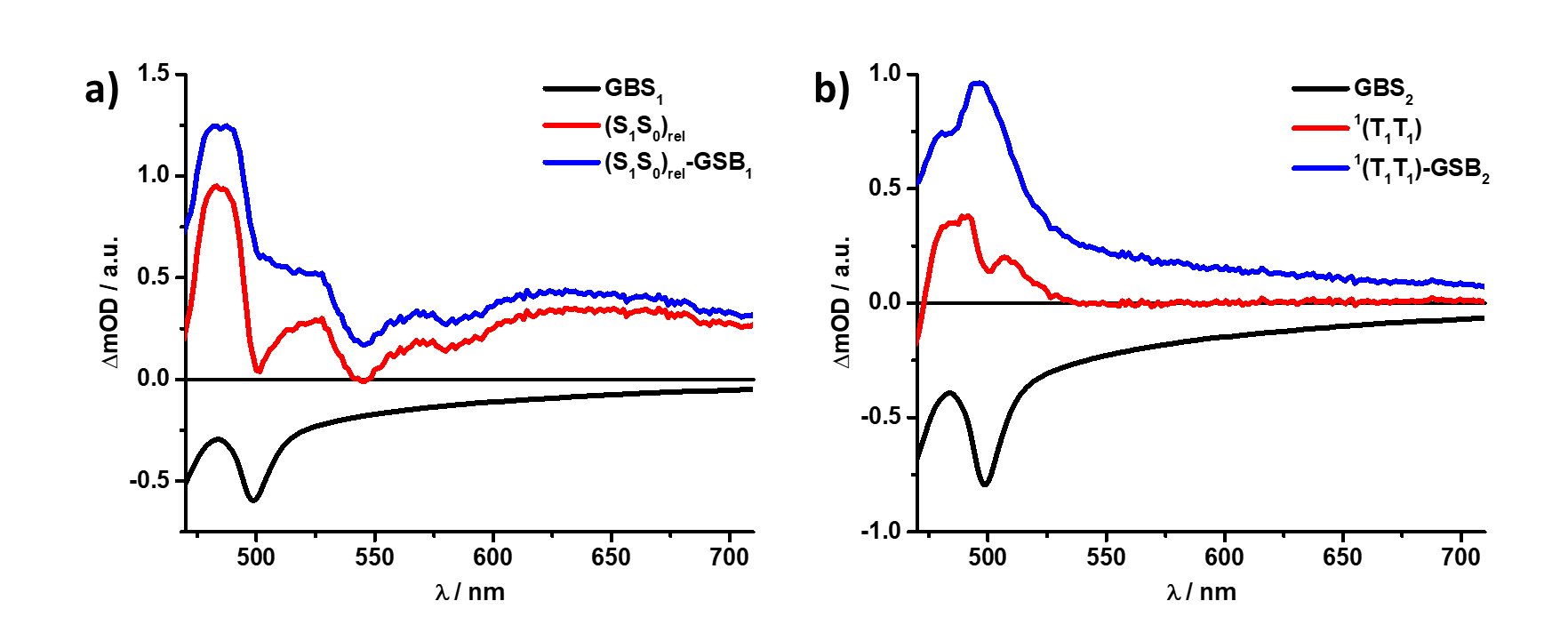


**Figure S53.** Transient absorption spectra of **Tc_S-NEA_NP** from **Figure 6**, showing a) (S­_1_S_0_)_rel_ and b) ^1^(T_1_T_1_) from GloTarAn global analysis (red, respectively), together with their pure spectra after the addition of GSB (blue, respectively) and scaled GSB (black, respectively).


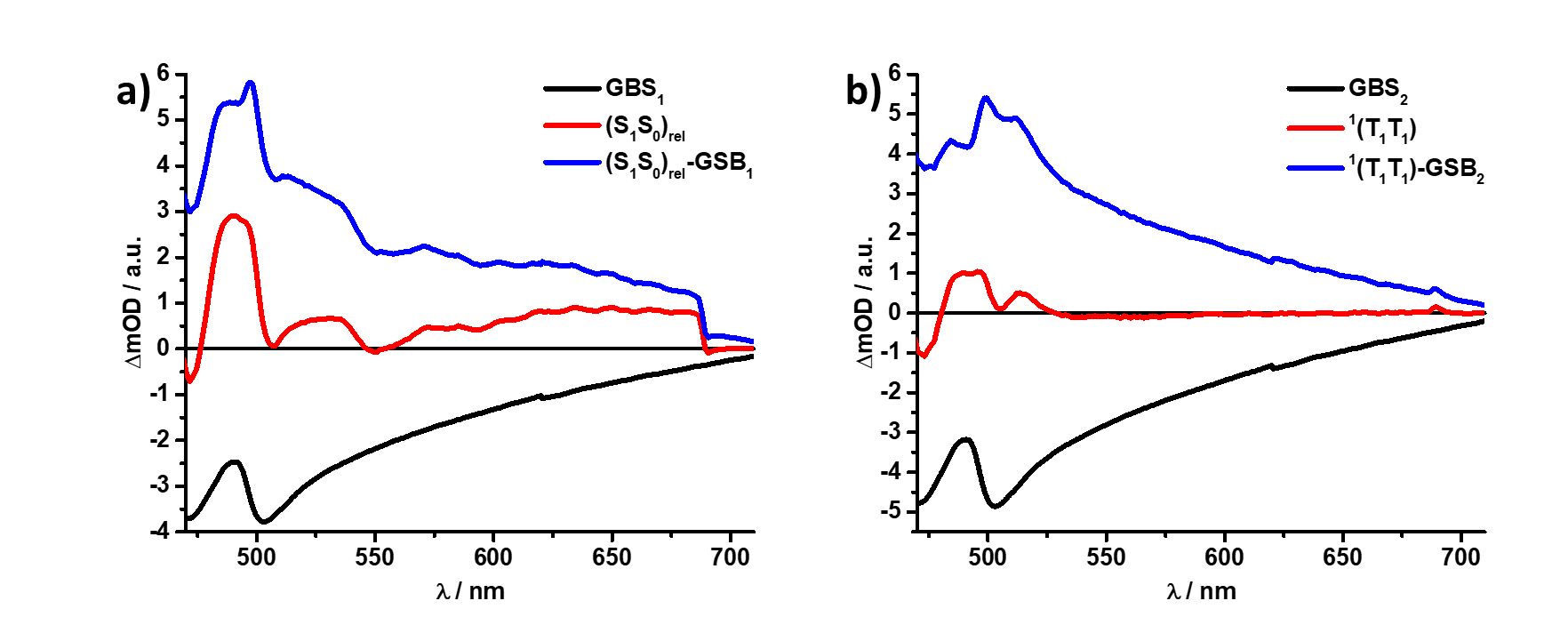


**Figure S54.** Transient absorption spectra of **Tc_R-NEA_NP** from **Figure S47**, showing a) (S­_1_S_0_)_rel_ and b) ^1^(T_1_T_1_) from GloTarAn global analysis (red, respectively), together with their pure spectra after the addition of GSB (blue, respectively) and scaled GSB (black, respectively).


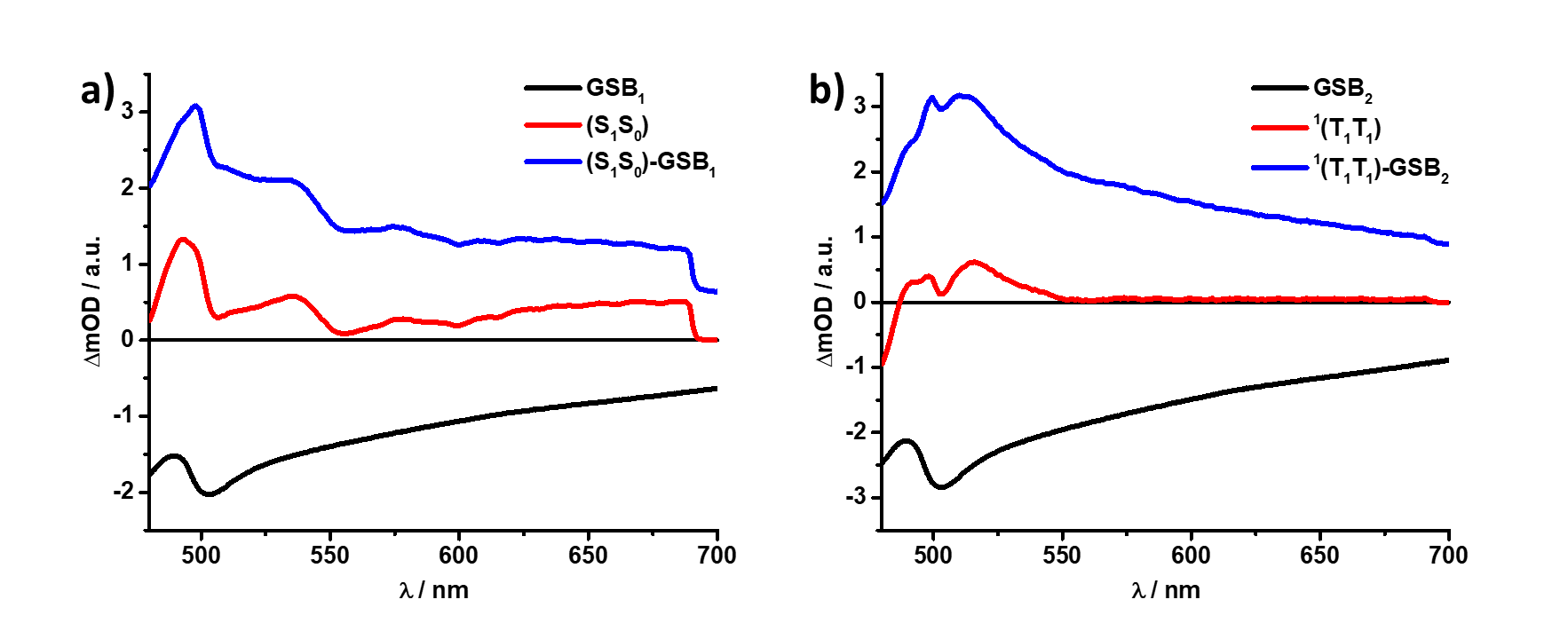


**Figure S55.** Transient absorption spectra of **Tc_Rac_NP** from **Figure S48**, showing a) (S_1_S_0_) and b) ^1^(T_1_T_1_) from GloTarAn global analysis (red, respectively), together with their pure spectra after the addition of GSB (blue, respectively) and scaled GSB (black, respectively).

The TQYs are then calculated from the respective GSB via the following equation:

$$TQY(\boldsymbol{Tc\_S}\boldsymbol{NEA\_NP}) = \frac{{GSB}_{2}}{{GSB}_{1}} x 100= \frac{-7.94E^{-4}}{-5.95E^{-4}} x 100=133\%$$

$$TQY(\boldsymbol{Tc\_R}\boldsymbol{NEA\_NP}) = \frac{{GSB}_{2}}{{GSB}_{1}} x 100= \frac{-4.86E^{-3}}{-3.78E^{-3}} x 100=129\%$$

$$TQY(\boldsymbol{Tc\_R}\mathrm{ac}\boldsymbol{\_NP}) = \frac{{GSB}_{2}}{{GSB}_{1}} x 100= \frac{-2.84E^{-3}}{-2.03E^{-3}} x 100=140\%$$

Furthermore, in order to calculate the yield of decoupled / free triplets, the same GSB method is applied to the ^1^(T_1_T_1_) and (T_1_+T_1_) states from GloTarAn global analysis of their respective nanosecond transient absorption measurements.


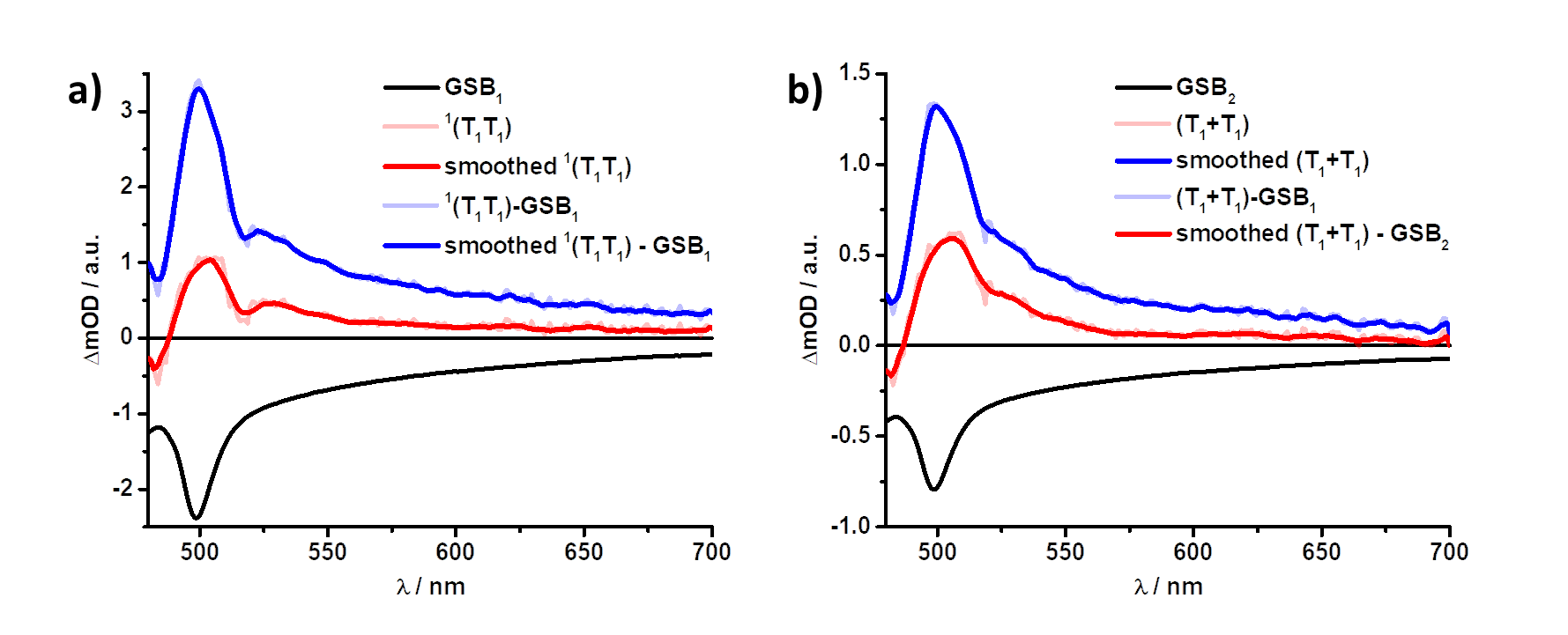


**Figure S56.** Transient absorption spectra of **Tc_S-NEA_NP** from **Figure 7**, showing a) a) ^1^(T_1_T_1_) and b) (T_1_+T_1_) from GloTarAn global analysis (red, respectively), together with their pure spectra after addition of GSB (blue, respectively) and scaled GSB (black, respectively).


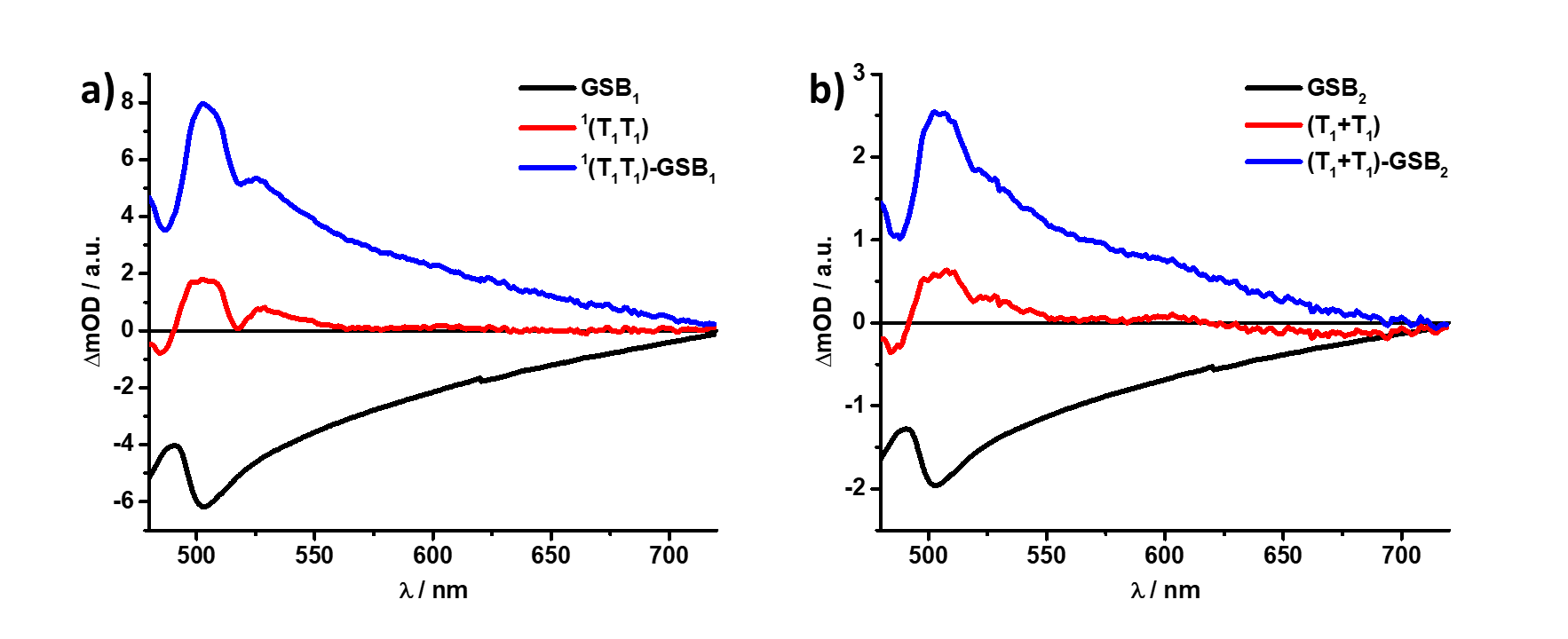


**Figure S57.** Transient absorption spectra of **Tc_R-NEA_NP** from **Figure S52**, showing a) a) ^1^(T_1_T_1_) and b) (T_1_+T_1_) from GloTarAn global analysis (red, respectively), together with their pure spectra after addition of GSB (blue, respectively) and scaled GSB (black, respectively).

The TQYs are then, again, calculated from the respective GSB via the following equation:

$$TQY(\boldsymbol{Tc\_S}\boldsymbol{NEA\_NP}) = \frac{{GSB}_{2}}{{GSB}_{1}} x 100= \frac{-7.938E^{-4}}{-2.3814E^{-3}} x 100=33\%$$

$$TQY(\boldsymbol{Tc\_R}\boldsymbol{NEA\_NP}) = \frac{{GSB}_{2}}{{GSB}_{1}} x 100= \frac{-1.960E^{-3}}{-6.173E^{-3}} x 100=32\%$$

**Table S5.** Summary of the FQYs and TQYs of **Tc_TPMA_NP**, **Tc_NMA_NP**, **Tc_CyHx_NP**, **Tc_R‑NEA_NP**, **Tc_S-NEA_NP** and **Tc_Rac_NP**.

| System | FQY / % | TQY (^1^T_1_T_1_) / % | TQY (T_1_+T_1_) / % |  |
| --- | --- | --- | --- | --- |
| Tc_TPMA_NPs / water | 43.5 | - | - |  |
| Tc_ NMA_NPs / water | 8.4 | - | - |  |
| Tc_CyHx_NPs / water | 10.1 | - | - |  |
| Tc_R-NEA_NPs / water | 6.4 | 129 | 32 |  |
| Tc_S-NEA_NPs / water | 7.9 | 133 | 33 |  |
| Tc_Rac_NPs / water | 2.0 | 140 | - |  |
